# Metagenomic characterization of ambulances across the USA

**DOI:** 10.1186/s40168-017-0339-6

**Published:** 2017-09-22

**Authors:** Niamh B. O’Hara, Harry J. Reed, Ebrahim Afshinnekoo, Donell Harvin, Nora Caplan, Gail Rosen, Brook Frye, Stephen Woloszynek, Rachid Ounit, Shawn Levy, Erin Butler, Christopher E. Mason

**Affiliations:** 1 Jacobs Technion-Cornell Institute, Cornell Tech, New York, NY USA; 2 Department of Physiology and Biophysics, Weill Cornell Medicine, New York, NY USA; 3 SUNY Downstate Medical Center, State University of New York, Brooklyn, NY USA; 4 Electrical and Computer Engineering, Drexel University, Philadelphia, PA USA; 5 School of Public Health and Health Sciences, University of Massachusetts, Amherst, MA USA; 6 Department of Computer Science and Engineering, University of California, Riverside, CA USA; 7 Hudson Alpha, Huntsville, AL USA; 8 The HRH Prince Alwaleed Bin Talal Bin Abdulaziz Alsaud Institute for Computational Biomedicine, Weill Cornell Medicine, New York, NY USA; 9 The Feil Family Brain and Mind Research Institute, Weill Cornell Medicine, New York, NY USA; 10 School of Medicine, New York Medical College, Valhalla, NY USA

**Keywords:** Ambulance, Classification, Taxonomy, Pre-hospital setting, Hospital-acquired infections, Nosocomial pathogens, Antimicrobial resistance, Microbial ecology, Metagenomics, Whole-genome sequencing

## Abstract

**Background:**

Microbial communities in our built environments have great influence on human health and disease. A variety of built environments have been characterized using a metagenomics-based approach, including some healthcare settings. However, there has been no study to date that has used this approach in pre-hospital settings, such as ambulances, an important first point-of-contact between patients and hospitals.

**Results:**

We sequenced 398 samples from 137 ambulances across the USA using shotgun sequencing. We analyzed these data to explore the microbial ecology of ambulances including characterizing microbial community composition, nosocomial pathogens, patterns of diversity, presence of functional pathways and antimicrobial resistance, and potential spatial and environmental factors that may contribute to community composition.

We found that the top 10 most abundant species are either common built environment microbes, microbes associated with the human microbiome (e.g., skin), or are species associated with nosocomial infections. We also found widespread evidence of antimicrobial resistance markers (hits ~ 90% samples). We identified six factors that may influence the microbial ecology of ambulances including ambulance surfaces, geographical-related factors (including region, longitude, and latitude), and weather-related factors (including temperature and precipitation).

**Conclusions:**

While the vast majority of microbial species classified were beneficial, we also found widespread evidence of species associated with nosocomial infections and antimicrobial resistance markers. This study indicates that metagenomics may be useful to characterize the microbial ecology of pre-hospital ambulance settings and that more rigorous testing and cleaning of ambulances may be warranted.

**Electronic supplementary material:**

The online version of this article (10.1186/s40168-017-0339-6) contains supplementary material, which is available to authorized users.

## Background

The vast diversity of microbial communities in our environment are shaped by many factors and have important implications for human health and disease. Recent advances in next-generation sequencing (NGS) and metagenomic analysis now enable us to map, quantify, and characterize environmental microbiomes and understand some of the factors shaping community composition and microbial population dynamics [1–3].

Microbial communities in the environment especially influence human health and disease in healthcare settings where patients often have increased susceptibility due to illness, invasive procedures, immunosuppression, or injuries [4, 5]. While metagenomics and other culture-independent research has been conducted in the healthcare environment [6–9], shotgun-based metagenomic sequence characterization of the ambulance pre-hospital setting is still an unexplored research area. The aim of this study is to use metagenomic techniques to profile the microbiome of ambulance surfaces across the country.

Ambulances and other pre-hospital settings are an important first point-of-contact between patients and hospitals. They also represent a vector for transmission of hospital-acquired infections (HAIs) to patients and healthcare workers and can conceivably represent a vector for transmission into hospitals [10]. Given the high rate of HAIs, with one in 25 hospital patients contracting infections [11], and increases in antimicrobial resistant (AMR) infections, there is an urgent need to characterize microbial populations in healthcare, hospital, and pre-hospital settings. There are multiple sources of nosocomial pathogens: important sources include patient’s endogenous microbiota and contamination from healthcare worker’s hands. Although more complex, over the last decade, the role of the surface environment as a source of nosocomial pathogens has also been increasingly acknowledged [12]. Targeted analyses of surface environments of ambulances are important because maintaining a sterile environment in this setting is challenging; furthermore, the cleaning regimen for ambulances is not as clearly defined or regulated as it is for other healthcare settings, such as in hospitals [13]. In fact, studies using culturing-based methods have found high incidence of *Staphylococcus aureus* and other potential nosocomial pathogens on ambulance surfaces [10, 14–18].

In addition to infectious disease and public health implications, studying the built environment using metagenomics also enables us to contribute to the field of microbial ecology. Elucidation of factors driving species diversity and distribution has historically been, and continues to be, a major focus of study in the field of ecology [19, 20]. With currently available sequencing technology and analysis tools, we are now able to explore these patterns at the microorganismal level in new ways, uncovering undiscovered levels of diversity and identifying novel microbial ecology dynamics [21, 22]. Metagenomics has been used to study the microbial ecology of a growing number of diverse environments including urban environments such as subways [2, 23], healthcare settings such as hospitals [7–9], built environments such as homes [1], and natural environments such as oceans [3]. Findings include evidence of overabundance of particular microbial populations associated with specific environments and taxa being driven by a myriad of factors such as surface-type, humidity, temperature, and cleaning regimens. Studies in built environments have shown characteristic microbial profiles often shaped by the unnatural environment and displaying evidence of selection by factors such as artificial chemicals and materials [22]. The metagenomics of ambulances may be of interest because ambulances nationwide can have divergent materials, design, and usage [13]; these mobile, built environments are distributed throughout the nation and thus allow for exploration of spatial and abiotic factors that may influence species diversity and distribution.

In this study, we used shotgun NGS sequencing (125 × 125 paired-end Illumina reads with > 99% base-level accuracy), on 398 surface samples collected from 137 ambulances in 19 cities in six states across the USA. Using a metagenomics analysis approach, we explored the following questions: (1) What is the microbial composition of ambulances and potential factors shaping this composition? (2) What is the functional characterization of these microbial communities (e.g., pathogenicity, and AMR markers) and what factors could be shaping this functionality? (3) What patterns of diversity are we seeing in these communities and what factors could be shaping this diversity? Overall, the aim of this study was to characterize the microbial ecology of ambulances across the USA using metagenomics.

## Results

**Fig. 1:**
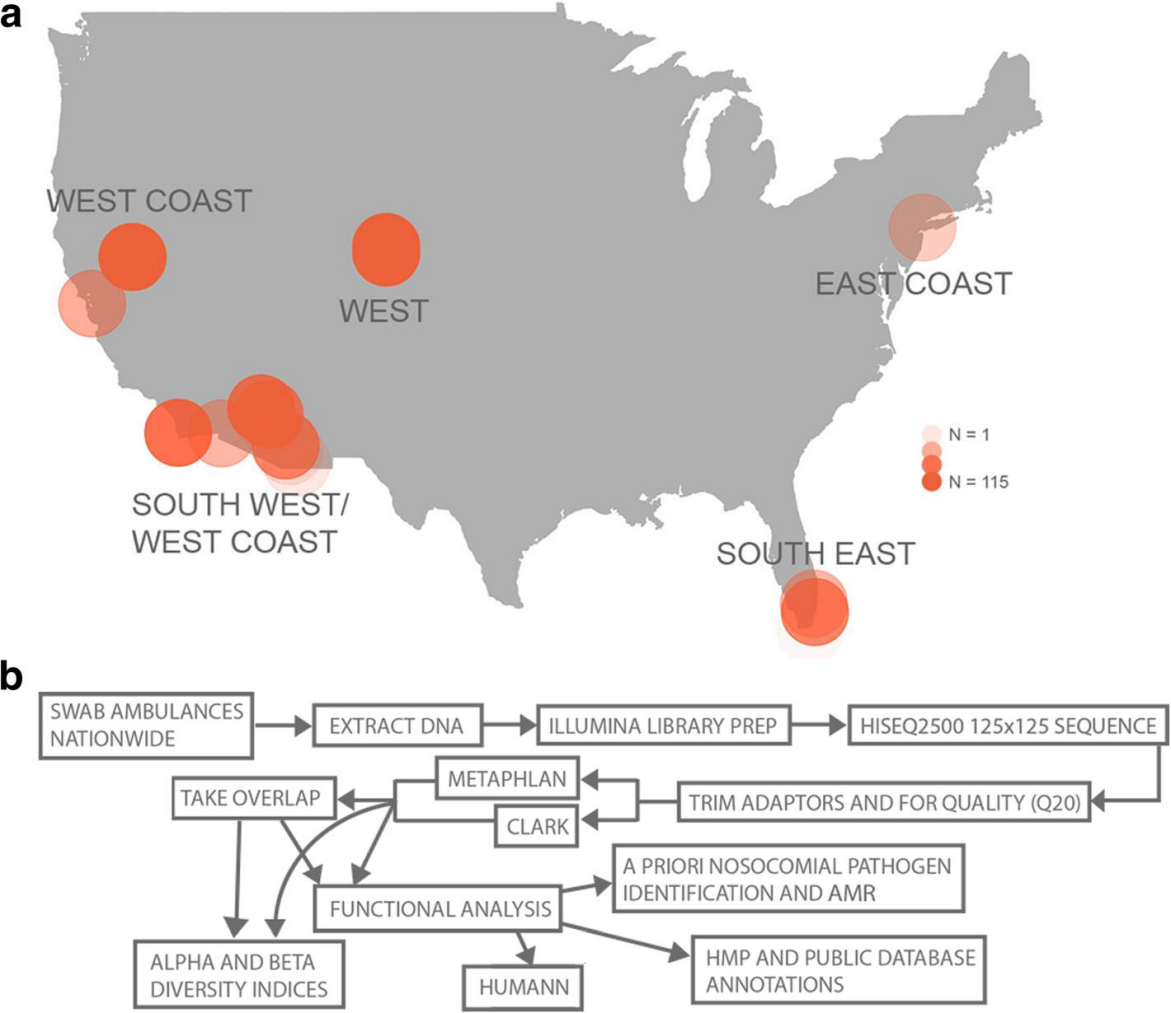
Sample collection and workflow. **a** Map of sample collection areas across the USA (cities not specified to protect privacy). Darker orange signifies a greater number of samples were collected as indicated in key. Sample collection was clustered in five regions labeled East, West, West Coast, Southwest/West Coast, and Southeast. **b** Workflow figure including laboratory and computational approaches used

Samples were collected by swabbing multiple surfaces using the international MetaSUB urban metagenomics protocol [24] within each ambulance including 3 min swab-based collections of computers, steering wheels, keyboards, medical equipment (stethoscopes, pulse O_2_ probes, blood pressure cuffs and bulbs, control panels, automated external defibrillators [AEDs], and monitors), stretchers, handles, rails, and cabinets. Samples were processed to extract DNA and 398 of the 1407 samples collected were sequenced. Samples sequenced were chosen to include all surfaces and breadth of locations (137 ambulances in 19 cities and six states; Fig. 1). Complementary classification tools Metagenomic Phylogenetic Analysis Tool (MetaPhlAn v2.0) [25] and CLARK [26] were used to classify samples and existing bioinformatics tools and custom scripts were used to further analyze these data (see Methods; Fig. 1). Statistical approaches including generalized linear models (GLMM) and random forest (RF) analysis were used to explore variables potentially shaping the microbial patterns observed. Finally, conducting a preliminary analysis using CLARK and default settings against a database of bacterial, viral and human genomes, we found that 2.6% (+/−6.5) of the reads per sample were human.

### Microbial identification and potential contributors

While several sequence classification methods are available to identify microorganisms in a sequenced sample, there is no computational method capable of absolute accuracy (i.e., no false positives and no false negatives). In the context of the healthcare environment, it is crucial to limit false positives/negatives in order to avoid reporting pathogens that are not present and missing pathogens that are truly present. To increase our absolute accuracy, we used classification tools MetaPhlAn2 and CLARK on the full dataset as well as an integrated multi-tool approach that generated the overlapping results of the two tools. MetaPhlAn2 performs better than many other state-of-the-art abundance estimation programs and can achieve zero false positives, and CLARK is one of most accurate read-based classifiers and is the only tool in studies showing the capacity for zero false negatives [27]. We tested this multi-tool approach by using synthetic datasets (see Methods) and found that combining results from both of these tools by taking the overlapping results (i.e., organisms that are identified by both tools for a given sample) can increase the detection accuracy of microorganisms (See Additional file 1: Table S1) in agreement with other studies (McIntyre et al. in press). We report and analyze both the individual MetaPhlAn2, CLARK, and overlap results as indicated in the methods and results and as outlined in Additional file 2: Table S2.

MetaPhlAn2 made 5119 species calls in total summing across all samples with 12.8 species/sample on average, while CLARK made a total of 39,015 species calls summing across all samples with 97.8 species/sample on average (Table 1; Additional file 3: Figure S1; Additional file 4: Table S3 all MetaPhlAn2 results; Additional file 5: Table S4 all CLARK results).

**Table 1:** MetaPhlAn2, CLARK, and MetaPhlAn2/CLARK overlap results. Count includes each time taxa was classified. Total count refers to counts summed across all samples

Tool	Total genera count	Total species count	Average genera count per sample (±SE)	Average species count per sample (±SE)
MetaPhlan2	5374	5119	13.47 (±0.46)	12.83 (±0.60)
CLARK	26,128	39,015	65.48 (±1.09)	97.78 (±1.65)
MetaPhlan2 and CLARK	4246	2644	10.64 (±0.35)	6.63 (±0.28)

This resulted in an overlapping dataset of 2644 species calls total, which represented 52.7% of the species calls by MetaPhlan2 and 6.8% of the species calls by CLARK. At the genus level, the overlap represents 79.0% of the genera calls by MetaPhlan2 and 16.3% of the genera calls by CLARK (Additional file 6: Figure S2). In agreement with other studies, CLARK had greater sensitivity and made more calls than MetaPhlan2 and there were some differences in taxa called due to variation in tool databases (McIntyre et al. in press). There were 127 unique species classified by both tools and the top 10 most abundant overlapping species were *Stenotrophomonas maltophilia, Pseudomonas stutzeri, Micrococcus luteus, Propionibacterium acnes, Enterobacter cloacae, Kocuria rhizophila, Pseudomonas putida, Bacillus cereus, Enterococcus faecalis,* and *Staphylococcus epidermidis*. Notably, this list includes species commonly associated with hospital-acquired infections or known to cause infections in immunocompromised hosts (Table 2). Despite these findings and associations, further analysis is necessary to elucidate whether these hits are in fact infectious agents. For the overlap species, the total relative abundance, average relative abundance, and standard error of the relative abundance are listed for all species identified by both tools with relative abundance determined by MetaPhlAn2 (Additional file 7: Table S5). There was variation in abundance and identification of species across cities, regions, and surfaces (Additional file 8: Figure S3). These overlapping species have been characterized further when data was retrievable (Additional file 9: Table S6) using sources including MicrobeWiki (https://microbewiki.kenyon.edu/index.php/MicrobeWiki) and the Human Microbiome Project (http://www.hmpdacc.org/).

**Table 2:** Top 10 most abundant species identified by MetaPhlan2 and CLARK (abundance from MetaPhlan2)

Species	Summed relative abundance across ambulances (average relative abundance ± SE)	NCBI Tax ID	Annotations
*Stenotrophomonas maltophilia*	2783.7 (7.0 ± 17.6)	40,324	A ubiquitous, aerobic, gram-negative bacterium. A common cause of nosocomial infections.
*Pseudomonas stutzeri*	2641.0 (6.63 ± 16.9)	316	A gram-negative soil bacterium found in almost all environments, it has diverse metabolic function, can fix nitrogen, and can be used in bioremediation and waste water treatment. It is an opportunistic pathogen, though rarely infects people.
*Micrococcus luteus*	1239.28615 (3.1 ± 13.7)	1270	A gram-positive, obligate aerobe which is part of mammalian skin microbiota and is also found in water, dust, and soil. Has been found to cause infections in immunocompromised patients.
*Propionibacterium acnes*	774.2 (1.9 ± 6.8)	1747	A gram-positive bacterium found on human skin and in the gastrointestinal tract and is linked to acne. Generally non-pathogenic but may contaminate bodily fluids and cause infections.
*Enterobacter cloacae*	400.0 (1.0 ± 6.4)	550	A gram-negative bacterium which is part of the normal gut microbiota which is an important nosocomial pathogen which causes a range of infections such as urinary tract and respiratory tract infections. Has been used as a biological control for plant disease.
*Kocuria rhizophila*	390.4 (1.0 ± 6.1)	72,000	A gram-positive bacterium with industrial applications in the food industry. Reclassified from *Micrococcus luteus* strain.
*Pseudomonas putida*	321.1 (0.8 ± 3.6)	303	A gram-negative soil bacterium which has a diverse metabolism that can degrade organic solvents and so has been used in bioremediation. It is found in soil and water habitats and is a type of rhizobacteria that forms a symbiotic relationship with host plants.
*Bacillus cereus*	199.4 (0.5 ± 5.2)	1396	A gram-positive aerobic bacterium found in soil and food. Some strains can cause food poisoning due to secretion of emetic toxins and enterotoxins. It is also an opportunistic pathogen.
*Enterococcus faecalis*	182.4 (0.5 ± 3.1)	1351	A gram-positive bacterium which can survive in harsh environments and is found in the gastrointestinal tract, in soil, water, and plants. It is a common cause of nosocomial infections, and harbors high levels of antibiotic resistance.
*Staphylococcus epidermidis*	148.4 (0.4 ± 2.2)	1282	A gram-positive bacterium part of the normal human skin microbiota but may cause infections in immunocompromised patients.

#### Surface and region classification

To explore how variables including surfaces, cities, and region may contribute to the variation in microbial communities observed, we used a machine learning approach to analyze these data. First, 20% of the data were randomly sampled and set aside for testing to assess generalizability. The remaining 80% were used as a training-validation test for repeated (10×) 10-fold cross validation. Using cross validation on the training-validation data, we performed parameters sweeps on an array of classifiers. We maximized classification performance of the taxonomic dataset by evaluating an array of classifiers on the training-validation set and found that random forest (RF) performed the best (mean ROC score across classes: surface = 0.618, region = 0.774). When using RF, our classification performance of surface was weak for both the MetaPhlAn2 (0.6354) and overlap (0.629) datasets, but we were nonetheless able to effectively classify region of the USA based on these data, particularly for the MetaPhlAn2 data (0.787) (see Additional files 10, 11, 12, 13, 14, 15, 16, 17, 18 and 19).

**Fig. 2:**
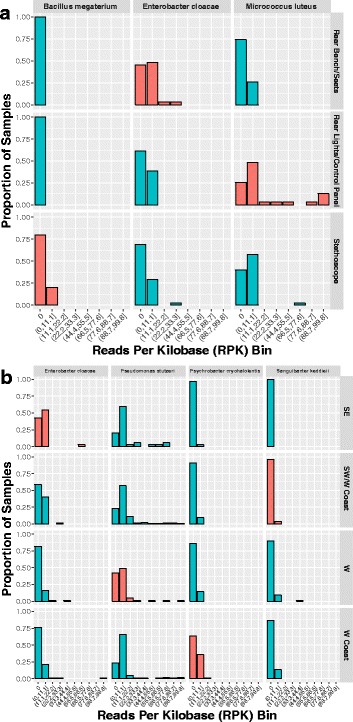
Top ranking features (species) during random forest classification training (128 trees) when the overlap dataset was used. Features were identified in terms of random forest importance scores, indicating their contribution to classification performance for a given class. The relative abundances (RPK) for each top ranking feature across all samples were binned (*x*-axis). The frequency of each feature across samples falling into these bins is shown (*y*-axis). Bars shaded red indicate the highest ranking feature for a given class. High ranking features with large frequencies at bin 0 suggest that those features are rare, but if present, highly influence the classifier to classify a sample in that feature’s corresponding class. **a** Surface. **b** Region

To identify the taxa most influential in distinguishing between classes, we ranked the taxa based on RF feature importance. The results for the 10 surfaces are shown in Additional file 20: Figure S4. Rear Bench Seats, Rear Lights Control Panel, and Stethoscope are best distinguished by *E. cloacae*, *M. luteus,* and, to a lesser degree, *Bacillus megaterium*, respectively (Fig. 2). Moreover, presence/absence of *E. cloacae* and *B. megaterium* seemingly had more impact on classifier performance than differences in their relative abundance between samples. For example, 17/31 Rear Bench Seats samples contained *E. cloacae*, compared to 107/365 of samples from other surfaces; however, of the 17 sites containing this species, the mean normalized abundance (from MetaPhlAn2) was only 4.19. The result was similar for *B. megaterium* with respect to stethoscope samples, with 9/45 (mean = 1.07) compared to 7/351 non-stethoscope samples containing the species. *M. luteus* was similarly found in a greater proportion of Rear Lights Control Panel samples (23/31) compared to other surfaces (156/365), but at much larger abundances (mean = 24.36). This suggests that the very presence of certain taxa significantly influenced classification performance, even if the abundance of that taxa was low.

The same approach was repeated for region and city classes. The RF region model performed considerably well during cross validation with a mean ROC score and balanced accuracy across classes of 0.8750 and 0.7789, respectively. Performance was best for the classes with fewest samples (Southeast and West, 0.7452 combined accuracy), which were up-sampled during cross validation. This was likely because we resampled these minority classes during cross validation to overcome issues with unbalanced class sample sizes.

To assess generalizability, we adjusted our approach for creating a test set. Specifically, we split our regions in terms of city, such that the within-region cities in the test set were not utilized during training. We believe this approach should help prevent the classifier from seeing similar co-occurrence profiles in the test set that may be from the same ambulance. The test set class sizes were balanced so 8 samples would be used for each region class. The average accuracy across all 4 classes was 0.438 ([0.263, 0.623], via the Clopper-Pearson CI approach) (Southeast = 3/8, Southwest/West Coast = 5/8, West = 4/8, West Coast = 2/8). Mean balanced accuracy, F1, and AUC were 0.625, 0.448, and 0.698, respectively.

Given our concern with the size of our test set, we wanted to assess the consistency of test set performance using different combinations of samples and cities, but again maintaining the use of cities in the test set that were not trained on. We performed a Monte Carlo simulation, sampling without replacement the city and samples for the test set and then performing classification as described above. For each class, we sampled 1% of samples for each class for our test, giving us 40 in total (Southeast = 4, Southwest West Coast = 13, West = 10, West Coast = 13). We calculated the overall testing performance across 1000 runs. There was a drop in balanced accuracy for each class: Southeast = 0.563, Southwest/West Coast = 0.559, West = 0.598, West Coast = 0.541. This suggests that interpretation of the important features should be performed with caution, particularly when extrapolating to hypothetical new data. Still, given our study’s limitations in terms of sample size (both overall and within-class), our analysis indicates that we were able to effectively classify region.

When the US’s regions were split into city classes, performance declined, likely due to smaller sample size for each class. This performance drop was reflected by the decrease in the ability of the RF to classify the held out test set, used after cross validation. This suggested an inability of the RF to generalize well (mean ROC = 0.6326, mean balanced accuracy = 0.5857), despite good performance during cross validation (mean ROC = 0.9212, mean balanced accuracy = 0.7949).

RF importance rankings for region and the frequencies of these features across samples are shown in Fig. 2 and Additional file 21: Figure S5. The rankings suggest multiple species influenced the classification of the RF for a given class. This is particularly clear with the top ranking Southwest/West Coast feature being more abundant in West and West Coast samples. For city, on the other hand, specific species more intimately associate with samples as a function of class (Additional file 22: Figure S6 and Additional file 23: Figure S7). *Erwinia billingiae*, *Klebsiella pneumoniae*, and *Psychrobacter arcticus* are generally rare except in S004, S006, and S003 samples, respectively. Also, while *S. maltophilia* occurs in multiple samples across cities, samples from city S019 are especially dominated by large abundances of this species.

### Functional characterization of ambulance microbial communities and potential contributors

#### Functional analysis using HUManN2

**Fig. 3:**
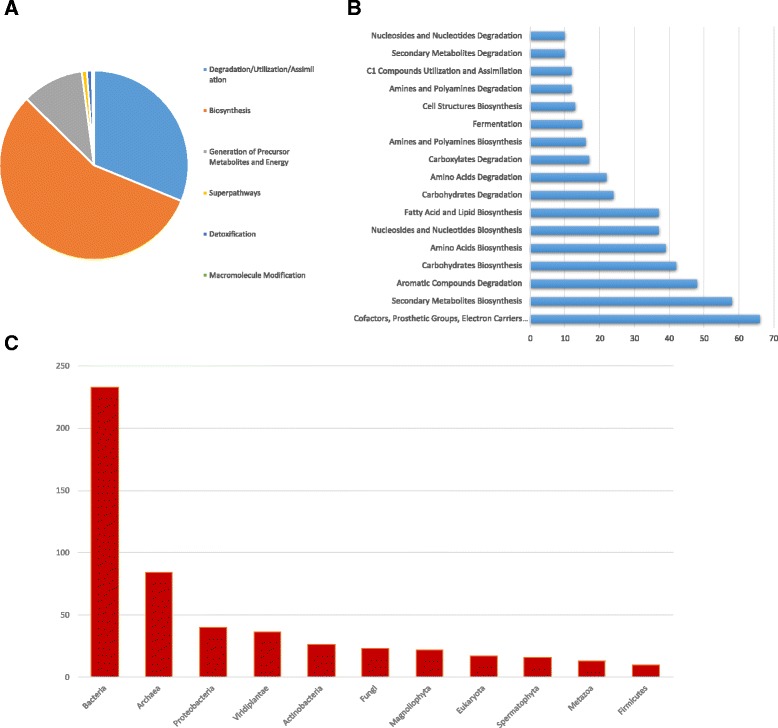
HUMAnN2 functional analysis results. Breakdown of superclasses of pathways identified and their relative proportions across the entire dataset (**a**), number of hits for top pathways identified across the entire dataset (**b**), and number of hits for different taxa across the entire dataset (**c**). All results determined from the annotations posted on the MetaCyc database for each identified pathway

Functional genomic profiles of the full dataset were generated using HUMAnN2 [version 0.5.0; 28; http://huttenhower.sph.harvard.edu/humann2]. HUManN2 identified 578 pathways from the MetaCyc database across our dataset. Most of these pathways were associated with bacterial organisms, further supporting our taxa classification results by MetaPhlAn2 and CLARK (Fig. 3). Annotations from the online MetaCyc database revealed that the top functional pathways superclass include Biosynthesis, Degradation, Utilization, and Assimilation, and finally, Generation of Precursor Metabolites and Energy. More specifically, the top pathways included biosynthesis of cofactors, prosthetic groups, and electron carriers, as well as secondary metabolites biosynthesis, and aromatic compound degradation. For a full list of pathways divided into superclass and categories based on MetaCyc annotations please see Additional file 24: Table S7.

Using the HUManN2 functional classification results, we performed a differential functional abundance analysis between the following classes, chosen based on adequate sample size and performance during RF classification with the overlap dataset: Stethoscope, Rear Lights Control Panel, and Rear Bench Seats for surfaces; Southeast, Southwest/West Coast, and West Coast for region; and S005, S003, S002, and S007 for city. Within each class category, a DESeq2 analysis was performed for each unique class combination, arbitrarily designating one of the classes as the reference class.

Additional file 25 Figure S8 shows volcano plots of surface *p* values after FDR correction versus log_2_ fold change (LFC) of functional pathway abundance. Despite an even distribution of pathway superclasses, several were significantly more abundant in Stethoscope compared to Rear Bench Seats, with 25% of the pathways in the upper LFC 95th percentile involved in aromatic compound degradation. There were notably few differentially abundant pathways between Stethoscope and Rear Lights Control Panels, however.

In terms of region (Additional file 26: Figure S9), Western samples tended to have far fewer differentially abundant pathways, which could be attributed to its lower levels of alpha diversity relative to the other three regions (Additional file 27: Figure S10) and our observation that taxonomic prevalence (proportion of taxa present in a given sample) is positively correlated with functional prevalence (Additional file 28: Figure S11). When regions are split into city classes, far fewer differentially abundant pathways result, likely due to small and unbalanced sample sizes (Additional file 29: Figure S12). A complete list of the LFC of functional pathway abundances for the three aforementioned class categories can be found in the supplementary material.

#### Microbial association with human microbiome

**Fig. 4:**
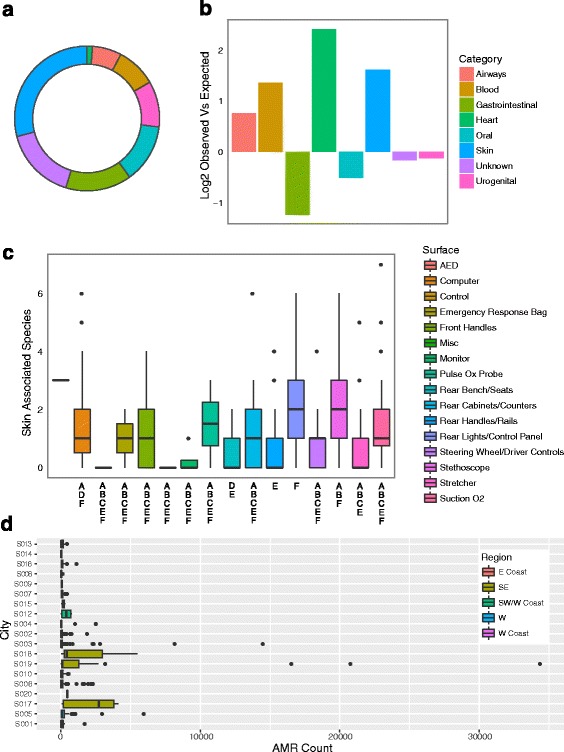
Functional analysis including Human Microbiome Project annotated ambulance species for overlap results and AMR hits. **a** Proportions of species identified in ambulances associated with indicated human body parts. **b** Deviation of ambulance body part associations from HMP database indicates HMP proportions are not driving patterns observed in ambulances and that heart, skin, and blood associated species are overrepresented. **c** Skin associated species varied significantly across surfaces, shared letter(s) on the *x*-axis between surfaces indicates statistical equivalence. **d** Boxplot of AMR hits across cities with boxplots colored by region

We next annotated our MetaPhlAn2/CLARK overlapping dataset with a Human Microbiome Project (HMP) dataset collected from healthy individuals (http://www.hmpdacc.org/HMRGD/healthy/#data, downloaded August 11 2016) to characterize identified species’ association with specific regions of the human body. We found that about half the species identified by both MetaPhlAn2 and CLARK were in the HMP database with the greatest proportion of these microorganisms being associated with skin, followed by an unknown primary site association (but present in the database), and then by gastrointestinal tract (Fig. 4). As a control, we found that the proportions of body part categories in the HMP database were not driving the proportions we were seeing in the ambulance (chi-square test of independence: *X* ^*2*^ = 421.71, Df = 9, *P* < 2.2 × 10^−16^). To determine deviation in ambulance proportions to HMP database proportions, we took the log_2_ of observed versus expected results. After heart, which had the largest difference between the observed and expected, but had an unacceptable sample size in the database (*N* = 2), we found that skin associated species were the most abundant in ambulances and the most overrepresented. After skin, blood was the third most overrepresented compared to the database and was also highly abundant. Finally, there were less gastrointestinal and oral microbes observed than expected given the database size (Fig. 4).

We used non-parametric tests to explore whether different variables may be driving the proportions of body part categories observed. We found that skin associated species varied significantly across surfaces (Kruskal-Wallis *X* ^*2*^ = 62.293, Df = 15, *P* = 1.013 × 10^−7^). We did a post hoc Kruskal test (Nemenyi test in R with built-in multiple correction) and found that rear handles rails versus computer (*P* = 0.034), rear bench seats versus rear lights control panel (*P* = 0.004), rear lights control panel versus rear handles rails (*P* = 0.010), stethoscope versus rear bench seats (*P* = 0.001), stethoscope versus rear handles rails (*P* = 0.003), and stretcher versus rear lights control panel were all significantly different (*P* = 0.030)(Fig. 4).

#### HAI-related pathogen and AMR distribution

In profiling the samples using MetaPhlAn2 and CLARK, using an a priori approach, many potential HAI-related pathogens were identified. For this analysis, we designate HAI-related pathogens as known pathogens previously characterized as causing greater than 1% of reported HAIs [28]. Because it is still challenging to resolve some pathogens at the species or strain level, we annotated at both the genus and species levels. Our results showed many hits for genera that include top nosocomial pathogenic species, with 341/398 (85.7%) of samples identified to contain HAI-associated genera identified by both MetaPhlAn2 and CLARK. Furthermore, we found that 312/398 (78.4%) of samples were identified to contain at least one nosocomial infection-related species identified by both MetaPhlAn2/CLARK (Table 3).

**Table 3:** Most common causes of HAIs [Magill 2014 and characterized further [57, 58] and hits in our ambulance samples

Species	Types of infections	Ambulance hits MetaPhlAn2	Ambulance hits overlap
*Clostridium difficile*	GI	0	0
*Staphylococcus aureus*	Pneumonia, surgical site, bloodstream	15	15
*Klebsiella pneumoniae*	Pneumonia, surgical site, UTIs	12	12
*Klebsiella oxytoca*	Pneumonia, surgical site, UTIs	6	6
*Escherichia coli*	Surgical site, UTIs, bloodstream	0	0
*Enterococcus faecalis*	Surgical site, UTIs, bloodstream	56	56
*Enterococcus faecium*	Surgical site, UTIs, bloodstream	38	38
*Enterococcus avium*	Surgical site, UTIs, bloodstream	1	0
*Pseudomonas aeruginosa*	Pneumonia, surgical site, UTIs	26	26
*Candida albicans*	Pneumonia, UTIs, bloodstream	0	0
*Candida parapsilosis*	Pneumonia, UTIs, bloodstream	0	0
*Candida glabrata*	Pneumonia, UTIs, bloodstream	0	0
*Candida dubliniensis*	Pneumonia, UTIs, bloodstream	0	0
*Streptococcus pneumoniae*	Pneumonia, surgical site, bloodstream	0	0
*Streptococcus parasanguinis*	Pneumonia, surgical site, bloodstream	18	18
*Acinetobacter baumannii*	Pneumonia, surgical site	8	8
*Proteus mirabilis*	Pneumonia, surgical site, UTIs	0	0
*Stenotrophomonas maltophilia*	Pneumonia, UTIs	280	280
*Mycobacterium tuberculosis*	NA	9	6
Genus			
*Enterococcus*	Surgical site, UTIs, bloodstream	114	114
*Candida*	Pneumonia, UTIs, bloodstream	0	0
*Streptococcus*	Pneumonia, surgical site, bloodstream	52	52
*Enterobacter*	Surgical site, UTIs, bloodstream	125	125
*Aspergillus*	NA	2	0
*Fusarium*	NA	39	0
*Scedosporium*	NA	0	0
*Citrobacter*	NA	0	0
*Serratia*	NA	8	8
*Bacteroides*	NA	1	1
*Haemophilus*	NA	25	25

Column one is pathogens included that cause at least greater than 1% of HAIs, column two lists types of infections (from Magill 2014 includes up to top three types of infections due to pathogen), and column three and four list the number of hits identified in ambulance samples for nosocomial taxa (species and genera) identified by MetaPhlAn2 and identified by both MetaPhlAn2 and CLARK (overlap)

Of the 18 top putative nosocomial pathogen species, 10 (56%) were identified in our ambulance samples by MetaPhlAn2 and 9 (50%) were identified by both MetaPhlAn2 and CLARK (Table 3). These include *S. aureus*, *K. pneumoniae*, *Klebsiella oxytoca*, *E. faecalis*, *Enterococcus faecium*, *Enterococcus avium* (only identified by MetaPhlAn2), *Pseudomonas aeruginosa*, *Streptococcus parasanguinis*, *Acinetobacter baumannii*, and *S. maltophilia*. While not on the list, *Mycobacterium tuberculosis* was also classified in 9 (2.3%) samples. Many of the genera and species identified commonly harbor antibiotic resistance, including *S. aureus* and *E. faecalis*.

There is widespread interest in the incidence of *S. aureus* in hospitals and ambulances. We determined if sequence coverage across *S. aureus* was sufficient to reliably report the potential pathogen and to test for evidence of methicillin resistance. We explored sequence coverage (Additional file 30: Table S8, Additional file 31: Figure S13) across the genome of all 15 samples identified as *S. aureus* positive by MetaPhlan2 and CLARK to check identification and check for antibiotic resistance by calculating coverage over *femA*, *femB* (used to characterize level of methicillin resistance), and *SCCmec* (including *mecA*), which is commonly used to identify and characterize methicillin resistant *S. aureus* (MRSA) [29, 30]. Using this analysis of coverage, we found evidence to support the identification of *S. aureus* by MetaPhlAn2/CLARK with consistent coverage across the genomes, but a lack of evidence for MRSA, with very little coverage over *mecA* (Additional file 30: Table S8, Additional file 31: Figure S13). However, we did find evidence of other *S. aureus* associated AMR markers as detailed below.

We also analyzed potential presence of AMR markers in the full dataset by building a custom CLARK database using the Comprehensive Antibiotic Resistance Database (CARD) sequence files [31]. We found that 289 of the 2172 markers in the CARD database had hits in our samples. The top hits were associated with known high priority nosocomial pathogens including *S. maltophilia*, *S. aureus*, *P. aeruginosa*, *E. coli*, and *E. cloacea* (Additional file 32: Table S9). Evidence for AMR was commonly found in our samples with 95.7% of the samples having at least one AMR hit and 89.5% of samples having at least three AMR hits. In regard to the high priority *S. aureus* pathogen, we found a number of AMR markers with high abundance in the samples. The top 10 most abundant *S. aureus* associated AMRs in the CARD database included *mecR1*, *qacA*, *blaZ*, *tetK*, *AAC(6′)-le-APH(2″)-la*, *mecI*, *sav1866*, *tet38*, *mepA*, *dfrG*. These results provide evidence for possible antimicrobial resistant *S. aureus* in these ambulance populations; however, further studies are warranted to test resistance.

When modeling the total number of AMR hits per sample using a univariate approach, we found a significant difference in AMR counts in different regions of the country, with Southeastern ambulances having by far the highest level of AMR hits (ANOVA on log transformed AMR count data with East Coast dropped to only included regions with *N* > 10: *F*_3,386_ = 14.94, *P* = 3.22 × 10^−9^; Fig. 4). We also saw a marginally significant difference in AMR hits across surfaces (ANOVA on log transformed AMR count data with AED and emergency response bag, monitor, miscellaneous, pulse ox probe dropped to exclude surfaces with *N* < 10: *F*_10,377_ = 2.16, *P* = 0.02). In agreement with our analysis of the effect of region on AMR, we found an anti-correlation between latitude and AMR count, with lower, southern latitudes having a greater AMR count (*t* = − 4.90, df = 395, *P* = 1.43 × 10^−6^, *r* = − 0.24) and a positive correlation between longitude and AMR count with samples collected further east having a higher AMR count (*t* = 5.72, df = 395, *P* = 2.15 × 10^−8^, *r* = 0.27). In regard to potential weather variables, we saw a positive correlation between temperature and AMR count with higher mean temperatures having higher AMR counts (*t* = 4.57, df = 395, *P* = 6.45 × 10^−6^, *r* = 0.22) and a positive correlation between precipitation and AMR counts with higher precipitation being associated with higher AMR counts (*t* = 5.67, df = 395, *P* = 2.81 × 10^−8^, *r* = 0.27). Alpha diversity and AMR counts were positively correlated with more diverse samples having a higher AMR count (*t* = 4.67, df = 393, *P* = 4.18 × 10^−6^, *r* = 0.23). We found a strong positive correlation between AMR count and top HAI-causing pathogen count (*t* = 8.18, df = 395, *P* = 4.00 × 10^−15^, *r* = 0.38). Finally, we found a strong correlation between AMR count and *S. maltophilia* abundance, which is also in agreement with the highest counts being identified in CARD database as being associated with *S. maltophilia* and indicates that our classification is matching up with the CARD species associated AMR (*t* = 11.47, df = 395, *P* = 2.20 × 10^−16^, *r* = 0.50). We also ran a generalized linear mixed effects model (GLMM) to take into account how some of these variables may covary (see Methods) and found that only surface had a significant effect on AMR in this model (GLMM; surface: *χ* ^2^_38,15_ = 26.14, *P* = 0.04; city: *χ* ^2^_38,19_ = 22.15, *P* = 0.28; temperature: *χ* ^2^_38,1_ = 1.72, *P* = 0.19); however, our power may have been limited to detect variation using this nested design.

### Patterns and potential factors shaping diversity

We explored both alpha diversity (diversity within sample) and beta diversity (diversity between samples) to map patterns and explore factors that may be driving community dynamics. We quantified alpha diversity for the full dataset using the Shannon Index, where a higher Shannon Index indicates greater richness with a more even representation. Overall, we found an average diversity of 1.42 (SD 0.86). We modeled factors contributing to alpha diversity including surface, latitude, longitude, and weather-nested within region using a GLMM, linear regression, and univariate ANOVAs.

**Fig. 5:**
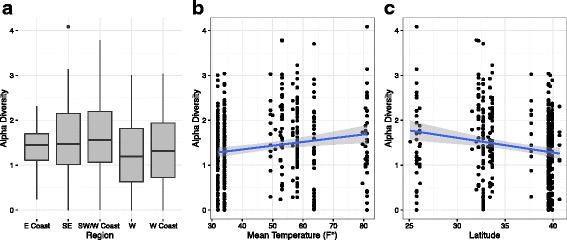
Potential factors driving variation in alpha diversity (calculated using MetaPhlAn2 results). **a** Region had a significant effect on alpha diversity (univariate ANOVA: *p* = 0.001; east removed due to small sample size). **b** Apha diversity increases with mean temperature (bivariate regression: *p* = 0.001; *r* = 0.161). **c** Alpha diversity decreases with latitude (bivariate regression: *p* = 0.0003; *r* = −0.179). Interesting because follows latitudinal diversity gradient (LDG)

Using a univariate approach, we found that region had a significant effect on diversity (ANOVA with East Coast dropped to only included regions with *N* > 10: *F*_3,396_ = 5.4, *P* = 0.001) with the Southwest/West Coast area having the highest diversity and the West having the lowest (Fig. 5). We found that surface did not have a significant effect on alpha diversity (ANOVA with AED and emergency response bag, monitor, miscellaneous, pulse O_2_ probe dropped to exclude surfaces with *N* < 10: *F*_10,378_ = 0.73, *P* = 0.70; Additional file 33: Figure S14). Although not significantly different in this model, some of the surfaces with the highest diversity include the stethoscopes and the rear bench seats. We found no correlation between diversity and longitude, but did see evidence of a positive significant correlation between species diversity and temperature (for all mean max, mean min, or mean temperatures; for mean max *t* = 3.6, df = 393, *P* = 0.0004, *r* = 0.18; Fig. 5), while finding no correlation between precipitation and diversity. Interestingly, we saw evidence that species diversity follows the “latitudinal diversity gradient (LDG)” with higher diversity found at lower latitudes (*t* = − 3.6, df = 395, *P* = 0.0003, *r* − 0.18; Fig. 5). We found that alpha diversity was significantly correlated with nosocomial pathogen hits (*t* = 12.66, df = 395, *r* = 0.54, *P* < 2.2 × 10^−16^), possibly due to increased diversity increasing one’s chance of identifying a nosocomial pathogen. We also ran a GLMM to take into account how some of these variables may covary (see Methods) and found that no variables had a significant effect on alpha diversity, however, similarly to the GLMM used to model AMR, our power may have been limited to detect variation using this nested design.

Beta diversity indicates the overall variation between sites [32]. We explored beta diversity using the Bray-Curtis distance dissimilarity and partitioned the matrix with respect to regions and surface types. We found that there were significant but weak associations between surfaces and beta diversity as well as regions and beta diversity (surface type: *r* = 0.24, *F* = 1.5, *P* = 0.0005; region: *r* = 0.15, *F* = 2.34, *P* = 0.0005; Additional file 34: Figure S15; Table 4).

**Table 4:** Results of beta diversity for MetaPhlAn2/CLARK overlap

	Sum of squares	*F*	*r*	Pr(>F)
Region	4.00	2.34	0.15	5 × 10^−4^
Region residuals	161.37			
Surface	9.84	1.55	0.24	5 × 10^−4^
Surface residuals	155.53			

PERMANOVA from the VEGAN package in R was used. Both region and surface had significant but weak effects (4000 permutations, Bray-Curtis dissimilarity matrix)

To further explore the microbial ecology of ambulances, we tested for the co-occurrence of microorganisms to gain insight into the ecology of these communities and because the presence of some species may facilitate or prevent the colonization of other species. We did an exploratory analysis on inter-organism relationships using Spearman’s rank coefficient among pairs of species (Additional file 34: Figure S15). We found that the pair that had the highest correlation (*r* = 0.81, *p* < 0.01) was *Rothia mucilaginosa* and *Streptococcus mitis,* bacteria species that inhabit the mouth. *R. mucilaginosa* has been implicated in infections associated with prosthetics. The species that were found in the most pairs and that had the highest rho (*r* > 0.5) were *P. acnes, S. mitis, and S. epidermis*; each showed up in pairs 4 times. *P. syringae* was found in 10 pairs with a weak but significant association (r between − 0.1 and 0.1, *p* < 0.05) suggesting an adaptation strategy that is more independent of other species.

## Discussion

This is the first study using metagenomics to characterize the microbiome of ambulances across a country. It was conducted on a national scale in order to explore regional factors that may be influencing the microbial ecology of ambulances. Characterization of pre-hospital as well as hospital microbial ecology is important as it may inform public health policy and healthcare practices. We found considerable variation as well as consistent patterns across samples in microbial diversity, species present, nosocomial pathogens, functional pathways, and AMR markers. We found that the majority of the microorganisms identified in ambulances were not known to be harmful, or are classified as beneficial, but we also identified species associated with nosocomial pathogens. While this is the first study to characterize the ambulance microbiome using metagenomic methods, we found concordance with other ambulance studies which used complementary methods such as culturing that found clinically relevant bacterial contamination and that more rigorous testing and cleaning of ambulances is warranted [14]. With the drastic decrease in the cost of sequencing and the ability to analyze large amounts of metagenomic data, we anticipate the growing utility and application of metagenomics in clinical environments. Furthermore, metagenomics combined with other approaches, such as RNA sequencing, culturing, or propidium monoazide (PMA) testing allow for both microbial identification and viability testing. In this study, we focus on identification of microorganisms, not viability, and provide a metagenomics baseline for ambulances, establishing a context for future studies.

### Factors shaping the microbial composition of ambulances

We found that the top 10 most abundant species are species that are either common built environment microbes (e.g., *S. maltophilia*, *P. stutzeri*), are microbes associated with the human microbiome (e.g., *P. acnes*) or those known to be associated with healthcare environments and hospital acquired infections (e.g., *E. cloacea*, *E. faecalis*) (Table 2). Many of these top 10 abundant taxa including *Pseudomonas* spp*.*, *Propionibacterium* spp*.*, *Enterobacter* spp*.*, *Staphylococcus epidermis*, *Micrococcus luteus*, and *Kocuria rhizophila* have been identified as “signature microbes” of healthcare settings, including the neonatal intensive care unit and hospital air samples [22]. Additionally, a recent large-scale metagenomics hospital study, has also found an abundance of *Staphylococcus* and *Propionibacterium* and has similarly found a preponderance of skin associated microbes on surfaces [9].

We used a machine learning approach to explore variables that contribute to the distribution of microbial populations, and we found that surfaces including rear bench seats, rear lights control panel and stethoscopes were distinguished by the abundance of three species *E. cloacea*, *M. luteus*, and *B. megaterium* (Fig. 2). Notably, these species are health- and HAI-relevant species, and may serve as a reservoir for acquiring AMR or other genetic markers. In contrast, for cities and regions, a greater number of species influenced the classification of the RF for a given class.

### Factors shaping microbial functional composition of ambulances

We used a variety of approaches for functional characterization of our samples. Using HUMAnN2, we found that the majority of pathways identified were associated with bacteria pathways for Biosynthesis, Degradation, Utilization, and Assimilation, and finally, Generation of Precursor Metabolites and Energy, which supports our taxonomic classification results. Using an RF approach, we found that several pathways were significantly more abundant on stethoscopes compared to rear bench seats, with a notable proportion of these pathways involved in aromatic compound degradation. This is interesting because aromatic compounds are common in cleaning products, such as those used in ambulances. This overabundance could indicate potential selection by the healthcare environment similarly to other studies which have shown “unnatural selection” by built environments [22]. In regard to region, we found that the west had fewer differentially abundant pathways which could be attributed to its lower levels of alpha diversity and the positive correlation we observed between the proportion of taxa present in a given sample and functional prevalence.

Using the HMP database to characterize these data further, we found an overabundance of microorganisms associated with the skin and blood. Surfaces varied significantly in the abundance of skin-associated microbes identified, with the highest levels found on surfaces that commonly come into contact with skin including rear lights/control panels and stethoscopes. High levels of skin-associated microbes have also been found on high-touch surfaces in other studies [2, 23]. An overabundance of blood-associated microbes could indicate identification of bloodborne pathogens or molecules which have been found in blood. This has been explored in studies which have identified a diversity of microbial species using cell free DNA since blood circulates through the body and collects molecules from an array of body tissue [33].

Using an a priori approach to characterize the incidence of nosocomial pathogens in the samples [28], we found widespread incidence of pathogens with the capacity to cause the majority of HAIs (78.4% of samples contained at least one nosocomial pathogen identified by the more rigorous overlap classification approach). Furthermore, over 50% of the top nosocomial pathogens on the high priority CDC list were identified in the ambulances sampled (also in overlap data). These include *S. aureus*, *K. pneumoniae*, *K. oxytoca*, *E. faecalis*, *E. faecium*, *P. aeruginosa*, *S. parasanguinis*, *A. baumannii*, and *S. maltophilia*. Many of the taxa identified commonly harbor antibiotic resistance. Our findings are in agreement with other studies which have cultured ambulances. The majority of these studies have focused on *S. aureus* identification and have found similar levels of contamination [13, 34–36].

High levels of AMR markers were identified in our samples (~ 90% of samples had hits for at least three AMR markers). A high level of AMR markers has similarly been found on surfaces in other healthcare environments, such as in hospitals [9]. These levels indicate a potential risk to patients, and EMS workers, and a pathway for AMR into hospitals. AMR are a major global health problem and are widespread, with resistance to “last line” drugs even identified in hospitals [37]. We found evidence that AMR levels may be associated with the surface and region of the country from which a sample is collected with areas with lower latitude, further east having higher levels of AMR. We also found that greater temperature, greater precipitation, and greater microbial diversity were all correlated with higher AMR levels. Finally, we found that higher levels of nosocomial pathogens were correlated with higher levels of AMR. While variation in these variables may explain variation in AMR, it is important to note that many of these variables co-vary. Taking this into account by running a GLMM, the data indicated that surface was the only variable to independently have a significant effect on AMR.

### Patterns and dynamics of microbial diversity

Due to the growing availability of molecular data for microbial species, it is now possible to test many long standing ecological theories in the realm of microbial ecology [21]. Ambulances, as well as other healthcare settings tend to be cleaned regularly creating a “disturbed” microbial ecosystem which is constantly being recolonized from patient, healthcare workers, visitors, and the environment [38, 39]. Disturbed environments have lower diversity which has been shown to be more conducive to invasive species at the macroscopic level [40] and for pathogens at the microscopic level, a property referred to as colonization resistance for microbes [41–43]. We found a positive correlation between alpha diversity and nosocomial pathogens, so in broad terms, our data do not support this theory, however this is not taking into account how combinations of microorganisms may facilitate colonization. We found evidence of co-occurrence of specific species, and further studies are warranted to explore the role of these co-occurrences (such as *R. mucilaginosa* and *S. mitis*) in the colonization resistance and microbial ecology of healthcare environments.

Exploring the relationship between microbial diversity and a number of local variables, we found that region had a significant effect on diversity with the Southwest/West Coast having the highest alpha and beta diversity. Interestingly, latitude was found to be inversely related to diversity with lower latitudes having higher diversity. The “latitudinal diversity gradient (LDG)” is a common pattern in which species diversity/richness is higher at lower latitudes. This pattern is largely accepted in the field of ecology but has rarely been studied in microorganisms. Our findings support the hypothesis that microbial species diversity may follow the LDG in agreement with at least one prior study [44].

We found that surface did not have an effect on alpha diversity, but it did have an effect on beta diversity as well as many other microbial variables (AMR, RF classification, and functional results) indicating that surface may play a role in shaping microbial communities and differentiation between microbial communities, but potentially not diversity within sample. In regard to weather-related variables, temperature (but not precipitation) was found to be positively correlated with alpha diversity. This finding is in agreement with some studies which have found that temperature is one of the driving factors determining microbial composition [45].

### Limitations and future work

Due to the high rate of false positives and the challenges associated with metagenomic analysis [46], we took a conservative approach to increase our confidence in species identification and report taxa classified by multiple published tools including commonly used MetaPhlan2 and CLARK as well as their overlap. By generating synthetic data to test our workflow, we found that working with the overlap data was a trade-off that increased our precision but decreased our sensitivity.

An additional challenge in characterizing pathogens using metagenomic data is that genus and species level identification may group pathogenic and non-pathogenic species and/or strains, but resolutions to the strain level are still computationally challenging. We addressed this by using a multi-tool approach, comparing results and by examining coverage across genomes for species of interest. Future work includes metagenomic assembly and exploration of pathogenicity using targeted PCR-based assays.

A limitation of this study was that we used the approach of swabbing and DNA sequencing but did not use the complementary approach of culturing so our results indicate that DNA collected and sequenced had best matches to the databases used but species reported might have been misidentified (due to high sequence similarity among some species or database limitations), or not viable or presenting an infection risk. We worked to ameliorate misclassification by using multiple classification tools and reporting overlapping results. Future ambulance work would benefit from culturing, complementary sequencing approaches, collection of additional metadata, and sampling both ambulances and healthcare environments to better characterize the role ambulances play as a vector for HAIs and AMRs.

## Conclusions

To the best of our knowledge, this is the only metagenomic study of ambulances to date  and our findings indicate that this approach is a useful way to characterize microbial communities in pre-hospital environments. Our methods demonstrate a multipronged approach of analysis, using complementary approaches, including using multiple classifiers, taking the overlap of these classification tools, and using a classification and functional approach, which provides for a more rigorous and reliable analysis.

Overall we found at least six factors influence the microbial ecology of ambulances including ambulance surfaces, geographical-related factors (including region, longitude, and latitude), and weather-related factors (including temperature and precipitation). We also found evidence of microbes associated with hospital-acquired infections and AMR markers in ambulances, presenting a possible source for HAIs and AMR. Our findings indicate additional, or targeted, testing and cleaning may be warranted in ambulances. These data represent the first baseline metagenomic characterization of ambulances, which will be a useful guide for future studies and more adaptive surveillance.

## Methods

### Sample collection

From 2013 to 2014, we collected 1407 samples from ambulances across the USA and sequenced a subset of 398 of these samples to include a breadth of locations from 137 ambulances in 19 cities (de-identified to protect ambulance privacy) in 6 states (Fig. 1). All samples were collected using Copan Liquid Amies Elution Swabs 481C, which are nylon-flocked swabs that we have found optimize the amount of sample collected from the environment [2]. These swabs include 1 mL transport medium, with a pH-neutral buffer that preserves RNA and DNA. The surfaces swabbed included computers, steering wheels, keyboards, medical equipment (stethoscopes, pulse ox probes, blood pressure cuffs and bulbs, control panels, AEDs, and monitors), stretchers, handles, rails, and cabinets for each ambulance. Surfaces were chosen to include high-touch surfaces and medical equipment that came in close contact with patients and healthcare workers and could act as a source or sink for microbiota. All surfaces swabbed were inside ambulances, except for handles and rails which included surfaces external to ambulances and were included because they were frequently touched by healthcare workers. To ensure the highest yield, swabs were dipped in the elution buffer before collection and surfaces were swabbed for 3 min. Samples were stored at − 80 °C until they were processed.

### Laboratory sample processing

To process these samples, we followed established protocols for sequencing and analysis [2], including MoBio Powersoil DNA extraction, Qubit 2.0 fluorometer quantification, paired end (125 bp × 125 bp) sequencing on the Illumina HiSeq2500 machine with an insert size of 600.9 bp (SE ± 88.7), generating 14.3 M (SE ± 0.4 M) reads per sample. On average 12.6 M (SE ± 0.3 M) reads passed an initial filter and were used in all further analyses. These data were analyzed using a combination of existing bioinformatics tools and custom scripts using a custom workflow (Fig. 1).

### Negative and positive controls

Negative control samples were collected in each ambulance by opening the swab, exposing it to the air for 1 s, and placing it in the media. These swabs were handled and stored in the same manner as other samples collected. DNA was extracted from these samples and quantified following the same protocol as other samples. DNA extracted from control samples was verified to be negligible (< 0.05 ng/μL compared to 138.89 ng/μL for non-control samples).

In this study, we present and further analyze results from multiple commonly used published classification tools MetaPhlAn2 and CLARK. In addition to presenting results from these individual classification tools, in our overlap results, we also present a set of higher confidence results based on classification by both MetaPhlAn2 and CLARK. Our goal in generating and analyzing overlap data was to maximize accuracy even at the expense of sacrificing some sensitivity. Positive control datasets were generated synthetically to estimate the accuracy and precision of using MetaPhlAn2, CLARK, and the two tools combined (their overlap). Synthetic datasets were created using the simulation tool ART, which allows for generation of synthetic sequence reads including platform-specific error simulation [47]. Three datasets (DS1, DS2, and DS3) were created based on experimental ambulance data to include the top seven species of interest which cause hospital acquired infections, as well as an additional 10 species which were found to have the highest abundance in ambulances. The other synthetic datasets used (SimBA-525, Buc12, CParMed48, Gut20, Hou21, Hou31, and Soi50) are published unambiguous datasets [48]. The overlapping classifications (those made by both MetaPhlAN2 and CLARK) had the greatest precision at the cost of a slightly lower sensitivity (Additional file 1: Table S1). Further analyses were conducted on either MetaPhlAn2, CLARK, or overlap data as deemed appropriate as indicated in Additional file 2: Table S2.

### Sequence analysis

#### Processing and classification

Sequences were trimmed for quality using FASTX-Toolkit (http://hannonlab.cshl.edu/fastx_toolkit/) based on a Q20 cutoff and adaptors were trimmed using CutAdapt [49]. We have compared identification tools by analyzing metagenomic samples of known composition (titrated mixtures of bacteria and synthetic DNAs) and found a multi-tool approach to be most reliable [27, McIntyre et al. in press]. Therefore, processed reads were analyzed using MetaPhlAn v2.0 and CLARK to identify and determine relative abundance of species. These tools use probabilistic matching approaches, comparing DNA to curated databases of species-specific sequence fragments. In previous work, we found MetaPhlAn2 has the highest positive predictive value (PPV), while CLARK scores lower on PPV but higher on sensitivity. Here we report the classification results for each classification tool (Additional file 3: Figure S1, Additional file 4: Table S3, Additional file 5: Table S4) as well as the species identified by both tools, the overlap (Additional file 6: Figure S2, Additional file 7: Table S5). Further analyses were conducted on this MetaPhlAn2/CLARK overlapping dataset to increase our confidence in species classified.

### Functional analysis

#### HUMAnN2

Functional genomic profiles of the dataset were generated using HUMAnN2 version 0.5.0. HUMAnN2 utilizes the MetaCyc, UniPathway, and KEGG databases as well as the UniRef gene family catalog to characterize the microbial pathways present in samples. HUMAnN2 was run under default parameters (see Additional file 35 for code and scripts). HUMAnN2 generates three outputs: (1) gene families based on UniRef proteins and their abundances reported in reads per kilobase, (2) MetaCyc pathways and their coverage, (3) MetaCyc pathways and their abundances reported in reads per kilobase. We focused our follow-up analysis and interpretation based on the third output as we wanted to study the functional pathways present in our samples and wanted to perform analyses based on abundance and not coverage, which only tells you if the pathway was found to be present or absent.

For follow-up analysis, we manually curated and annotated our results based on metadata on the MetaCyc database (http://metacyc.org/). For many of these pathways, further information on their taxonomic range, superclass, category, and molecules involved are posted on the MetaCyc database. Note that not all pathways had information posted on the MetaCyc database for further analysis.

#### Human Microbiome Project annotation

We annotated our MetaPhlAn2/CLARK overlapping dataset using the healthy Human Microbiome Project (HMP) dataset (downloadable from http://www.hmpdacc.org/HMRGD/healthy/#data, downloaded August 11 2016), which includes additional sites including blood and heart, to identify the regions of the human body highly associated with species we identified. To determine whether the ambulance proportions identified were due to what is available in the HMP database, we tested the proportions using a chi-square test and took the log_2_ of the observed versus expected counts.

#### Nosocomial pathogens and AMR

As a preliminary exploration of nosocomial pathogens and AMR, we conducted an a priori search of our overlap data for pathogens which are identified as causing the majority of nosocomial infections [28]. For samples with hits for nosocomial pathogens of interest such as *S. aureus*, we conducted further analyses including alignments to reference genomes using BWA (v7.10) [50]. For *S. aureus*, the reference genome we used was USA300 strain (USA300_FPR3757 GCA_000013465.1_ASM1346v1), a methicillin-resistant *S. aureus* (MRSA) strain which is documented to cause both community and hospital acquired infection. We generated multi pileup files using Samtools (v1.19), and analysis of coverage over virulence, phylogenetic, and AMR markers compiled from RefSeq databases using Bedtools (v2.18) [51], and then visualized in the Integrative Genome Viewer (IGV) [52] and a custom R script (Additional file 35).

To explore AMR, a CLARK database of AMR markers produced from the Comprehensive Antibiotic Resistance Database (CARD) was constructed using a combination of custom scripts (see Additional file 35), and the CLARK built-in custom database function. The CARD database was selected because it is the most current, manually curated AMR database. It includes sequence data for all AMR drug classes and resistance mechanisms (e.g., mutation-based, or acquired resistance). An exact k-mer match to the database sequence was required to report an AMR marker. The final abundance estimation of antibiotic markers was generated using a custom script with a CLARK positive identification hit threshold of 150. Further analysis and characterization of pathogens are ongoing.

#### Alpha and beta diversity

To explore diversity, we calculated both alpha and beta diversity and explored factors contributing to the diversity observed. Shannon diversity index was calculated from the MetaPhlan2 data using R package Vegan with default parameters [53]. Metaphlan2 results were used instead of overlap data to calculate alpha diversity because Shannon index relies on both the species diversity and evenness across species, so subsetting is not appropriate. Bray-Curtis dissimilarity was calculated to estimate beta diversity using the overlap data and the Vegan R package.

Since weather, including temperature and humidity have shown to play a prominent role in microbial diversity [45], weather data was downloaded and used in modeling alpha and beta diversity. Weather data including average maximum temperature, average minimum temperature, average temperature, and total precipitation for the month previous to and the month of the collection were downloaded for the weather station closest to the collection location from National Oceanic and Atmospheric Administration (NOAA; http://www.ncdc.noaa.gov/cdo-web/).

#### Modeling and statistical analysis

A variety of machine learning classifiers including random forest (RF), regularized random forest, support vector machine (linear, rbf, and polynomial kernels), gradient boosting, partial least squares, k nearest neighbors, and decision trees (C5.0) were explored to maximize and assess the ability of microbial and functional composition at predicting sample surface, city, region, and front versus rear ambulance surfaces. Surfaces, city, regions, and taxa with fewer than 20, 10, 10, and 3 samples were excluded to improve class balance and ensure the presence of low prevalence features across cross validation splits. This resulted in the following class labels: Computer (95), Front Handles (32), Rear Bench Seats (31), Rear Cabinets Counters (32), Rear Lights Control Panel (31), Steering Wheel Driver Controls (27), Stethoscope (45), Stretcher (29), and Suction O2 (32) for sample surface; S005 (49), S006 (49), S010 (13), S019 (24), S003 (113), S002 (36), S004 (13), S007 (30), S008 (7), S016 (22), and S013 (11) for deidentified city; Southeast (35), Southwest Coast (130), West (98), and West Coast (126) for region; and Front (153) and Rear (243) ambulance. Features were centered and scaled. For training, 20% of the data were randomly sampled and set aside for testing to assess generalizability. The remaining 80% were used as a training-validation test for repeated (10×) 10-fold cross validation. Using cross validation on the training-validation data, we performed parameter sweeps on an array of classifiers. To overcome issues arising from class imbalance, down-sampling was performed for all runs except when classifying region, which instead underwent up-sampling. Models were evaluated based on mean ROC score. To then assess prediction performance as a function of dataset, we performed a parameter sweep using a RF classifier for microbial composition data (MetaPhlAn2 and MetaPhlAn2/CLARK overlap data) and functional data (HUMAnN).

The generalization error of the resulting best-fit RF (mtry = 8, ntree = 128) was evaluated using the overlap test set. Importance rankings were assessed to characterize which taxa had the greatest impact on classification. Classification performance and ordination of the RF proximity scores were used to identify classes with strong classification performance. Each combination from the identified classes then underwent a DESeq2 differential abundance analysis with FDR correction (alpha = .01) using the HUMAnN2 functional dataset to identify significant differences in functional content in surfaces and regions with predictive microbial configurations [54]. These analyses were conducted in R (V3.2.3) using Vegan, Phyloseq, and Caret packages.

To determine how total AMR count per sample and alpha diversity was influenced by variables including surface type, city, latitude, temperature and precipitation, 2 separate generalized linear mixed effects models (GLMMs) were constructed, one to model AMRs and one to model alpha diversity. We used mixed models because our experimental design was hierarchically nested by region. The model was run in R using the lme4 package [55] with region as a random effect, all other variables fixed, a Gaussian link function and maximum likelihood for model estimation (see Additional file 35: for code). Total AMR count per sample were high (mean 499) so were treated as continuous data, and were log transformed before analysis to meet model assumptions. Models were fit with all variables and then variables were removed one at time and models compared using ANOVA to estimate test statistics. After fitting full nested models, we explored the correlation between specific variables of interest further by conducting bivariate linear regressions and univariate ANOVAs. In interpreting bivariate linear regression results, many of these variables may potentially explain variance in AMR counts, but some of these are highly correlated. GLMM is a useful lens to interpret these data but may be limited in power due to our experimental design. We also analyzed AMR counts in each sample for each marker and looked for associations with variables measured using DESeq2, anosim, and permanova and got very few significant or zero markers most likely due to a substantial degree of sparsity (95% of the values were zero).

Non-parametric permutational MANOVA was used to determine if either region or surface area type had an effect on AMRs or beta diversity patterns (using the adonis function in R package Vegan). We regressed region and surface area type separately against either the AMR count (for one model) or the Bray-Curtis dissimilarity matrix (for another model) and permuted the data 4000 times. Principal coordinates analysis (PCoA) plots were created using the matrices after standardizing the values of the rows between 0 and 1. To balance sample groups, we randomly sampled (without replacement) from over-represented groups and excluded regions or surface types that had fewer than 25 observations per group. We created the PCoA plots using the ade4 package in R, which uses a dissimilarity matrix as an input and performs the eigen-decomposition. The first two components of the resulting matrix were then plotted.

Correlation analysis was done using the Hmisc package in R [56]. We included species from the overlap data that had a relative abundance total of at least 10. We used the Spearman’s rank correlation coefficient as it is robust to outliers and skewness. We plotted the coefficients after filtering the output to include only significant (*p* < 0.05) pairs of species.

## Additional files

Additional file 1: **Table S1.** Precision and sensitivity of our computational pipeline were tested using synthetic datasets. The overlapping classifications (those made by both MetaPhlAN2 and CLARK) had the greatest precision but lower sensitivity. (DOCX 111 kb)

Additional file 2: **Table S2.** Delineation of which classification results were used for each analysis and the rationale for these choices. (DOCX 86 kb)

Additional file 3: **Figure S1.** Krona plot of classification results for all data using (A) MetaPhlan2, and (B) CLARK classification tools. (DOCX 2427 kb)

Additional file 4: **Table S3.** Total MetaPhlan2 results. (TSV 1562 kb)

Additional file 5: **Table S4.** Total CLARK results. (TXT 1568 kb)

Additional file 6: **Figure S2.** Venn diagram of CLARK and MetaPhlan2 total count of overlapping classifications at the (A) species, and (B) genus levels. (DOCX 2708 kb)

Additional file 7: **Table S5.** Total relative abundance, average relative abundance and standard deviation of the relative abundance for all species identified by both MetaPhlan2 and CLARK. (XLSX 380 kb)

Additional file 8: **Figure S3.** Heatmaps showing average relative abundances of all species identified by both MetaPhlan2 and CLARK (relative abundance from MetaPhlan2) across (A) cities, (B), regions, and (C) surfaces. Data shown is square root transformed. (DOCX 2145 kb)

Additional file 9: **Table S6.** Manually curated annotations of species in overlap data. (XLSX 61 kb)

Additional file 10: **Figure S16.** Boxplots of classifier performance over model specific parameter sweeps during training (80/20 split) on overlap data for surface class. Classes underwent down sampling and were optimized in terms of mean ROC score. Shown are kappa and balanced accuracy, averaged over classes. rf, random forest; gbm, stochastic gradient boosting; rrf, regularized random forest; c50, c5.0 decision tree, pls, partial least squares; en, elastic net; knn, k-nearest neighbors; svm linear, support vector machine with linear kernel; rbf svm, support vector machine with rbf kernel. (DOCX 92 kb)

Additional file 11: **Figure S17.** Boxplots of classifier performance over model specific parameter sweeps during training (80/20 split) on MetaPhlAn2 data for surface class. Classes underwent down sampling and were optimized in terms of mean ROC score. Shown are kappa and balanced accuracy, averaged over classes. rf, random forest; gbm, stochastic gradient boosting; rrf, regularized random forest; c50, c5.0 decision tree, pls, partial least squares; en, elastic net; knn, k-nearest neighbors; svm linear, support vector machine with linear kernel; rbf svm, support vector machine with rbf kernel. (DOCX 96 kb)

Additional file 12: **Figure S18.** Boxplots of classifier performance over model specific parameter sweeps during training (80/20 split) on overlap data for region class. Classes underwent up sampling and were optimized in terms of mean ROC score. Shown are kappa and balanced accuracy, averaged over classes. rf, random forest; gbm, stochastic gradient boosting; rrf, regularized random forest; c50, c5.0 decision tree, pls, partial least squares; en, elastic net; knn, k-nearest neighbors; svm linear, support vector machine with linear kernel; rbf svm, support vector machine with rbf kernel. (DOCX 101 kb)

Additional file 13: **Figure S19.** Boxplots of classifier performance over model specific parameter sweeps during training (80/20 split) on MetaPhlAn2 data for region class. Classes underwent up sampling and were optimized in terms of mean ROC score. Shown are kappa and balanced accuracy, averaged over classes. rf, random forest; gbm, stochastic gradient boosting; rrf, regularized random forest; c50, c5.0 decision tree, pls, partial least squares; en, elastic net; knn, k-nearest neighbors; svm linear, support vector machine with linear kernel; rbf svm, support vector machine with rbf kernel. (DOCX 141 kb)

Additional file 14: **Figure S20.** Boxplots of classifier performance over model specific parameter sweeps during training (80/20 split) on overlap data for city class. Classes underwent up sampling and were optimized in terms of mean ROC score. Shown are kappa and balanced accuracy, averaged over classes. rf, random forest; gbm, stochastic gradient boosting; rrf, regularized random forest; c50, c5.0 decision tree, pls, partial least squares; en, elastic net; knn, k-nearest neighbors; svm linear, support vector machine with linear kernel; rbf svm, support vector machine with rbf kernel. (DOCX 97 kb)

Additional file 15: **Figure S21.** Boxplots of classifier performance over model specific parameter sweeps during training (80/20 split) on MetaPhlan data for city class. Classes underwent up sampling and were optimized in terms of mean ROC score. Shown are kappa and balanced accuracy, averaged over classes. rf, random forest; gbm, stochastic gradient boosting; rrf, regularized random forest; c50, c5.0 decision tree, pls, partial least squares; en, elastic net; knn, k-nearest neighbors; svm linear, support vector machine with linear kernel; rbf svm, support vector machine with rbf kernel. (DOCX 134 kb)

Additional file 16: **Figure S22.** Boxplots of dataset performance for random forest training (80/20 split) for surface class. Classes underwent down sampling and were optimized in terms of mean ROC score. Shown are kappa and balanced accuracy, averaged over classes. (DOCX 107 kb)

Additional file 17: **Figure S23.** Boxplots of dataset performance for random forest training (80/20 split) for region class. Classes underwent down sampling and were optimized in terms of mean ROC score. Shown are kappa and balanced accuracy, averaged over classes. (DOCX 115 kb)

Additional file 18: **Figure S24.** Boxplots of dataset performance for random forest training (80/20 split) for city class. Classes underwent down sampling and were optimized in terms of mean ROC score. Shown are kappa and balanced accuracy, averaged over classes. (DOCX 113 kb)

Additional file 19: **Figure S25.** ROC curve of random forest test set performance (80/20 split) on front-rear surface class. Classes underwent down sampling and were optimized in terms of ROC score. (DOCX 51 kb)

Additional file 20: **Figure S4.** Normalized feature important for overlap data during random forest training (80/20 split) for surface class. Classes underwent down sampling and were optimized in terms of mean ROC score. Shown are kappa and balanced accuracy, averaged over classes. (DOCX 566 kb)

Additional file 21: **Figure S5.** Normalized feature important for overlap data during random forest training (80/20 split) for region class. Classes underwent up sampling and were optimized in terms of mean ROC score. Shown are kappa and balanced accuracy, averaged over classes. (DOCX 308 kb)

Additional file 22: **Figure S6.** Normalized feature important for overlap data during random forest training (80/20 split) for city class. Classes underwent up sampling and were optimized in terms of mean ROC score. Shown are kappa and balanced accuracy, averaged over classes. (DOCX 553 kb)

Additional file 23: **Figure S7.** Overlap binned abundances (RPK) over samples for the top 3 ranking species (columns) in terms of feature importance from random forest classification training (80/20 split, 128 trees). Red bars correspond to the top ranking feature for that deidentified city (row). (DOCX 452 kb)

Additional file 24: **Table S7.** Total HUMAnN2 results and MetaCyc annotations. (XLSX 2419 kb)

Additional file 25: **Figure S8.** Volcano plot of the *p*-value versus log_2_-fold change (LFC) of HUMAnN2 pathway abundances resulting from a DESeq2 differential abundance analysis for surface class with FDR correction (Benjamini-Hochberg correction, *α* = 0.01). Class combinations were selected based on overlap data classification performance. Points vary in color based on pathway superclass and size based on the proportion of genes in that class with p < α. Genes in the 95th percentile of absolute LFC are labeled. (PDF 273 kb)

Additional file 26: **Figure S9.** Volcano plot of the *p* value versus log_2_-fold change (LFC) of HUMAnN2 pathway abundances resulting from a DESeq2 differential abundance analysis for region class with FDR correction (Benjamini-Hochberg correction, *α* = 0.01). Class combinations were selected based on overlap data classification performance. Points vary in color based on pathway superclass and size based on the proportion of genes in that class with p < α. Genes in the 95th percentile of absolute LFC are labeled. (PDF 381 kb)

Additional file 27: **Figure S10.** Shannon alpha diversity for overlap data. (DOCX 25 kb)

Additional file 28: **Figure S11.** Scatter plot of HUMAnN2 gene prevalence (proportion of pathways found in sample i) versus overlap species prevalence (proportion of species found in sample i). Each point represents a sample, with colors representing the sample region. (DOCX 120 kb)

Additional file 29: **Figure S12.** Volcano plot of the p-value versus log_2_-fold change (LFC) of HUMAnN2 pathway abundances resulting from a DESeq2 differential abundance analysis for city class with FDR correction (Benjamini-Hochberg correction, *α* = 0.01). Class combinations were selected based on overlap data classification performance. Points vary in color based on pathway superclass and size based on the proportion of genes in that class with p < α. Genes in the 95th percentile of absolute LFC are labeled. (PDF 338 kb)

Additional file 30: **Table S8.** Sequence coverage over *S. aureus* antibiotic and virulence factors *femA*, *femB*, and *mecA* for all samples with *S. aureus* hits. (XLSX 10 kb)

Additional file 31: **Figure S13.** Evidence of non-MRSA *S. aureus* found in ambulance samples. (A) Visualization of sequence coverage across genome of sample with highest relative abundance of *S. aureus* (AW0974) shows lack of coverage over *mecA* and high consistent coverage over *femA* and *femB* (gene locations marked on X-axis) and most of the genome (average 20× coverage ranging from 0 to 174). (B-C) Sequence coverage over *mecA* for the two *S. aureus* positive samples with the highest level of *mecA* coverage shows lack of evidence for MRSA. (DOCX 3569 kb)

Additional file 32: **Table S9.** Hits for AMR markers in CARD database for all samples. (TXT 1834 kb)

Additional file 33: **Figure S14.** Boxplot of alpha diversity across surfaces (Shannon index calculated based on Metaphlan2 results). Surface did not have a significant effect on alpha diversity (univariate ANOVA with surfaces *N* < 10 dropped: *P* = 0.70). (DOCX 122 kb)

Additional file 34: **Figure S15.** Potential factors driving variation in beta diversity (calculated using MetaPhlAn2/CLARK overlap). Beta diversity was calculated with relative abundances, using the VEGAN package in R. Data were standardized [0,1] and balanced through random sampling (for each region, *n* = 36; for each surface, *n* = 25). (A) By surface, (B) by region, (C) Correlation plot using Spearman’s rank coefficient, including only species that had total relative abundance > 10, *P* < 0.05. (DOCX 552 kb)

Additional file 35: Supplemental Methods. (DOCX 32 kb)

Additional file 36: **Table S10.** Ambulance study metadata. (TXT 35 kb)

## Abbreviations

AMR: Antimicrobial resistance
GLMM: Generalized linear mixed effects models
HAIs: Hospital-acquired infections
HMP: Human microbiome project
LFC: log_2_ fold
ML: Machine learning
NGS: Next-generation sequencing
NOAA: National Oceanic and Atmospheric Administration
PCoA: Principal coordinates analysis
RF: Random forest
ROC curve: Receiver operating characteristic curve
