# Supplementary material for: Metagenomic characterization of ambulances across the USA
Source: Microbiome. 2017 Sep 22;5:125. doi: 10.1186/s40168-017-0339-6 (PMC5610413; doi:10.1186/s40168-017-0339-6)
Supplement: Supplementary file 1 — Table S1. Precision and sensitivity of our computational pipeline were tested using synthetic datasets. The overlapping classifications (those made by both MetaPhlAN2 and CLARK) had the greatest precision but lower sensitivity. (DOCX 111 kb) [file 40168_2017_339_MOESM1_ESM.docx]

**Table S1**. Overlapping classifications (made by both MetaPhlAN2 and CLARK) had the greatest precision but lower sensitivity.

| **Datasets** | **CLARK (A)** | **CLARK (B)** |  | **MetaPhlan2 (A)** | **MetaPhlan2 (B)** |  | **Overlap (CLARK, MetaPhlan2) (A)** | **Overlap (CLARK, MetaPhlan2) (B)** |
| --- | --- | --- | --- | --- | --- | --- | --- | --- |
| *DS1* | 19.0% | **100.0%** |  | **92.3%** | **100.0%** |  | **92.3%** | **100.0%** |
| *DS2* | 16.7% | **100.0%** |  | **90.9%** | **100.0%** |  | **90.9%** | **100.0%** |
| *DS3* | 8.2% | **100.0%** |  | **100.0%** | **100.0%** |  | **100.0%** | **100.0%** |
| *SimBA-525* | 77.9% | **99.8%** |  | **100.0%** | 76.1% |  | **100.0%** | 75.9% |
| *Buc12* | 11.8% | **83.3%** |  | **100.0%** | **83.3%** |  | **100.0%** | 66.7% |
| *CParMed48* | 28.4% | **100.0%** |  | **100.0%** | 72.9% |  | **100.0%** | 72.9% |
| *Gut20* | 10.6% | **95.0%** |  | **100.0%** | 65.0% |  | **100.0%** | 60.0% |
| *Hou21* | 42.9% | **100.0%** |  | **100.0%** | 80.0% |  | **100.0%** | 80.0% |
| *Hou31* | 19.2% | **100.0%** |  | **100.0%** | 65.0% |  | **100.0%** | 65.0% |
| *Soi50* | 21.9% | **100.0%** |  | **100.0%** | 86.0% |  | **100.0%** | 86.0% |

Precision and sensitivity of our computational pipeline were tested using synthetic datasets. The overlapping classifications (those made by both MetaPhlAN2 and CLARK) had the greatest precision but lower sensitivity. (A) Precision which is the ratio between the number of species correctly predicted and the number of species predicted. (B) Sensitivity which is the ratio between the number of species correctly predicted and the number of expected species in the dataset. The highest value for A or B are bolded for each dataset. Dataset: DS1, DS2 and DS3 are three synthetic datasets created based on microbial abundance results from ambulance samples (Ounit et al submitted 2017). The other datasets are a set of unambiguous published datasets (Ounit and Lonardi 2016).
