# Supplementary material for: Metagenomic characterization of ambulances across the USA
Source: Microbiome. 2017 Sep 22;5:125. doi: 10.1186/s40168-017-0339-6 (PMC5610413; doi:10.1186/s40168-017-0339-6)
Supplement: Supplementary file 2 — Table S2. Delineation of which classification results were used for each analysis and the rationale for these choices. (DOCX 86 kb) [file 40168_2017_339_MOESM2_ESM.docx]

**Table S2**. Delineation of which classification results were used for each analysis and the rationale for these choices.

| **Analysis** | **MetaPhlAn2** | **CLARK** | **Overlap** | **Rationale** |
| --- | --- | --- | --- | --- |
| Classification | X | X | NA | NA |
| HUMAnN2 | X |  |  | To not constrain - cast wide net. MetaPhlAn2 input is used because that is how the tool is designed. |
| HMP |  |  | X | Hand annotated/curated and interested in high confidence species |
| A priori HAI annotations |  |  | X | Hand annotated/curated and interested in high confidence species |
| CARD AMR |  | X |  | Used CLARK's feature which allows for custom database creation and the CARD database - used all FASTQ files |
| Alpha diversity | X |  |  | Shannon index relies on both the species diversity and evenness across species, so subsetting is not appropriate. Chose MetaPhlAn2 results over CLARK because more conservative (lower sensitivity but higher precision) |
| Beta diversity |  |  | X | Interested in modelling less noise and high confidence species identifications |
| Random Forest | X | X | X | To assess prediction performance as a function of dataset we ran results from individual classifiers as well as overlap data. Overlap data is reported in results because all performed similarly. |
| Generalized linear models | X | X – CARD AMR output |  | Ran as continuous data so better to work with the larger dataset from the individual classifiers |
