# Supplementary figures and images for: Metagenomic characterization of ambulances across the USA
Source: Microbiome. 2017 Sep 22;5:125. doi: 10.1186/s40168-017-0339-6 (PMC5610413; doi:10.1186/s40168-017-0339-6)

Figure S1: Summary krona plots for MetaPhlAn2 (A) and CLARK (B) results.


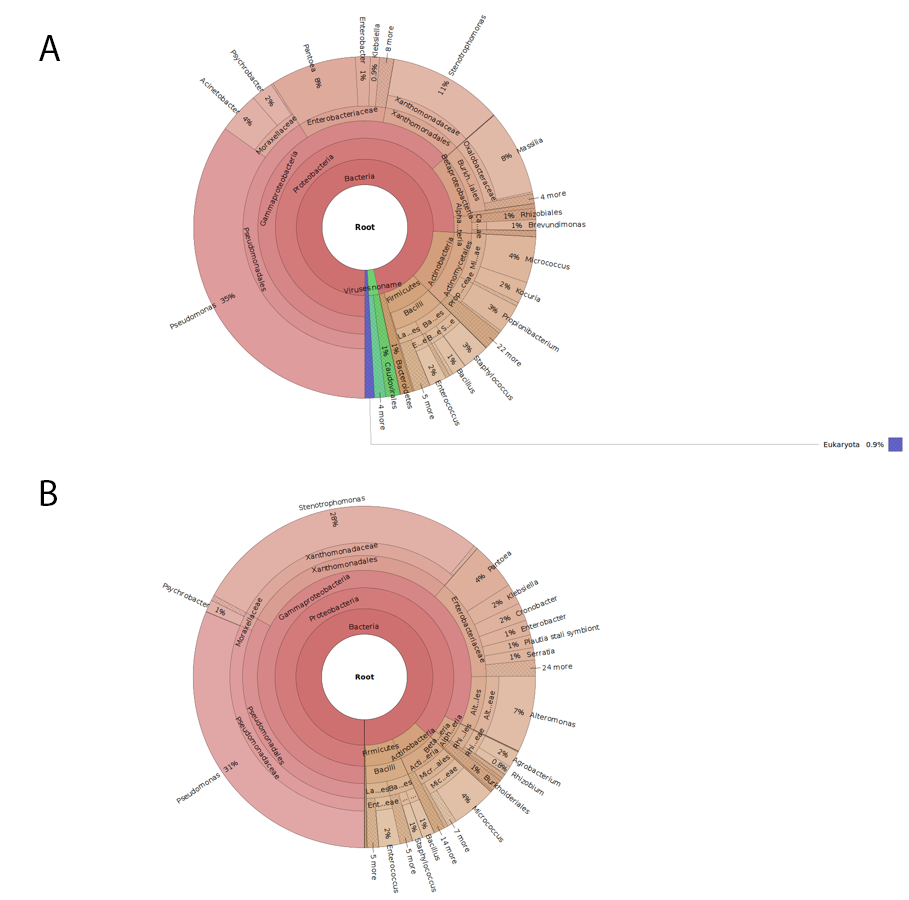

Supplement: Supplementary file 3 — Figure S1. Krona plot of classification results for all data using (A) MetaPhlan2, and (B) CLARK classification tools. (DOCX 2427 kb) [file 40168_2017_339_MOESM3_ESM.docx]

Figure S2: Venn diagram of overlap of classification tools MetaPhlAn2 and CLARK.


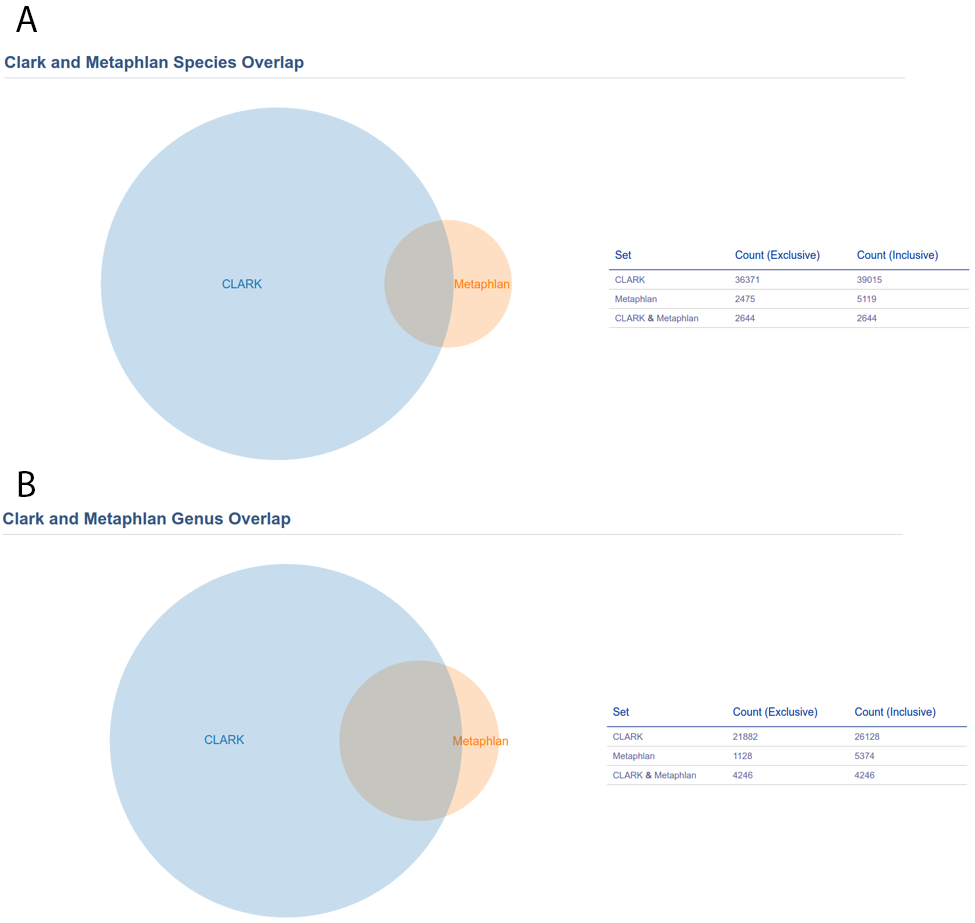

Supplement: Supplementary file 6 — Figure S2. Venn diagram of CLARK and MetaPhlan2 total count of overlapping classifications at the (A) species, and (B) genus levels. (DOCX 2708 kb) [file 40168_2017_339_MOESM6_ESM.docx]

Figure S3: Heatmaps mean abundance of overlap species across cities (A), regions (B), and surfaces (C).


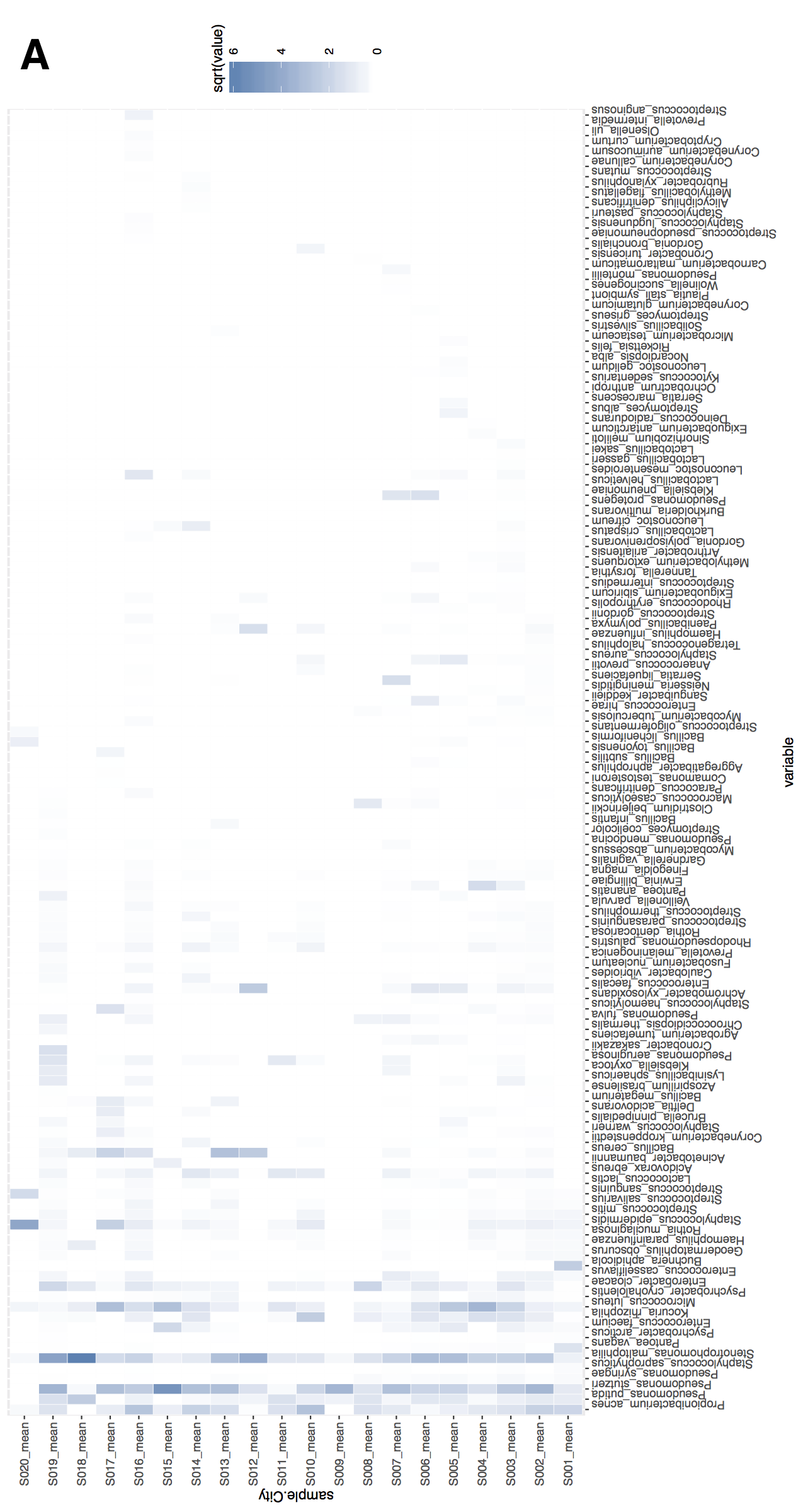


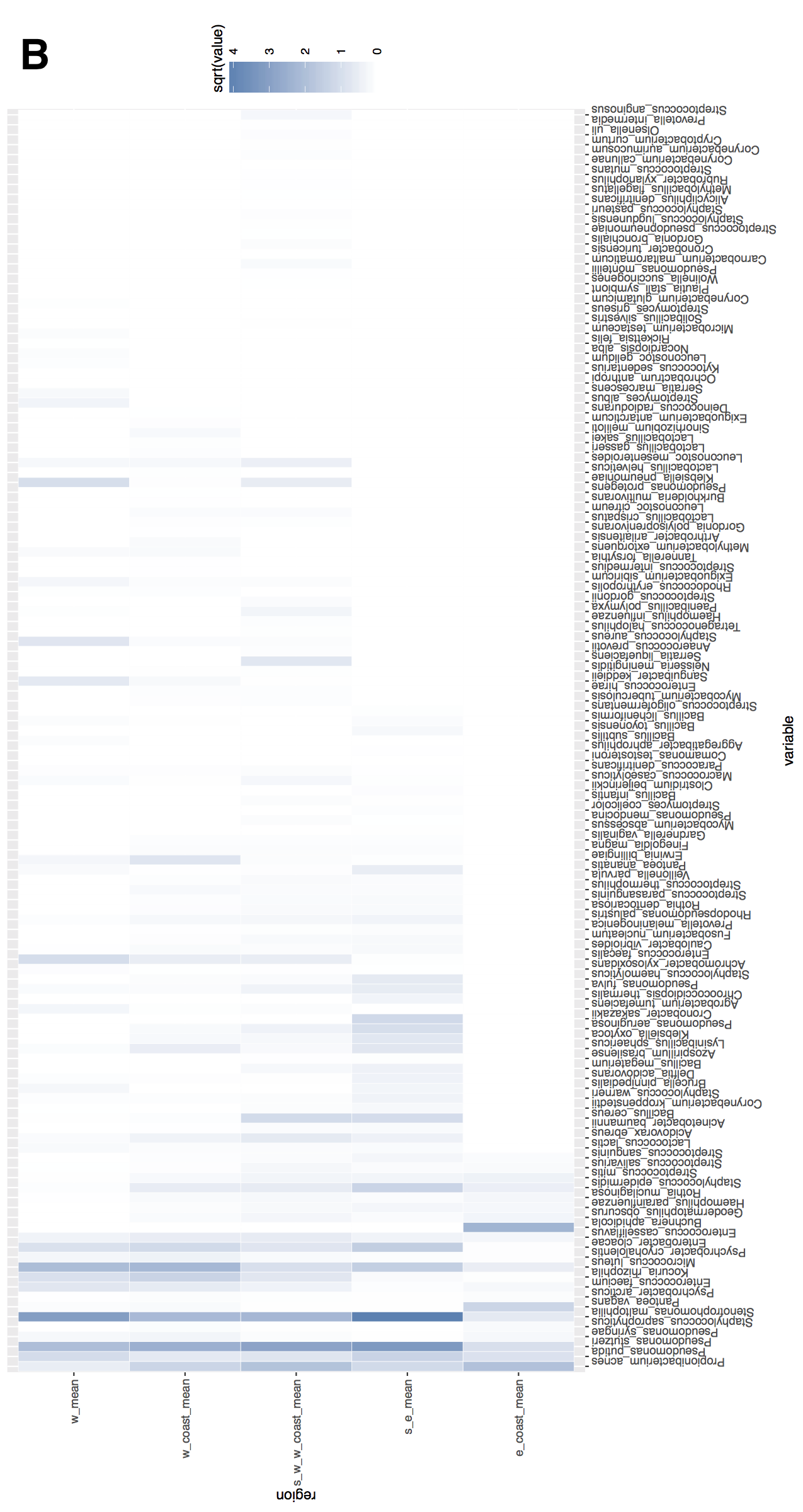


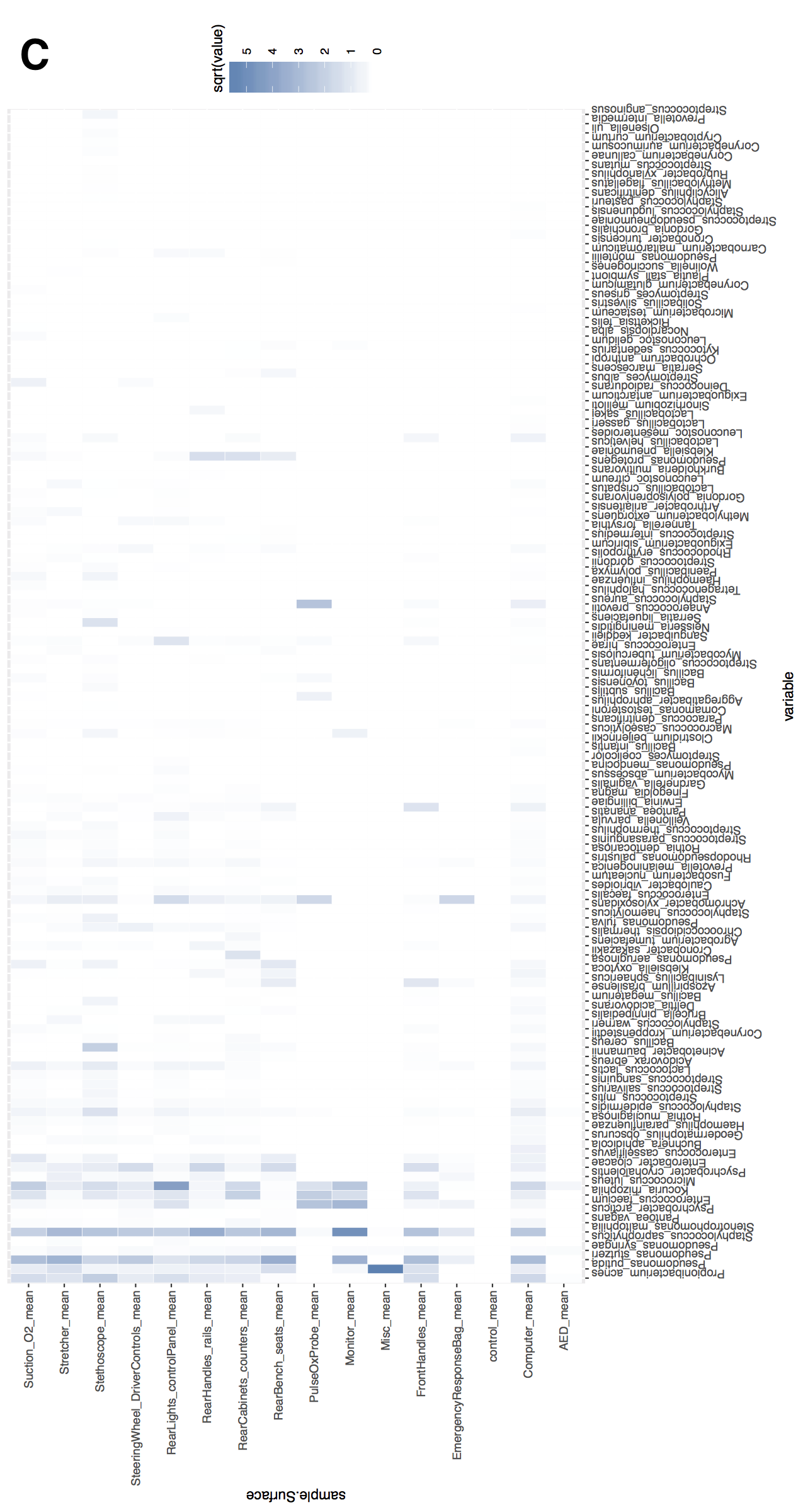

Supplement: Supplementary file 8 — Figure S3. Heatmaps showing average relative abundances of all species identified by both MetaPhlan2 and CLARK (relative abundance from MetaPhlan2) across (A) cities, (B), regions, and (C) surfaces. Data shown is square root transformed. (DOCX 2145 kb) [file 40168_2017_339_MOESM8_ESM.docx]

Figure S16: classifier results for overlap, surface


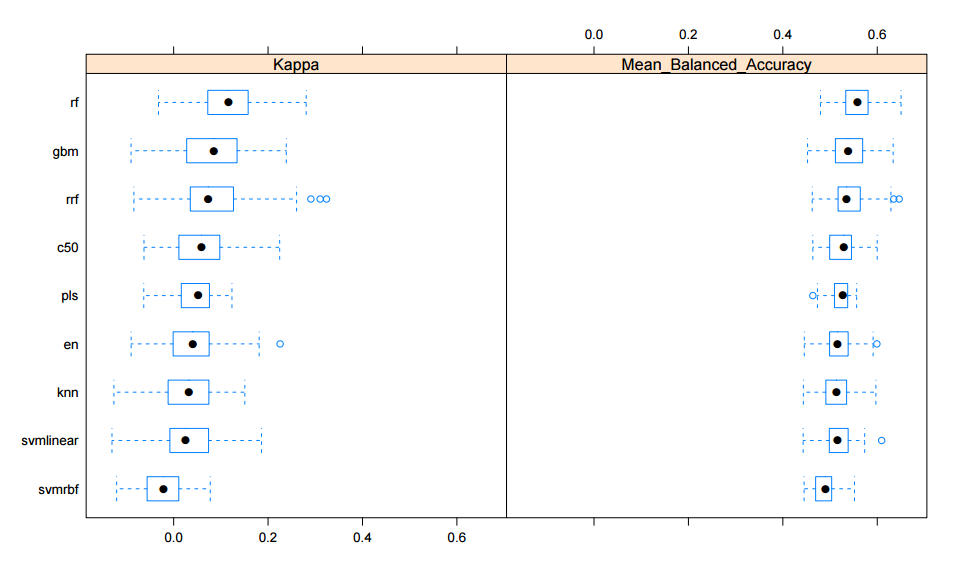

Supplement: Supplementary file 10 — Figure S16. Boxplots of classifier performance over model specific parameter sweeps during training (80/20 split) on overlap data for surface class. Classes underwent down sampling and were optimized in terms of mean ROC score. Shown are kappa and balanced accuracy, averaged over classes. rf, random forest; gbm, stochastic gradient boosting; rrf, regularized random forest; c50, c5.0 decision tree, pls, partial least squares; en, elastic net; knn, k-nearest neighbors; svm linear, support vector machine with linear kernel; rbf svm, support vector machine with rbf kernel. (DOCX 92 kb) [file 40168_2017_339_MOESM10_ESM.docx]

Figure S17: classifier results for metaphlan, surface


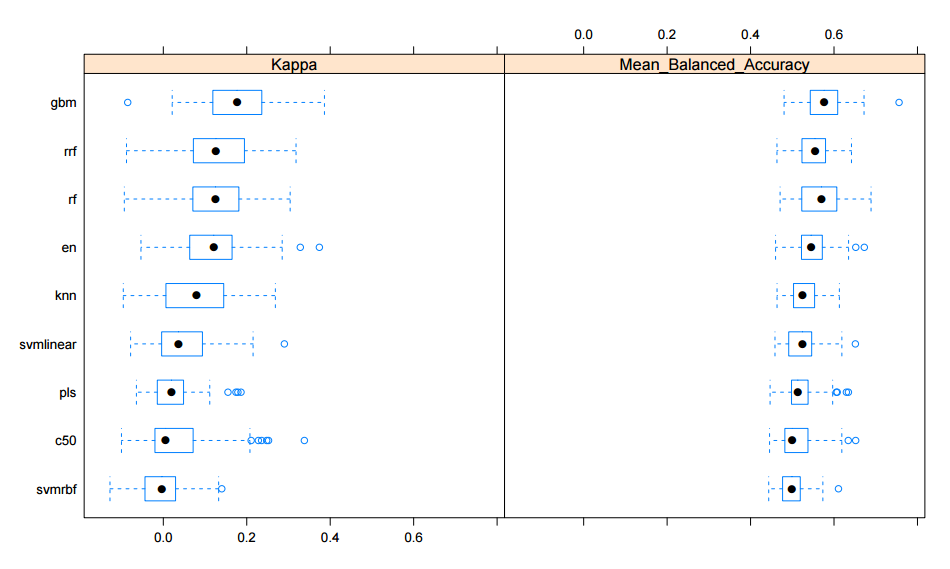

Supplement: Supplementary file 11 — Figure S17. Boxplots of classifier performance over model specific parameter sweeps during training (80/20 split) on MetaPhlAn2 data for surface class. Classes underwent down sampling and were optimized in terms of mean ROC score. Shown are kappa and balanced accuracy, averaged over classes. rf, random forest; gbm, stochastic gradient boosting; rrf, regularized random forest; c50, c5.0 decision tree, pls, partial least squares; en, elastic net; knn, k-nearest neighbors; svm linear, support vector machine with linear kernel; rbf svm, support vector machine with rbf kernel. (DOCX 96 kb) [file 40168_2017_339_MOESM11_ESM.docx]

Figure S18: classifier results for overlap, region


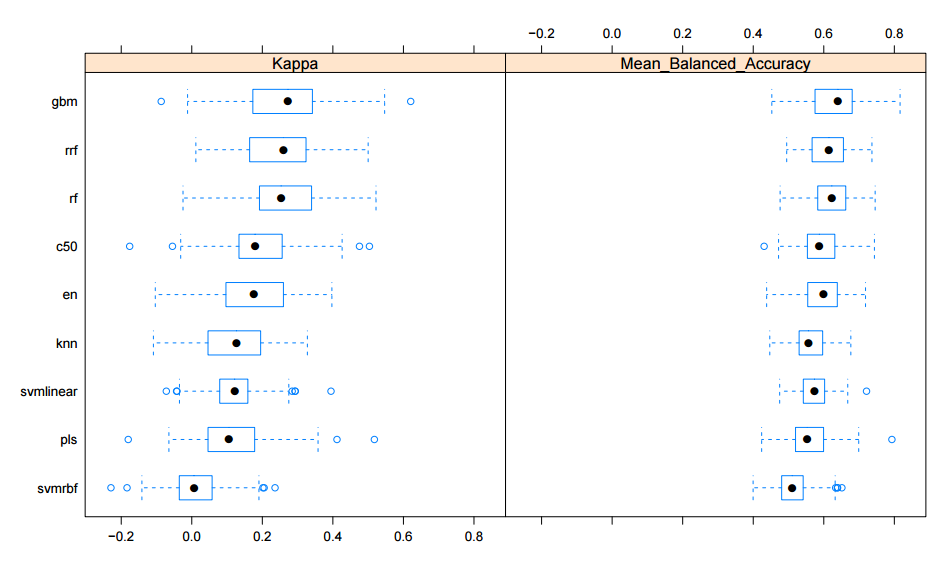

Supplement: Supplementary file 12 — Figure S18. Boxplots of classifier performance over model specific parameter sweeps during training (80/20 split) on overlap data for region class. Classes underwent up sampling and were optimized in terms of mean ROC score. Shown are kappa and balanced accuracy, averaged over classes. rf, random forest; gbm, stochastic gradient boosting; rrf, regularized random forest; c50, c5.0 decision tree, pls, partial least squares; en, elastic net; knn, k-nearest neighbors; svm linear, support vector machine with linear kernel; rbf svm, support vector machine with rbf kernel. (DOCX 101 kb) [file 40168_2017_339_MOESM12_ESM.docx]

Figure S19: classifier results for metaphlan, region


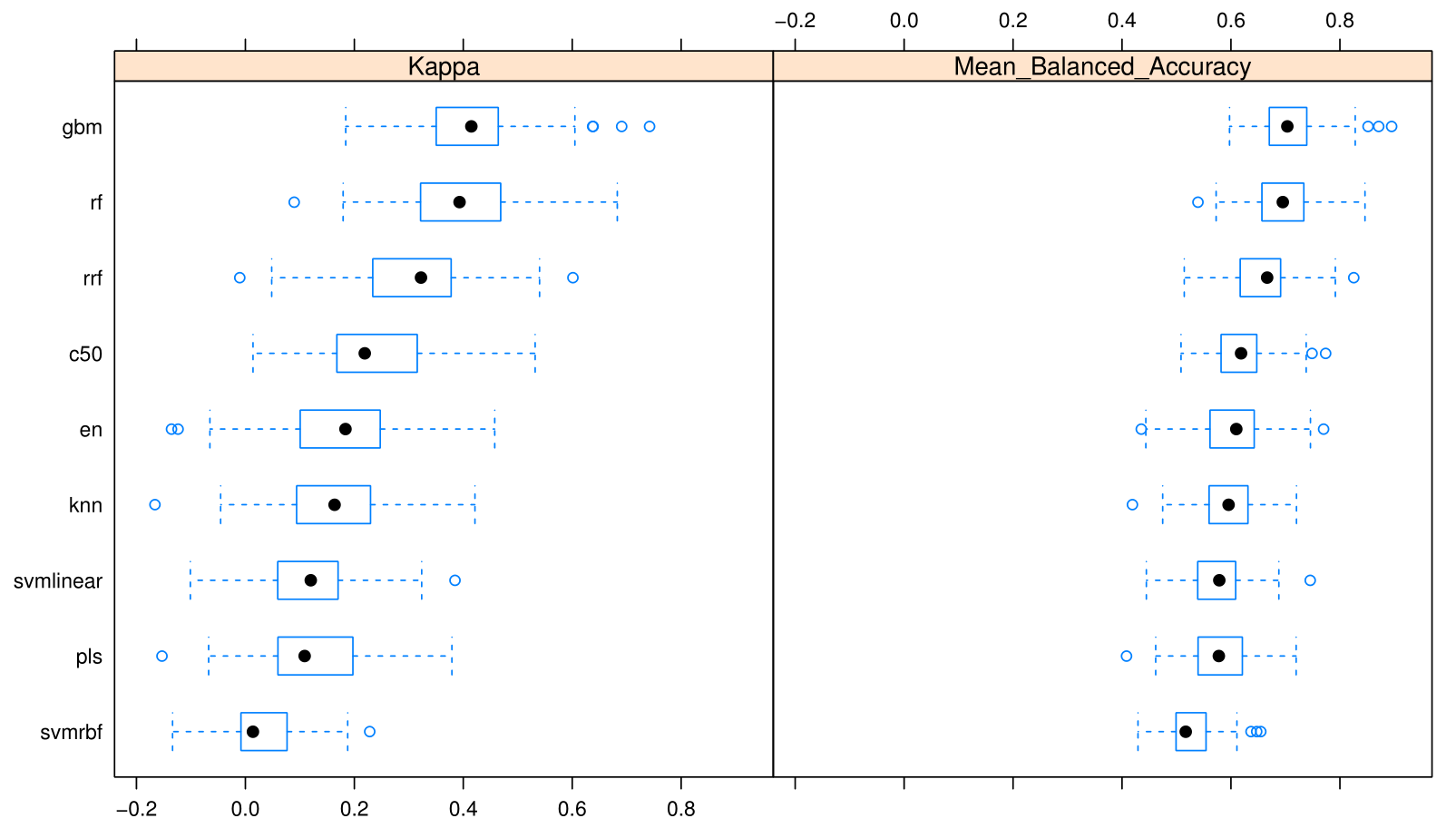

Supplement: Supplementary file 13 — Figure S19. Boxplots of classifier performance over model specific parameter sweeps during training (80/20 split) on MetaPhlAn2 data for region class. Classes underwent up sampling and were optimized in terms of mean ROC score. Shown are kappa and balanced accuracy, averaged over classes. rf, random forest; gbm, stochastic gradient boosting; rrf, regularized random forest; c50, c5.0 decision tree, pls, partial least squares; en, elastic net; knn, k-nearest neighbors; svm linear, support vector machine with linear kernel; rbf svm, support vector machine with rbf kernel. (DOCX 141 kb) [file 40168_2017_339_MOESM13_ESM.docx]

Figure S20: classifier results for overlap, city


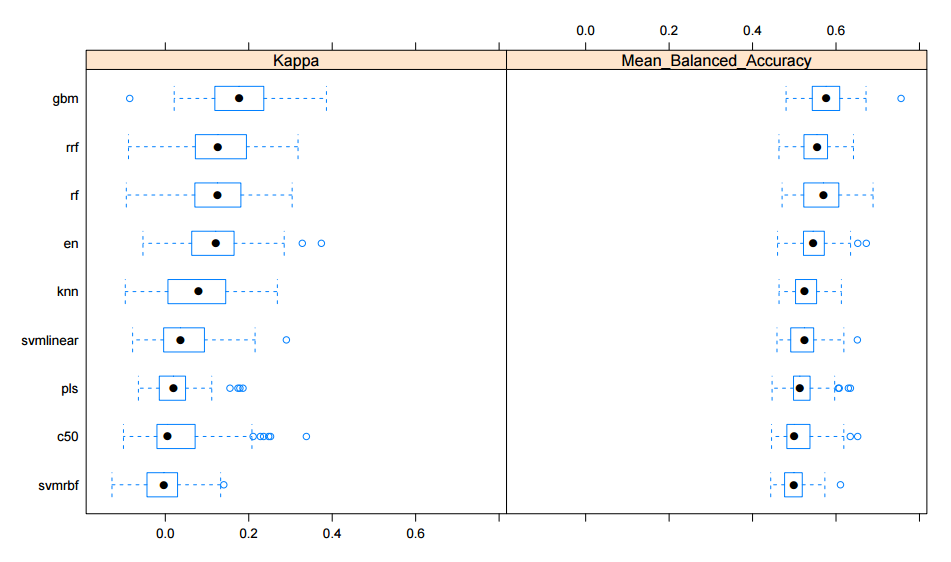

Supplement: Supplementary file 14 — Figure S20. Boxplots of classifier performance over model specific parameter sweeps during training (80/20 split) on overlap data for city class. Classes underwent up sampling and were optimized in terms of mean ROC score. Shown are kappa and balanced accuracy, averaged over classes. rf, random forest; gbm, stochastic gradient boosting; rrf, regularized random forest; c50, c5.0 decision tree, pls, partial least squares; en, elastic net; knn, k-nearest neighbors; svm linear, support vector machine with linear kernel; rbf svm, support vector machine with rbf kernel. (DOCX 97 kb) [file 40168_2017_339_MOESM14_ESM.docx]

Figure S22: classifier results for all datasets, surface


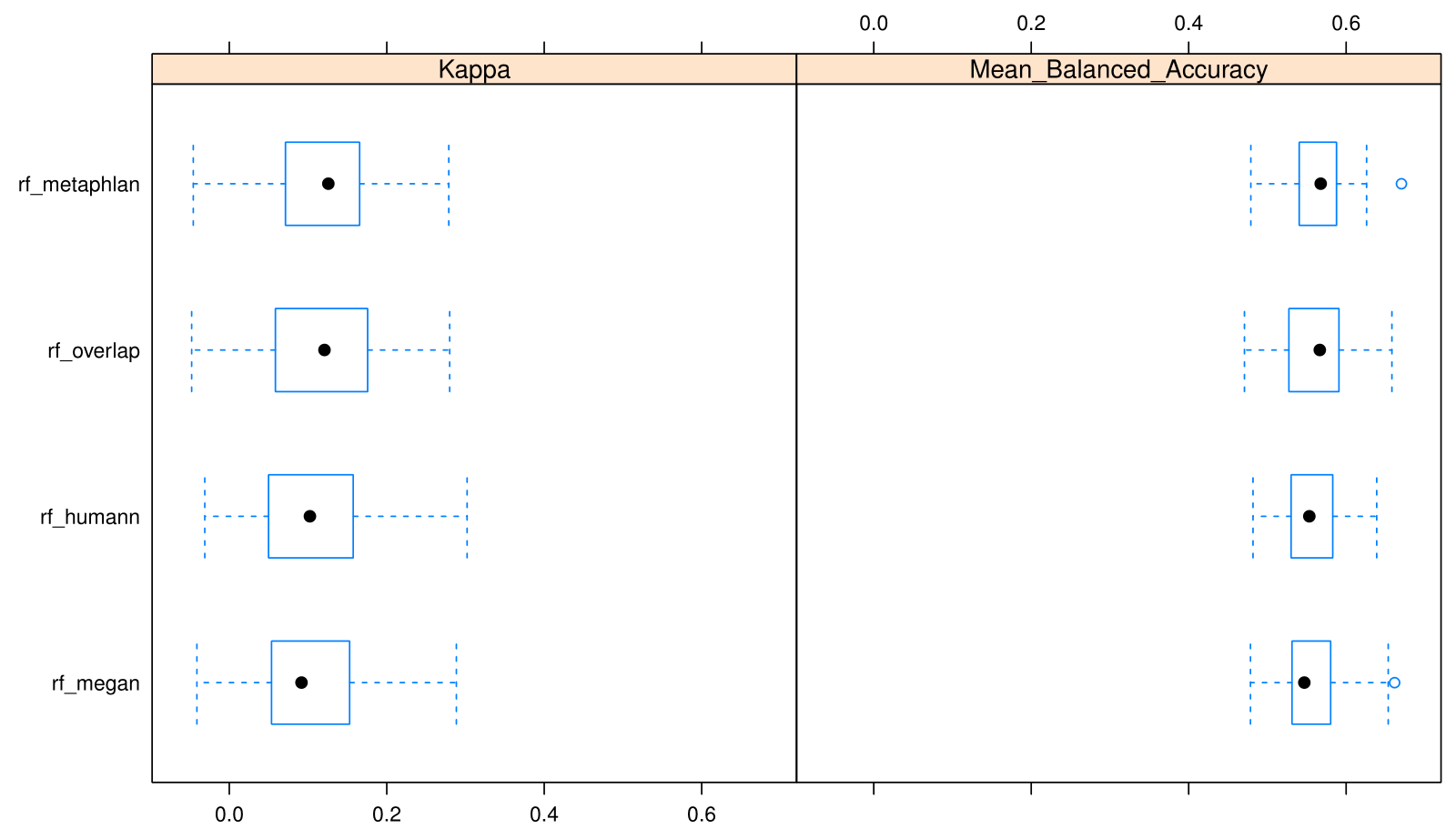

Supplement: Supplementary file 15 — Figure S21. Boxplots of classifier performance over model specific parameter sweeps during training (80/20 split) on MetaPhlan data for city class. Classes underwent up sampling and were optimized in terms of mean ROC score. Shown are kappa and balanced accuracy, averaged over classes. rf, random forest; gbm, stochastic gradient boosting; rrf, regularized random forest; c50, c5.0 decision tree, pls, partial least squares; en, elastic net; knn, k-nearest neighbors; svm linear, support vector machine with linear kernel; rbf svm, support vector machine with rbf kernel. (DOCX 134 kb) [file 40168_2017_339_MOESM15_ESM.docx]

Figure S21: classifier results for metaphlan, city


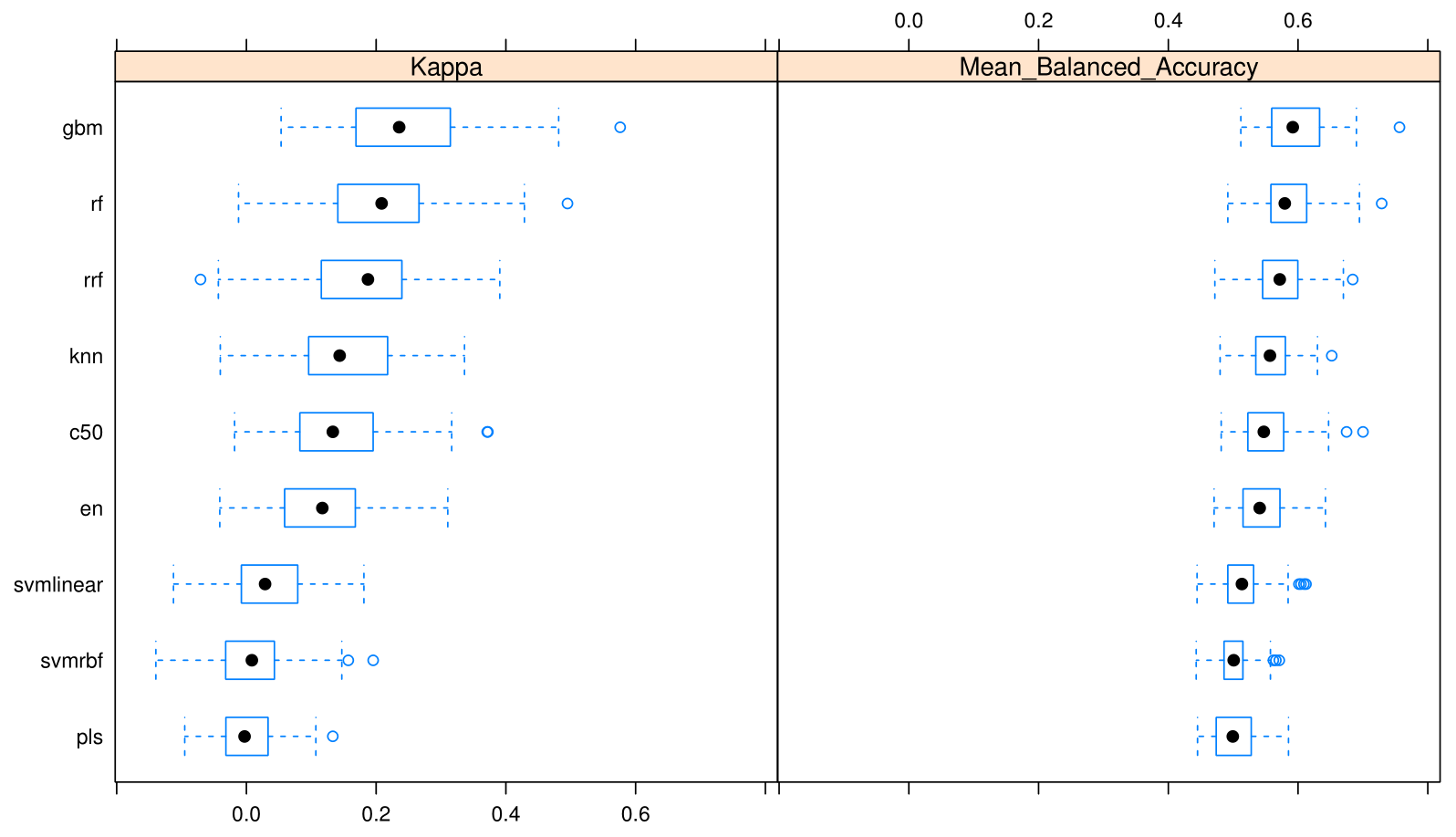

Supplement: Supplementary file 16 — Figure S22. Boxplots of dataset performance for random forest training (80/20 split) for surface class. Classes underwent down sampling and were optimized in terms of mean ROC score. Shown are kappa and balanced accuracy, averaged over classes. (DOCX 107 kb) [file 40168_2017_339_MOESM16_ESM.docx]

Figure S23: classifier results for all datasets, region


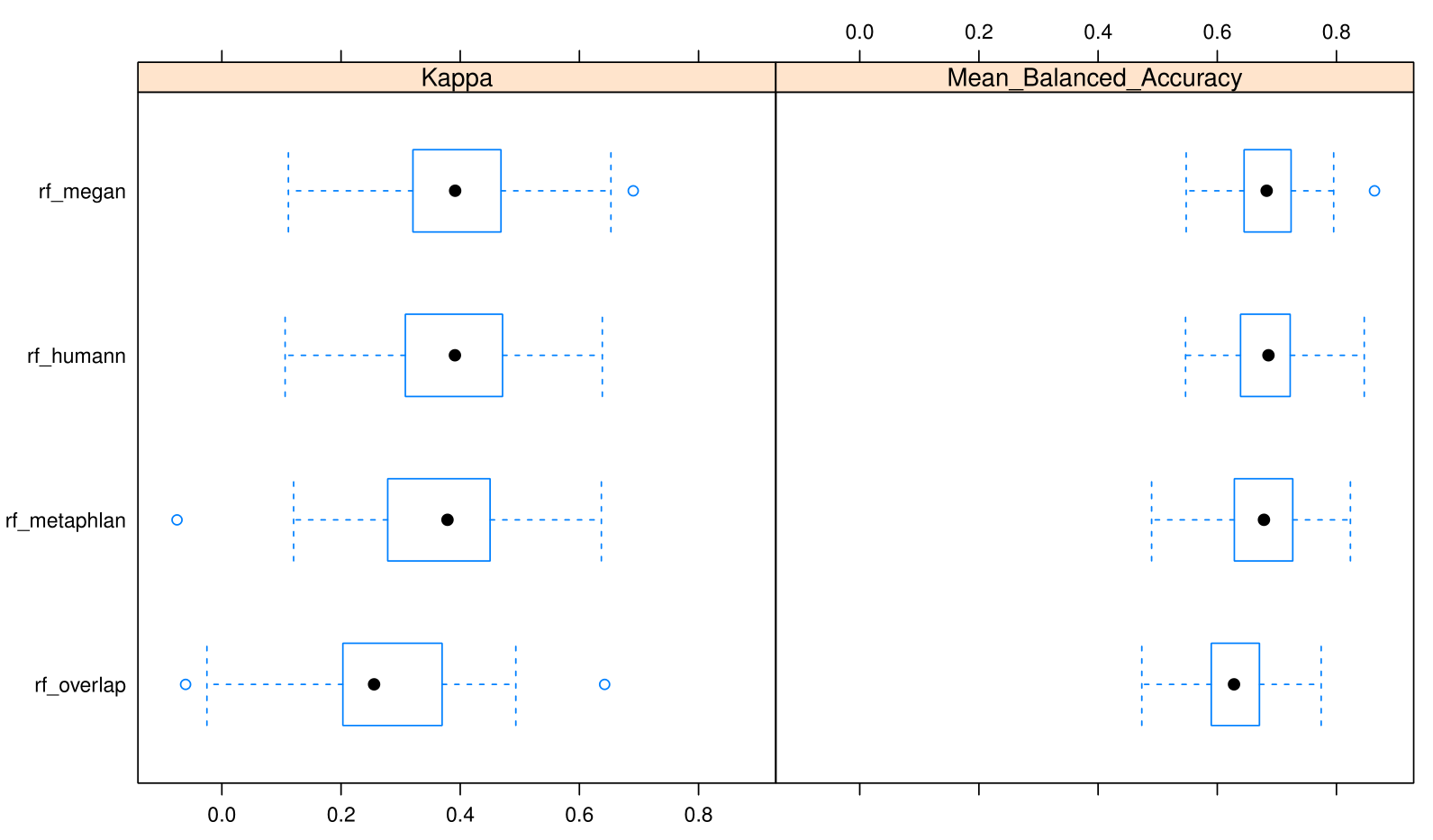

Supplement: Supplementary file 17 — Figure S23. Boxplots of dataset performance for random forest training (80/20 split) for region class. Classes underwent down sampling and were optimized in terms of mean ROC score. Shown are kappa and balanced accuracy, averaged over classes. (DOCX 115 kb) [file 40168_2017_339_MOESM17_ESM.docx]

Figure S24: classifier results for all datasets, city


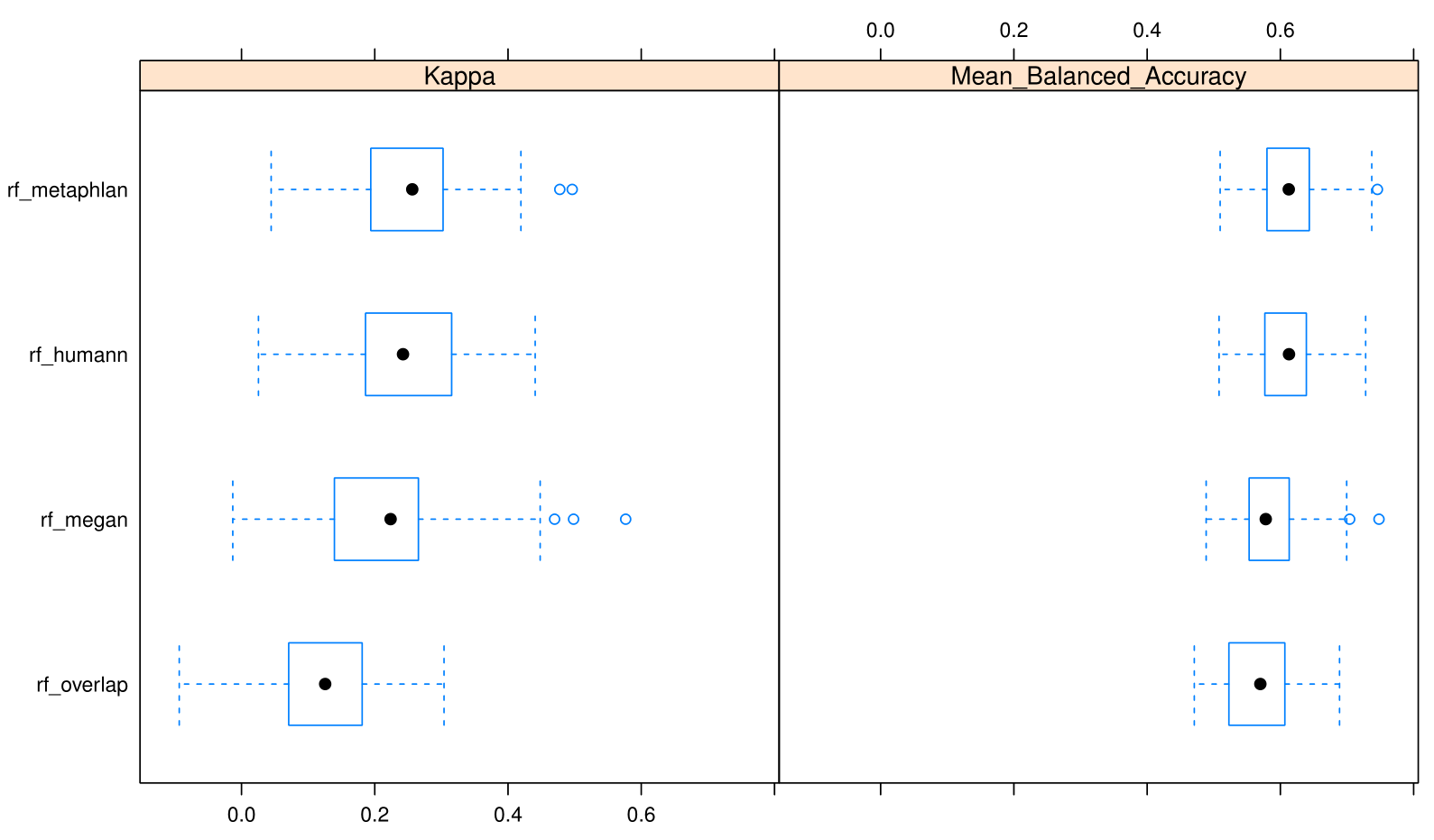

Supplement: Supplementary file 18 — Figure S24. Boxplots of dataset performance for random forest training (80/20 split) for city class. Classes underwent down sampling and were optimized in terms of mean ROC score. Shown are kappa and balanced accuracy, averaged over classes. (DOCX 113 kb) [file 40168_2017_339_MOESM18_ESM.docx]

Figure S25: ROC curve, overlap, front rear


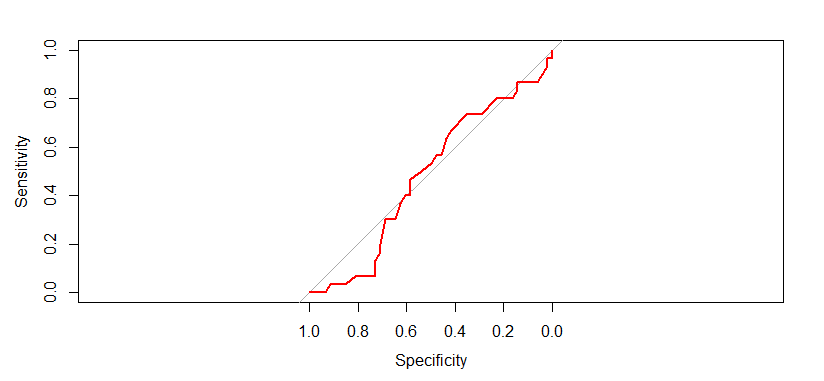

Supplement: Supplementary file 19 — Figure S25. ROC curve of random forest test set performance (80/20 split) on front-rear surface class. Classes underwent down sampling and were optimized in terms of ROC score. (DOCX 51 kb) [file 40168_2017_339_MOESM19_ESM.docx]

Figure S10: Shannon diversity, overlap, region


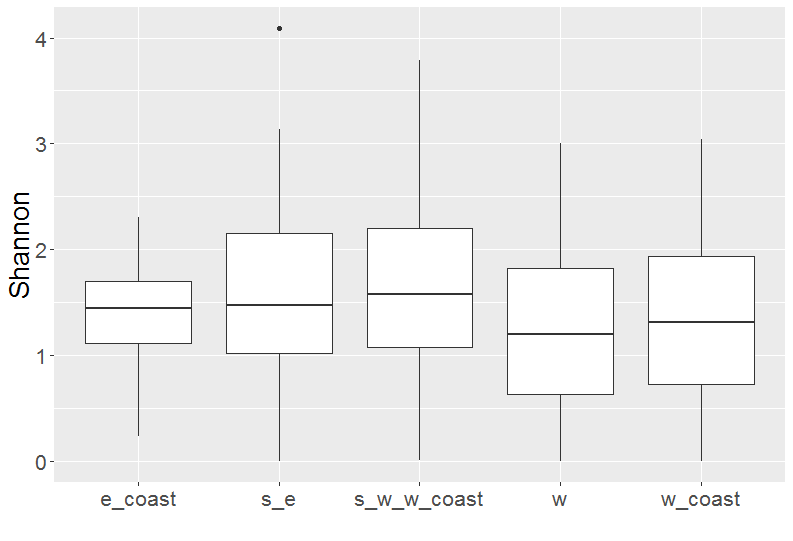

Supplement: Supplementary file 27 — Figure S10. Shannon alpha diversity for overlap data. (DOCX 25 kb) [file 40168_2017_339_MOESM27_ESM.docx]

Figure S11: Correlation between overlap and humann prevalence


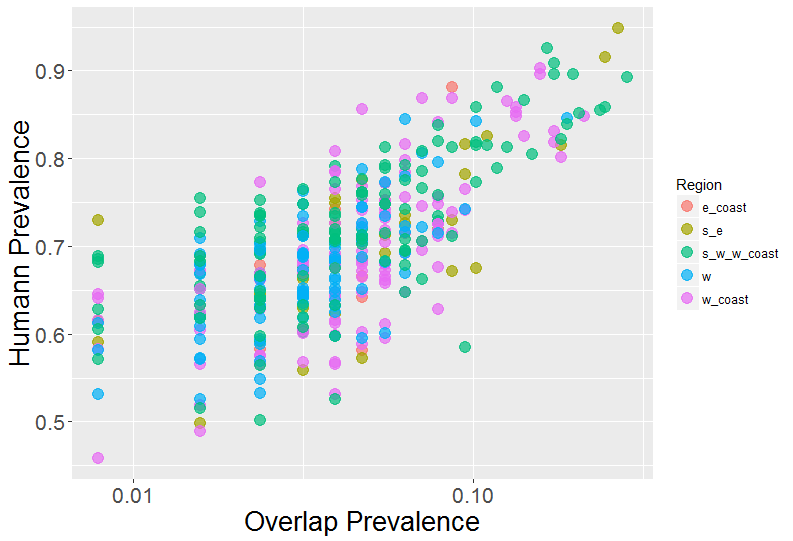

Supplement: Supplementary file 28 — Figure S11. Scatter plot of HUMAnN2 gene prevalence (proportion of pathways found in sample i) versus overlap species prevalence (proportion of species found in sample i). Each point represents a sample, with colors representing the sample region. (DOCX 120 kb) [file 40168_2017_339_MOESM28_ESM.docx]

Figure S13: Sequence coverage across *S. aureus* genome and mecA for sample with highest abundance.


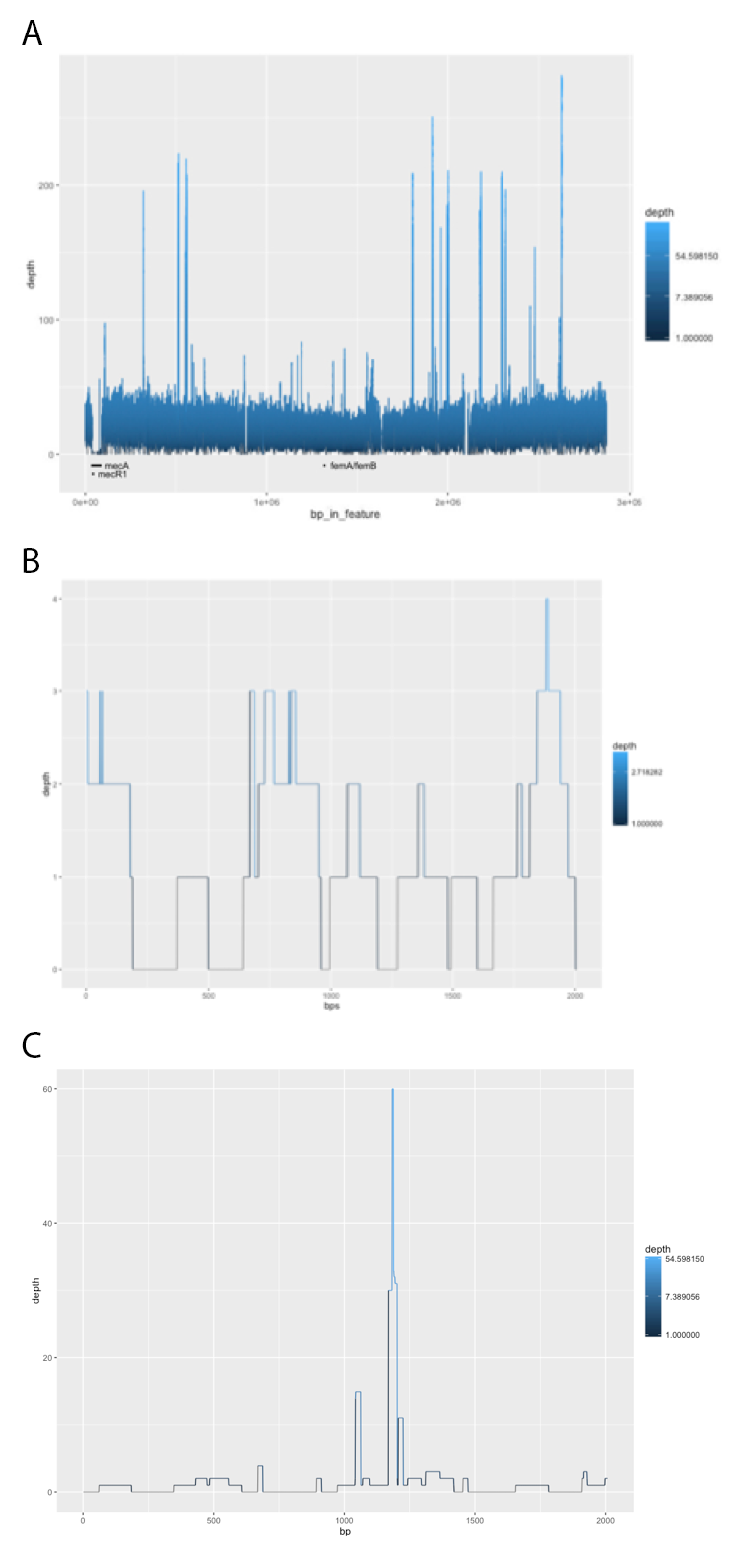

Supplement: Supplementary file 31 — Figure S13. Evidence of non-MRSA S. aureus found in ambulance samples. (A) Visualization of sequence coverage across genome of sample with highest relative abundance of S. aureus (AW0974) shows lack of coverage over mecA and high consistent coverage over femA and femB (gene locations marked on X-axis) and most of the genome (average 20× coverage ranging from 0 to 174). (B-C) Sequence coverage over mecA for the two S. aureus positive samples with the highest level of mecA coverage shows lack of evidence for MRSA. (DOCX 3569 kb) [file 40168_2017_339_MOESM31_ESM.docx]
