# Supplementary material for: Metagenomic characterization of ambulances across the USA
Source: Microbiome. 2017 Sep 22;5:125. doi: 10.1186/s40168-017-0339-6 (PMC5610413; doi:10.1186/s40168-017-0339-6)
Supplement: Supplementary file 22 — Figure S6. Normalized feature important for overlap data during random forest training (80/20 split) for city class. Classes underwent up sampling and were optimized in terms of mean ROC score. Shown are kappa and balanced accuracy, averaged over classes. (DOCX 553 kb) [file 40168_2017_339_MOESM22_ESM.docx]

Figure S6: random forest importance, overlap, city
