# Supplementary material for: Metagenomic characterization of ambulances across the USA
Source: Microbiome. 2017 Sep 22;5:125. doi: 10.1186/s40168-017-0339-6 (PMC5610413; doi:10.1186/s40168-017-0339-6)
Supplement: Supplementary file 23 — Figure S7. Overlap binned abundances (RPK) over samples for the top 3 ranking species (columns) in terms of feature importance from random forest classification training (80/20 split, 128 trees). Red bars correspond to the top ranking feature for that deidentified city (row). (DOCX 452 kb) [file 40168_2017_339_MOESM23_ESM.docx]

Figure S7: random forest, importance abundance, overlap, city
