# Supplementary material for: Metagenomic characterization of ambulances across the USA
Source: Microbiome. 2017 Sep 22;5:125. doi: 10.1186/s40168-017-0339-6 (PMC5610413; doi:10.1186/s40168-017-0339-6)

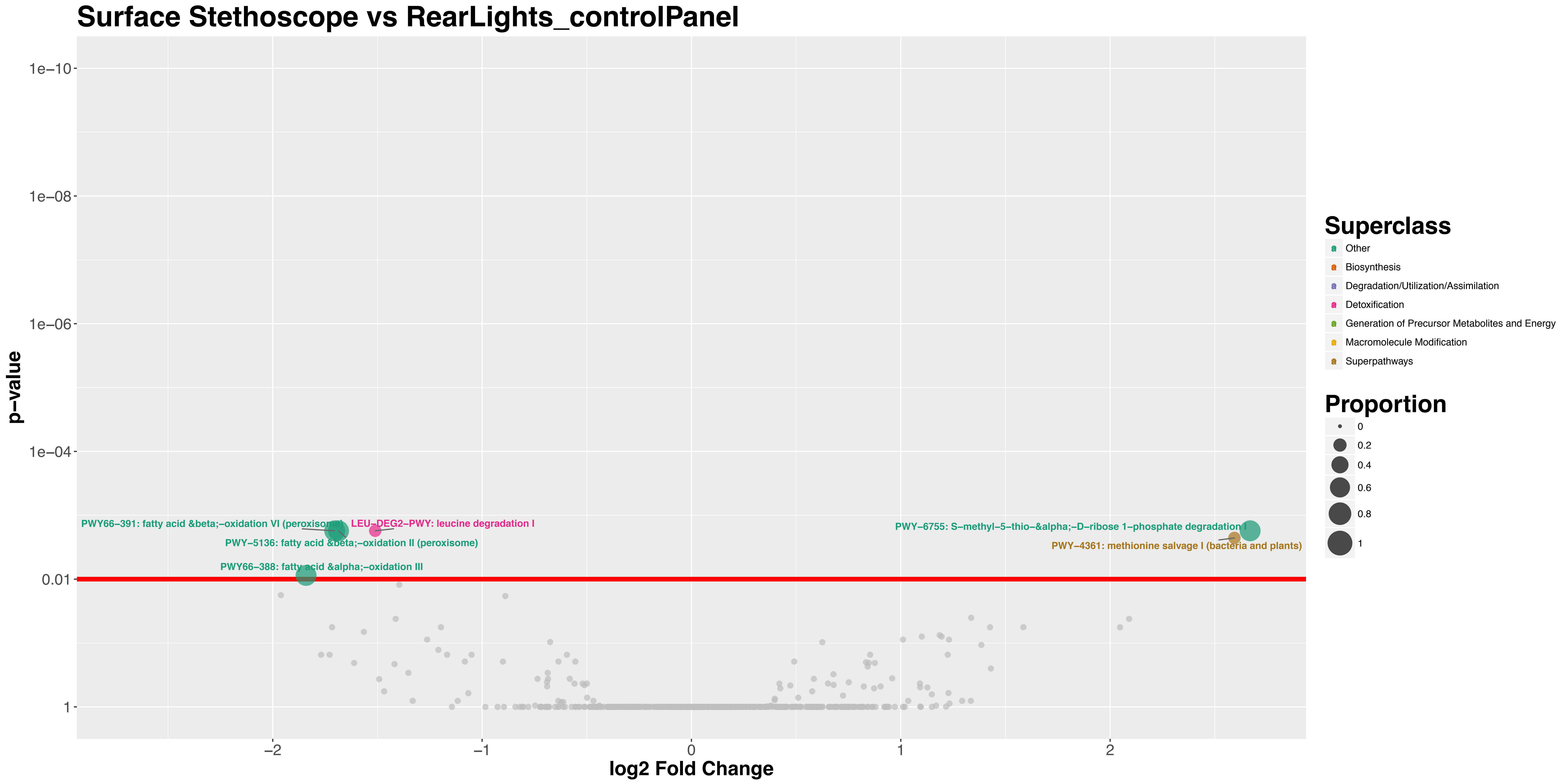
Surface Stethoscope vs RearLights_controlPanel
p−value
1e−10
1e−08
1e−06
1e−04
0.01
1
−2
−1
0
1
2
log2 Fold Change
PWY66−391: fatty acid β−oxidation VI (peroxisome)
LEU−DEG2−PWY: leucine degradation I
PWY−5136: fatty acid β−oxidation II (peroxisome)
PWY66−388: fatty acid α−oxidation III
PWY−6755: S−methyl−5−thio−α−D−ribose 1−phosphate degradation I
PWY−4361: methionine salvage I (bacteria and plants)
Superclass
Other
Biosynthesis
Degradation/Utilization/Assimilation
Detoxification
Generation of Precursor Metabolites and Energy
Macromolecule Modification
Superpathways
Proportion
0
0.2
0.4
0.6
0.8
1

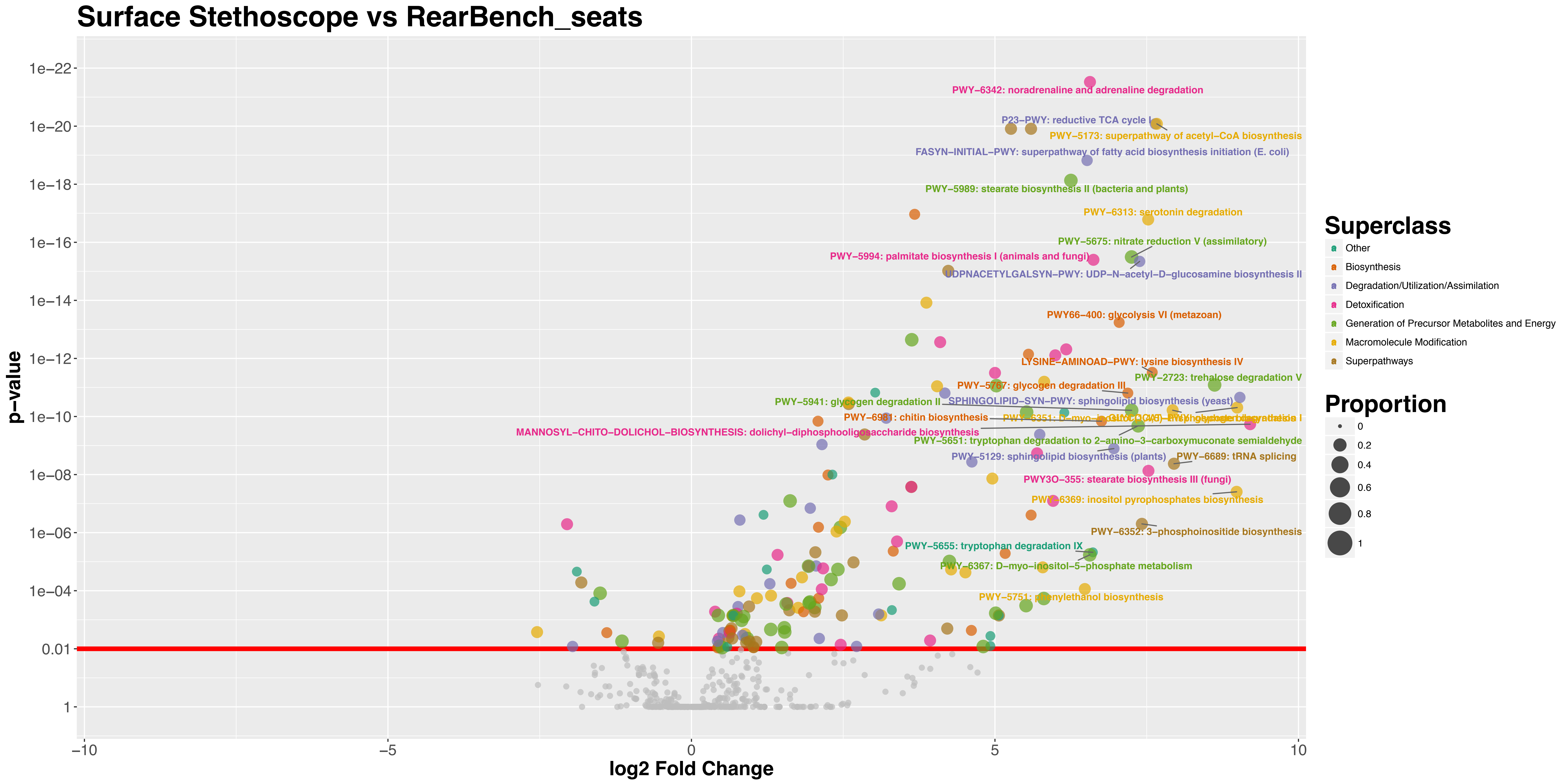
Surface Stethoscope vs RearBench_seats
p−value
log2 Fold Change
1e−22
1e−20
1e−18
1e−16
1e−14
1e−12
1e−10
1e−08
1e−06
1e−04
0.01
1
−10
−5
0
5
10
PWY−6342: noradrenaline and adrenaline degradation
P23−PWY: reductive TCA cycle I
PWY−5173: superpathway of acetyl−CoA biosynthesis
FASYN−INITIAL−PWY: superpathway of fatty acid biosynthesis initiation (E. coli)
PWY−5989: stearate biosynthesis II (bacteria and plants)
PWY−6313: serotonin degradation
PWY−5675: nitrate reduction V (assimilatory)
PWY−5994: palmitate biosynthesis I (animals and fungi)
UDPNACETYLGALSYN−PWY: UDP−N−acetyl−D−glucosamine biosynthesis II
PWY66−400: glycolysis VI (metazoan)
LYSINE−AMINOAD−PWY: lysine biosynthesis IV
PWY−2723: trehalose degradation V
PWY−5767: glycogen degradation III
PWY−5941: glycogen degradation II
SPHINGOLIPID−SYN−PWY: sphingolipid biosynthesis (yeast)
PWY−6981: chitin biosynthesis
MANNOSYL−CHITO−DOLICHOL−BIOSYNTHESIS: dolichyl−diphosphooligosaccharide biosynthesis
PWY−5651: tryptophan degradation to 2−amino−3−carboxymuconate semialdehyde
PWY−5129: sphingolipid biosynthesis (plants)
PWY−6689: tRNA splicing
PWY3O−355: stearate biosynthesis III (fungi)
PWY−6369: inositol pyrophosphates biosynthesis
PWY−6352: 3−phosphoinositide biosynthesis
PWY−5655: tryptophan degradation IX
PWY−6367: D−myo−inositol−5−phosphate metabolism
PWY−5751: phenylethanol biosynthesis
Superclass
Other
Biosynthesis
Degradation/Utilization/Assimilation
Detoxification
Generation of Precursor Metabolites and Energy
Macromolecule Modification
Superpathways
Proportion
0
0.2
0.4
0.6
0.8
1

Surface RearLights_controlPanel vs RearBench_seats

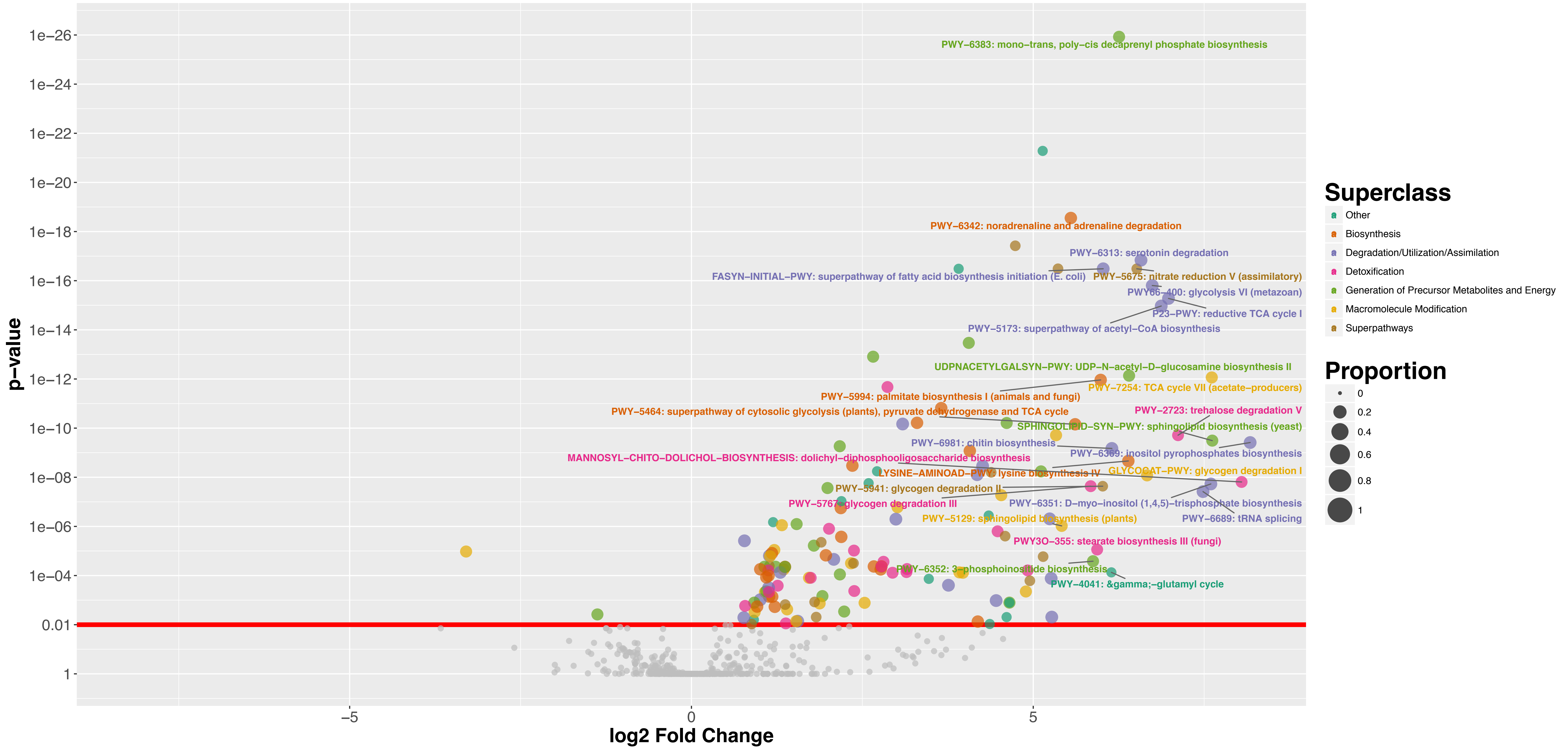

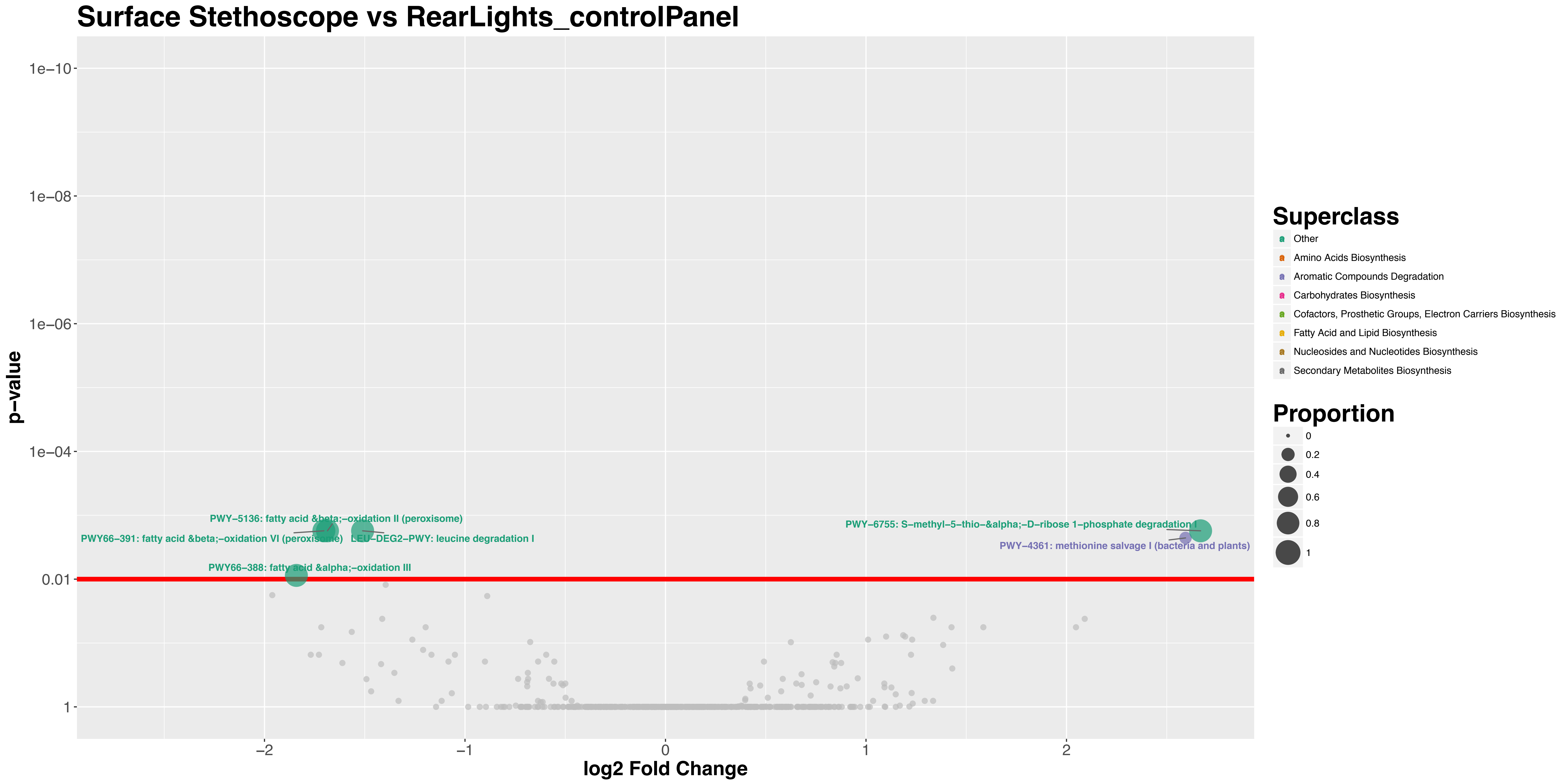

Surface Stethoscope vs RearLights_controlPanel
p−value
1e−10
1e−08
1e−06
1e−04
0.01
1
log2 Fold Change
−2
−1
0
1
2
PWY−5136: fatty acid β−oxidation II (peroxisome)
PWY66−391: fatty acid β−oxidation VI (peroxisome)
LEU−DEG2−PWY: leucine degradation I
PWY66−388: fatty acid α−oxidation III
PWY−6755: S−methyl−5−thio−α−D−ribose 1−phosphate degradation I
PWY−4361: methionine salvage I (bacteria and plants)
Superclass
Other
Amino Acids Biosynthesis
Aromatic Compounds Degradation
Carbohydrates Biosynthesis
Cofactors, Prosthetic Groups, Electron Carriers Biosynthesis
Fatty Acid and Lipid Biosynthesis
Nucleosides and Nucleotides Biosynthesis
Secondary Metabolites Biosynthesis
Proportion
0
0.2
0.4
0.6
0.8
1

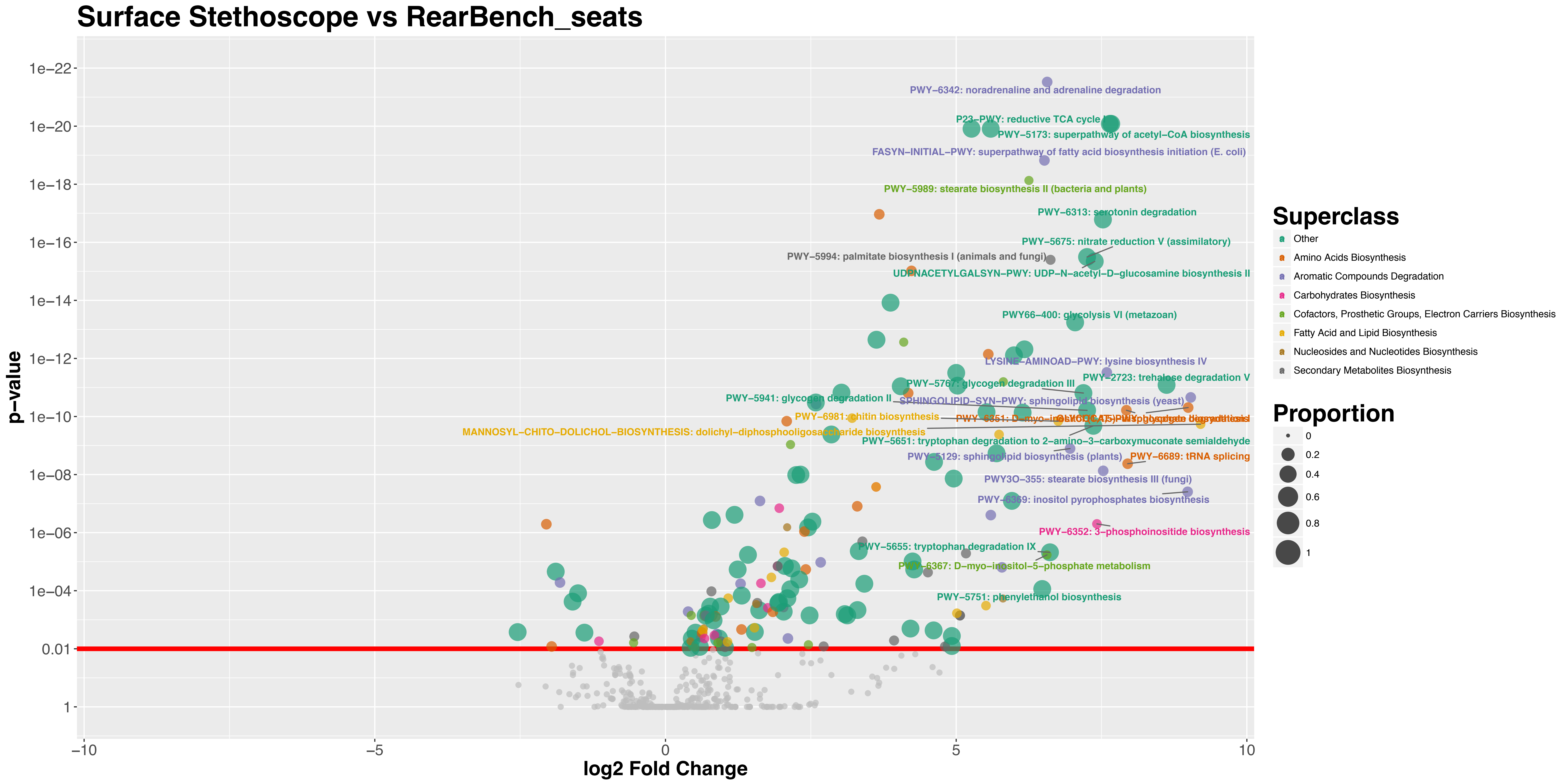
Surface Stethoscope vs RearBench_seats
PWY−6342: noradrenaline and adrenaline degradation
P23−PWY: reductive TCA cycle I
PWY−5173: superpathway of acetyl−CoA biosynthesis
FASYN−INITIAL−PWY: superpathway of fatty acid biosynthesis initiation (E. coli)
PWY−5989: stearate biosynthesis II (bacteria and plants)
PWY−6313: serotonin degradation
PWY−5675: nitrate reduction V (assimilatory)
PWY−5994: palmitate biosynthesis I (animals and fungi)
UDPNACETYLGALSYN−PWY: UDP−N−acetyl−D−glucosamine biosynthesis II
PWY66−400: glycolysis VI (metazoan)
LYSINE−AMINOAD−PWY: lysine biosynthesis IV
PWY−2723: trehalose degradation V
PWY−5767: glycogen degradation III
PWY−5941: glycogen degradation II
SPHINGOLIPID−SYN−PWY: sphingolipid biosynthesis (yeast)
PWY−6981: chitin biosynthesis
MANNOSYL−CHITO−DOLICHOL−BIOSYNTHESIS: dolichyl−diphosphooligosaccharide biosynthesis
PWY−5651: tryptophan degradation to 2−amino−3−carboxymuconate semialdehyde
PWY−5129: sphingolipid biosynthesis (plants)
PWY−6689: tRNA splicing
PWY3O−355: stearate biosynthesis III (fungi)
PWY−6369: inositol pyrophosphates biosynthesis
PWY−6352: 3−phosphoinositide biosynthesis
PWY−5655: tryptophan degradation IX
PWY−6367: D−myo−inositol−5−phosphate metabolism
PWY−5751: phenylethanol biosynthesis
p−value
1e−22
1e−20
1e−18
1e−16
1e−14
1e−12
1e−10
1e−08
1e−06
1e−04
0.01
1
−10
−5
0
5
10
log2 Fold Change
Superclass
Other
Amino Acids Biosynthesis
Aromatic Compounds Degradation
Carbohydrates Biosynthesis
Cofactors, Prosthetic Groups, Electron Carriers Biosynthesis
Fatty Acid and Lipid Biosynthesis
Nucleosides and Nucleotides Biosynthesis
Secondary Metabolites Biosynthesis
Proportion
0
0.2
0.4
0.6
0.8
1

Surface RearLights_controlPanel vs RearBench_seats

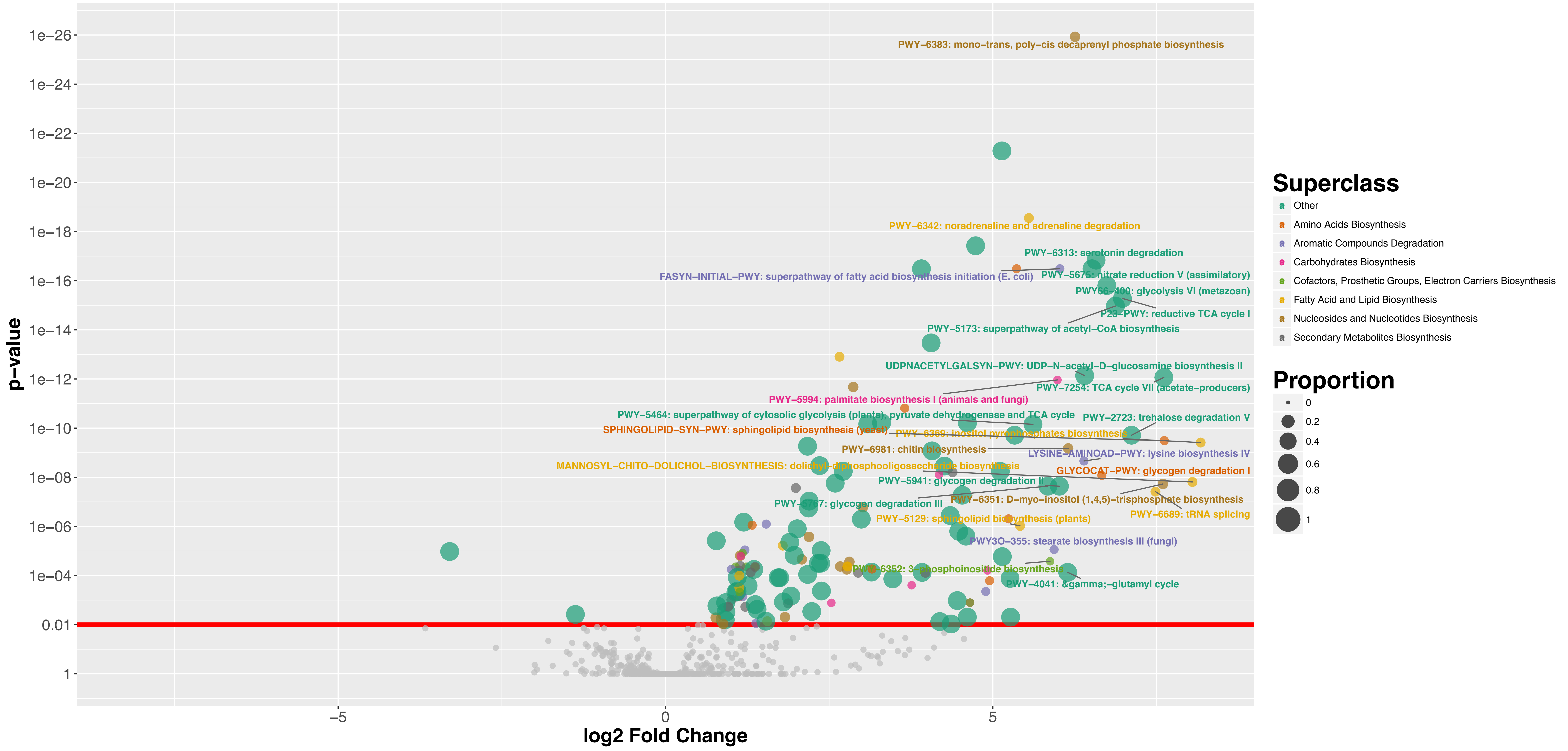

Supplement: Supplementary file 25 — Figure S8. Volcano plot of the p-value versus log2-fold change (LFC) of HUMAnN2 pathway abundances resulting from a DESeq2 differential abundance analysis for surface class with FDR correction (Benjamini-Hochberg correction, α = 0.01). Class combinations were selected based on overlap data classification performance. Points vary in color based on pathway superclass and size based on the proportion of genes in that class with p < α. Genes in the 95th percentile of absolute LFC are labeled. (PDF 273 kb) [file 40168_2017_339_MOESM25_ESM.pdf]
