# Supplementary material for: Metagenomic characterization of ambulances across the USA
Source: Microbiome. 2017 Sep 22;5:125. doi: 10.1186/s40168-017-0339-6 (PMC5610413; doi:10.1186/s40168-017-0339-6)
Supplement: Supplementary file 26 — Figure S9. Volcano plot of the p value versus log2-fold change (LFC) of HUMAnN2 pathway abundances resulting from a DESeq2 differential abundance analysis for region class with FDR correction (Benjamini-Hochberg correction, α = 0.01). Class combinations were selected based on overlap data classification performance. Points vary in color based on pathway superclass and size based on the proportion of genes in that class with p < α. Genes in the 95th percentile of absolute LFC are labeled. (PDF 381 kb) [file 40168_2017_339_MOESM26_ESM.pdf]

region s_w_w_coast vs s_e

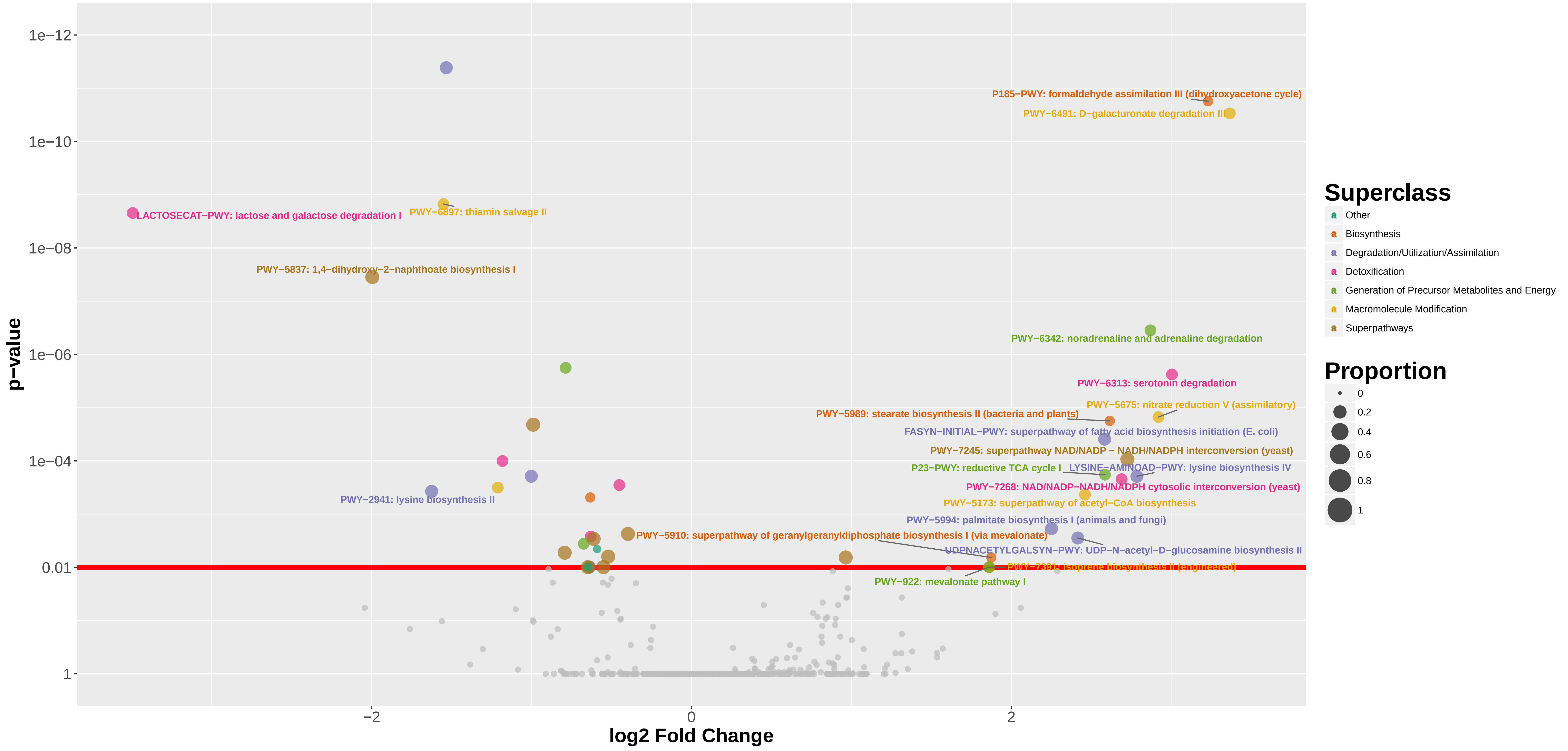

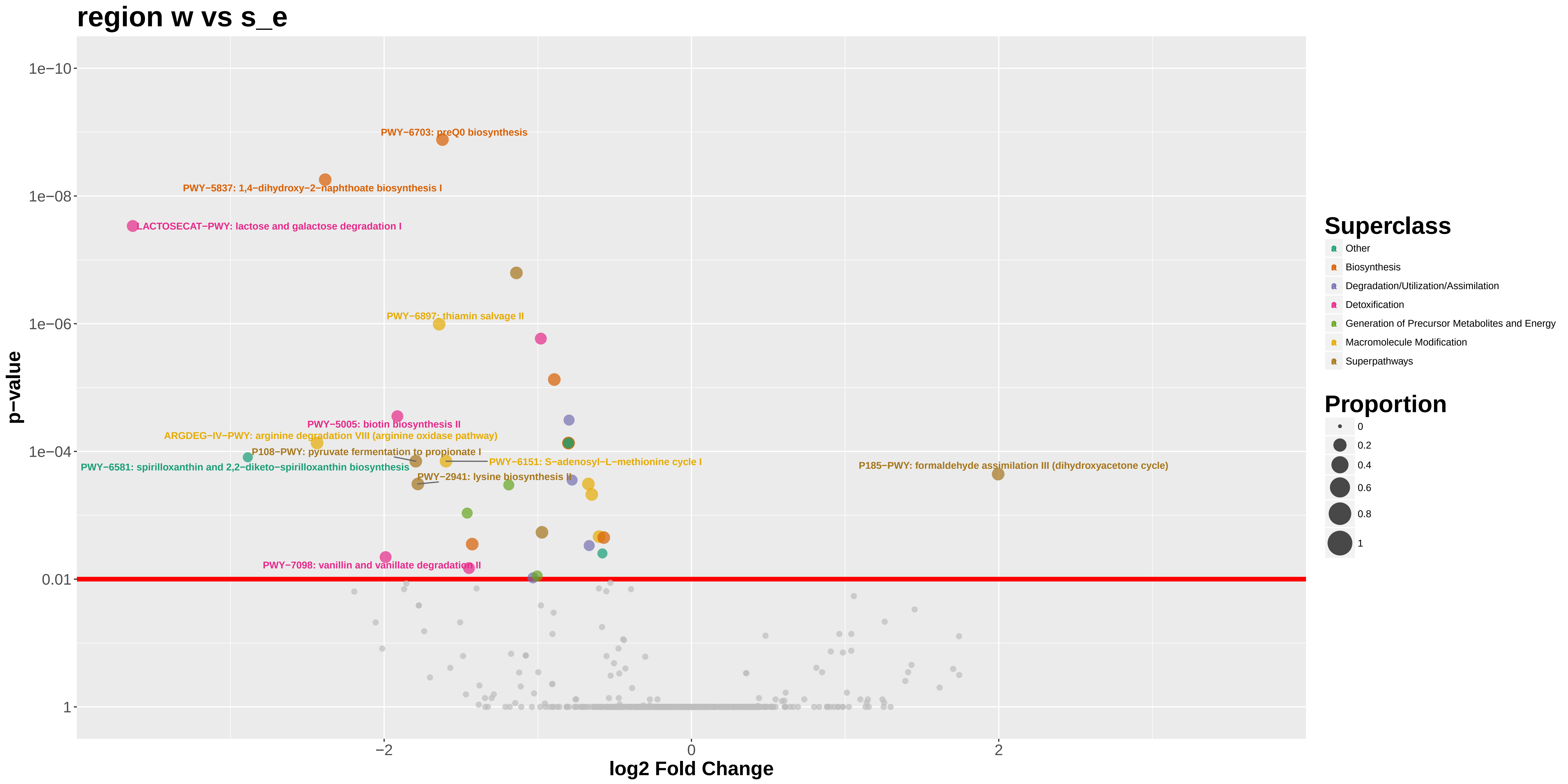
region w vs s_e
PWY−6703: preQ0 biosynthesis
PWY−5837: 1,4−dihydroxy−2−naphthoate biosynthesis I
LACTOSECAT−PWY: lactose and galactose degradation I
PWY−6897: thiamin salvage II
PWY−5005: biotin biosynthesis II
ARGDEG−IV−PWY: arginine degradation VIII (arginine oxidase pathway)
P108−PWY: pyruvate fermentation to propionate I
PWY−6151: S−adenosyl−L−methionine cycle I
PWY−6581: spirilloxanthin and 2,2'−diketo−spirilloxanthin biosynthesis
PWY−2941: lysine biosynthesis II
P185−PWY: formaldehyde assimilation III (dihydroxyacetone cycle)
PWY−7098: vanillin and vanillate degradation II
p−value
1e−10
1e−08
1e−06
1e−04
0.01
1
log2 Fold Change
−2
0
2
Superclass
Other
Biosynthesis
Degradation/Utilization/Assimilation
Detoxification
Generation of Precursor Metabolites and Energy
Macromolecule Modification
Superpathways
Proportion
0
0.2
0.4
0.6
0.8
1

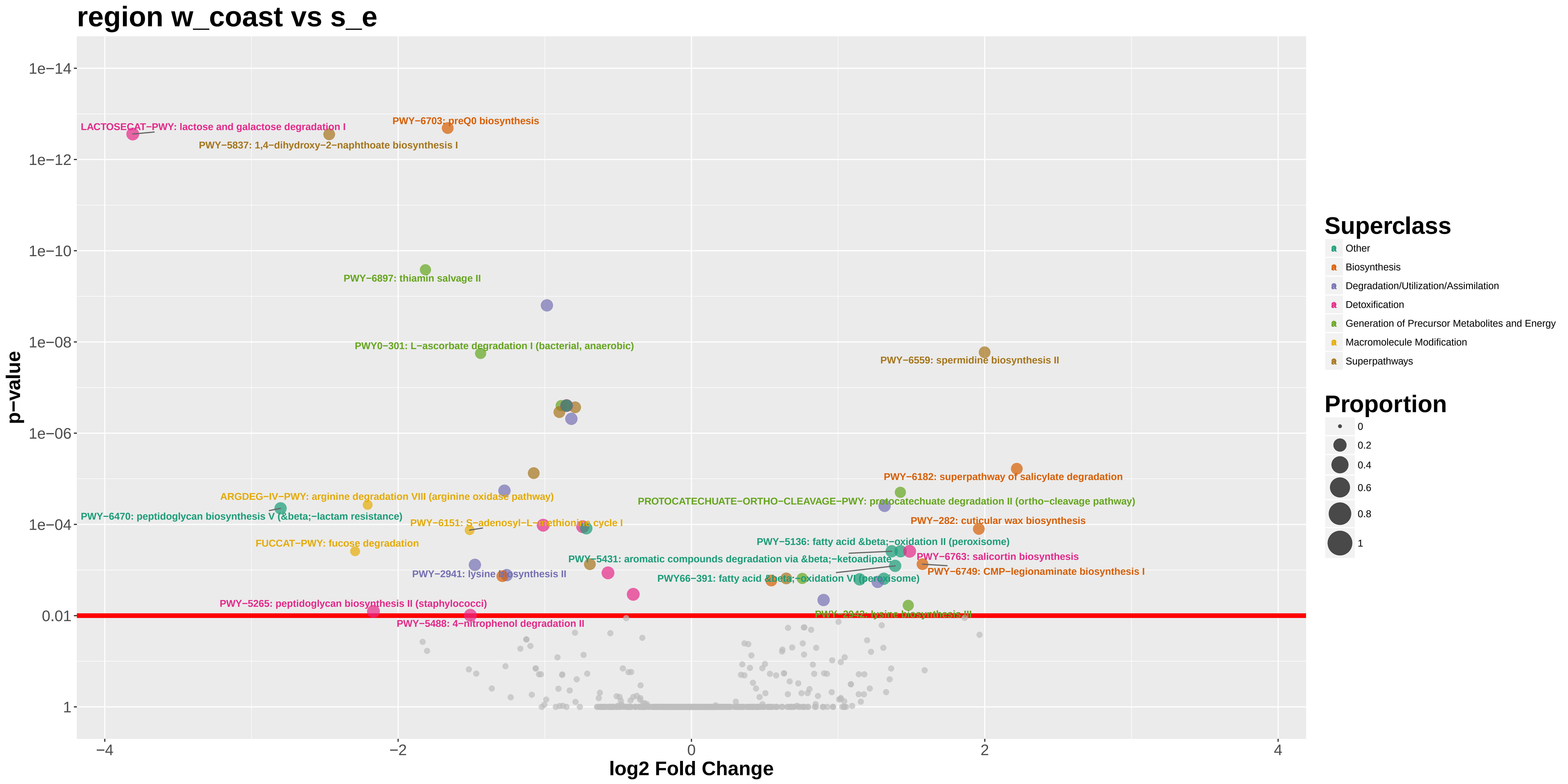
region w_coast vs s_e
p−value
1e−14
1e−12
1e−10
1e−08
1e−06
1e−04
0.01
1
log2 Fold Change
−4
−2
0
2
4
LACTOSECAT−PWY: lactose and galactose degradation I
PWY−6703: preQ0 biosynthesis
PWY−5837: 1,4−dihydroxy−2−naphthoate biosynthesis I
PWY−6897: thiamin salvage II
PWY0−301: L−ascorbate degradation I (bacterial, anaerobic)
PWY−6559: spermidine biosynthesis II
PWY−6182: superpathway of salicylate degradation
ARGDEG−IV−PWY: arginine degradation VIII (arginine oxidase pathway)
PROTOCATECHUATE−ORTHO−CLEAVAGE−PWY: protocatechuate degradation II (ortho−cleavage pathway)
PWY−6470: peptidoglycan biosynthesis V (β−lactam resistance)
PWY−282: cuticular wax biosynthesis
PWY−6151: S−adenosyl−L−methionine cycle I
FUCCAT−PWY: fucose degradation
PWY−5136: fatty acid β−oxidation II (peroxisome)
PWY−5431: aromatic compounds degradation via β−ketoadipate
PWY−6763: salicortin biosynthesis
PWY−2941: lysine biosynthesis II
PWY−6749: CMP−legionaminate biosynthesis I
PWY66−391: fatty acid β−oxidation VI (peroxisome)
PWY−5265: peptidoglycan biosynthesis II (staphylococci)
PWY−2942: lysine biosynthesis III
PWY−5488: 4−nitrophenol degradation II
Superclass
Other
Biosynthesis
Degradation/Utilization/Assimilation
Detoxification
Generation of Precursor Metabolites and Energy
Macromolecule Modification
Superpathways
Proportion
0
0.2
0.4
0.6
0.8
1

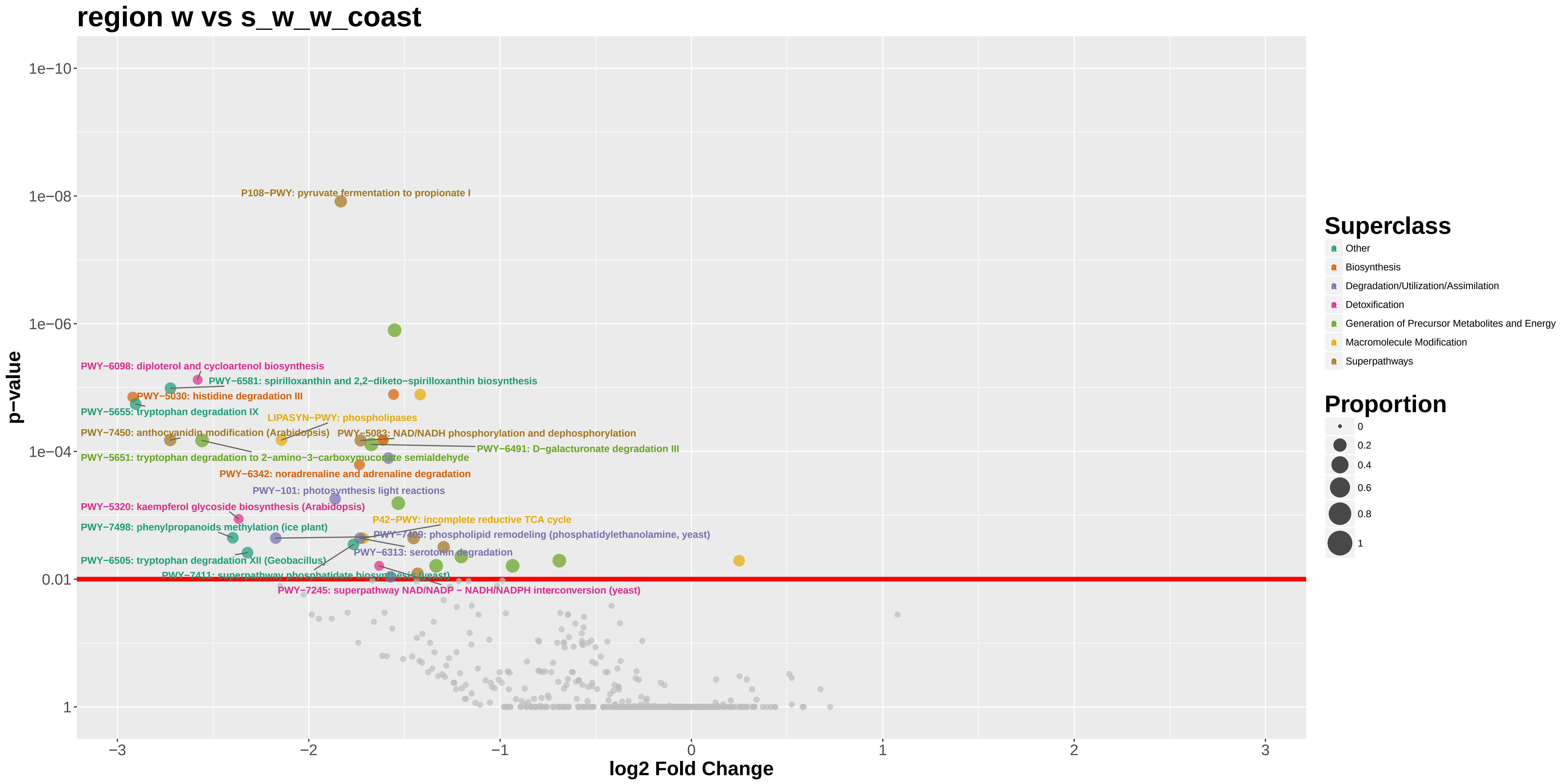
region w vs s_w_w_coast
p−value
log2 Fold Change
1e−10
1e−08
1e−06
1e−04
0.01
1
−3
−2
−1
0
1
2
3
P108−PWY: pyruvate fermentation to propionate I
PWY−6098: diploterol and cycloartenol biosynthesis
PWY−6581: spirilloxanthin and 2,2−diketo−spirilloxanthin biosynthesis
PWY−5030: histidine degradation III
PWY−5655: tryptophan degradation IX
LIPASYN−PWY: phospholipases
PWY−7450: anthocyanidin modification (Arabidopsis)
PWY−5083: NAD/NADH phosphorylation and dephosphorylation
PWY−6491: D−galacturonate degradation III
PWY−5651: tryptophan degradation to 2−amino−3−carboxymuconate semialdehyde
PWY−6342: noradrenaline and adrenaline degradation
PWY−101: photosynthesis light reactions
PWY−5320: kaempferol glycoside biosynthesis (Arabidopsis)
P42−PWY: incomplete reductive TCA cycle
PWY−7498: phenylpropanoids methylation (ice plant)
PWY−7409: phospholipid remodeling (phosphatidylethanolamine, yeast)
PWY−6313: serotonin degradation
PWY−6505: tryptophan degradation XII (Geobacillus)
PWY−7411: superpathway phosphatidate biosynthesis (yeast)
PWY−7245: superpathway NAD/NADP − NADH/NADPH interconversion (yeast)
Superclass
Other
Biosynthesis
Degradation/Utilization/Assimilation
Detoxification
Generation of Precursor Metabolites and Energy
Macromolecule Modification
Superpathways
Proportion
0
0.2
0.4
0.6
0.8
1

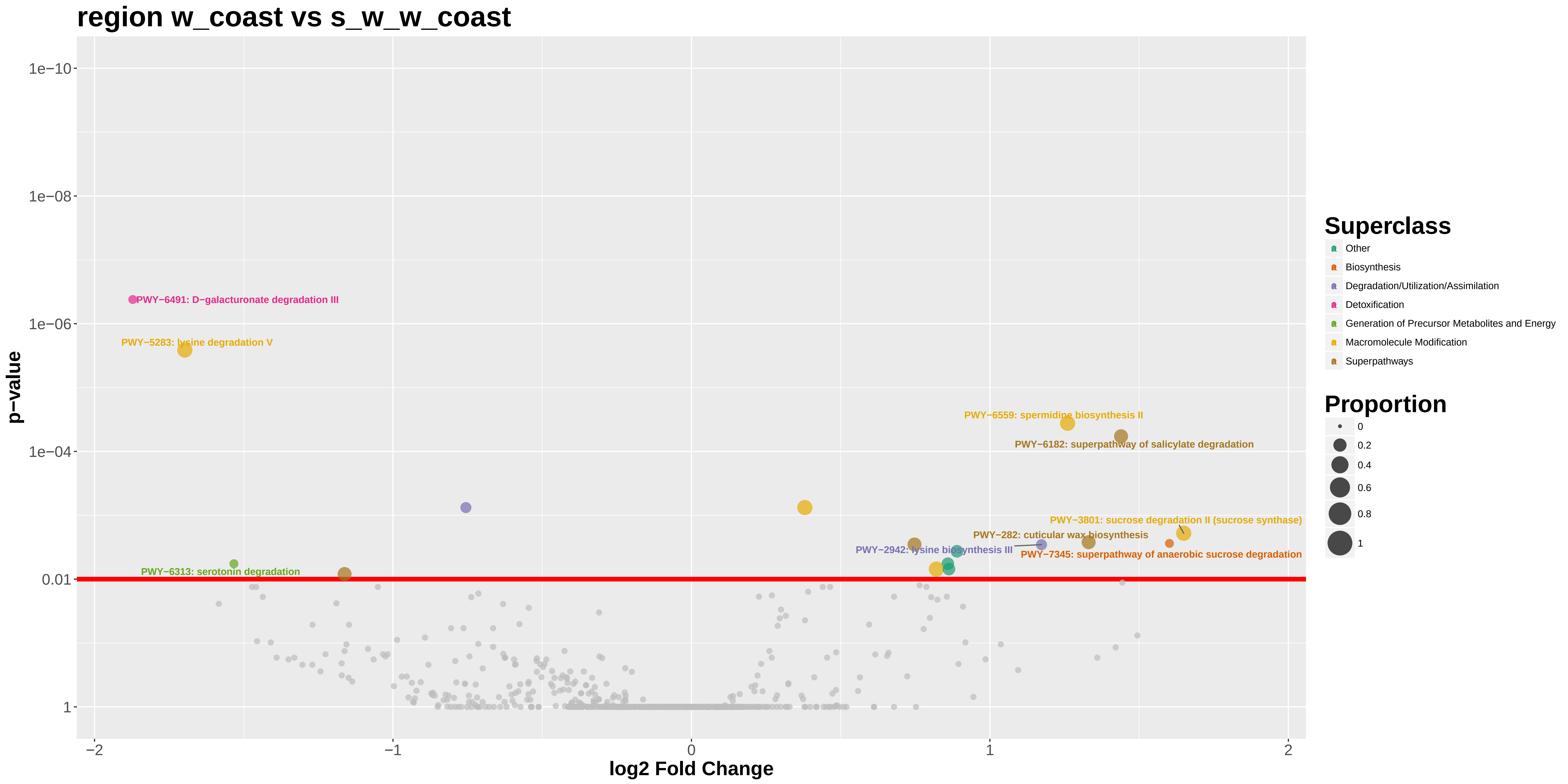

region w_coast vs s_w_w_coast
p−value
1e−10
1e−08
1e−06
1e−04
0.01
1
log2 Fold Change
−2
−1
0
1
2
PWY−6491: D−galacturonate degradation III
PWY−5283: lysine degradation V
PWY−6559: spermidine biosynthesis II
PWY−6182: superpathway of salicylate degradation
PWY−3801: sucrose degradation II (sucrose synthase)
PWY−282: cuticular wax biosynthesis
PWY−2942: lysine biosynthesis III
PWY−7345: superpathway of anaerobic sucrose degradation
PWY−6313: serotonin degradation
Superclass
Other
Biosynthesis
Degradation/Utilization/Assimilation
Detoxification
Generation of Precursor Metabolites and Energy
Macromolecule Modification
Superpathways
Proportion
0
0.2
0.4
0.6
0.8
1

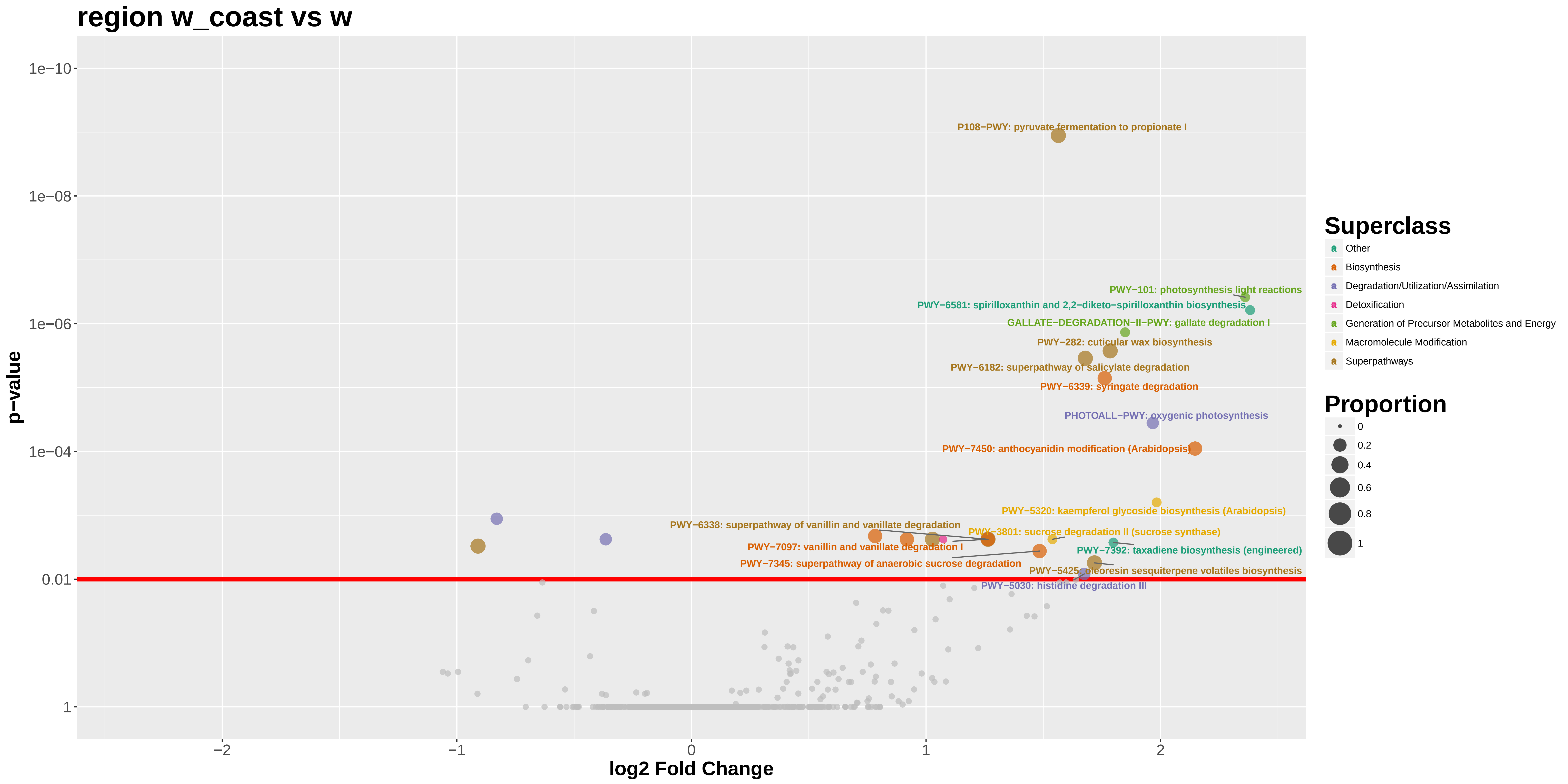
region w_coast vs w
P108−PWY: pyruvate fermentation to propionate I
PWY−101: photosynthesis light reactions
PWY−6581: spirilloxanthin and 2,2−diketo−spirilloxanthin biosynthesis
GALLATE−DEGRADATION−II−PWY: gallate degradation I
PWY−282: cuticular wax biosynthesis
PWY−6182: superpathway of salicylate degradation
PWY−6339: syringate degradation
PHOTOALL−PWY: oxygenic photosynthesis
PWY−7450: anthocyanidin modification (Arabidopsis)
PWY−5320: kaempferol glycoside biosynthesis (Arabidopsis)
PWY−6338: superpathway of vanillin and vanillate degradation
PWY−3801: sucrose degradation II (sucrose synthase)
PWY−7097: vanillin and vanillate degradation I
PWY−7392: taxadiene biosynthesis (engineered)
PWY−7345: superpathway of anaerobic sucrose degradation
PWY−5425: oleoresin sesquiterpene volatiles biosynthesis
PWY−5030: histidine degradation III
p−value
1e−10
1e−08
1e−06
1e−04
0.01
1
−2
−1
0
1
2
log2 Fold Change
Superclass
Other
Biosynthesis
Degradation/Utilization/Assimilation
Detoxification
Generation of Precursor Metabolites and Energy
Macromolecule Modification
Superpathways
Proportion
0
0.2
0.4
0.6
0.8
1

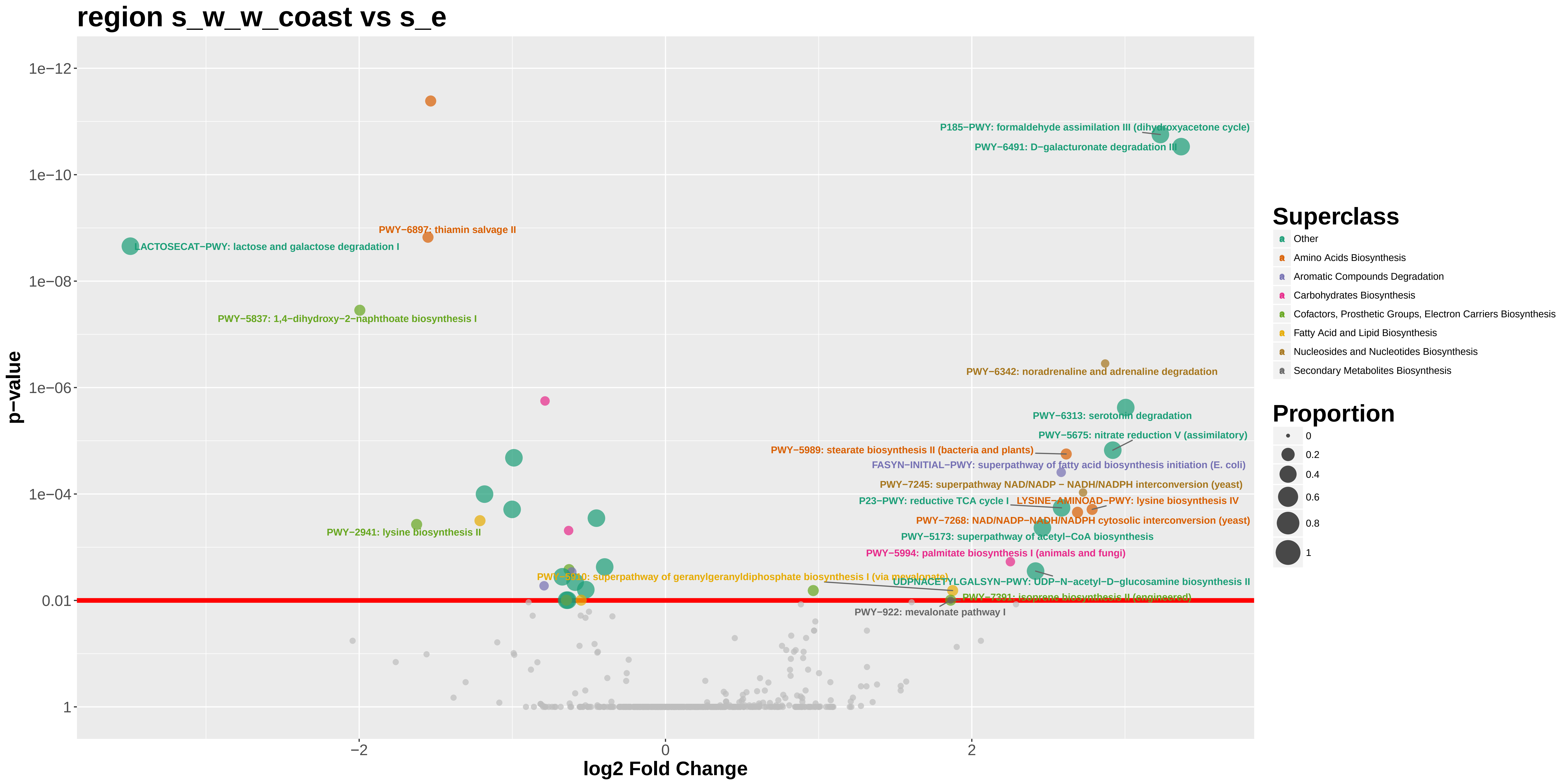

region s_w_w_coast vs s_e
p−value
log2 Fold Change
1e−12
1e−10
1e−08
1e−06
1e−04
0.01
1
−2
0
2
P185−PWY: formaldehyde assimilation III (dihydroxyacetone cycle)
PWY−6491: D−galacturonate degradation III
PWY−6897: thiamin salvage II
LACTOSECAT−PWY: lactose and galactose degradation I
PWY−5837: 1,4−dihydroxy−2−naphthoate biosynthesis I
PWY−6342: noradrenaline and adrenaline degradation
PWY−6313: serotonin degradation
PWY−5675: nitrate reduction V (assimilatory)
PWY−5989: stearate biosynthesis II (bacteria and plants)
FASYN−INITIAL−PWY: superpathway of fatty acid biosynthesis initiation (E. coli)
PWY−7245: superpathway NAD/NADP − NADH/NADPH interconversion (yeast)
P23−PWY: reductive TCA cycle I
LYSINE−AMINOAD−PWY: lysine biosynthesis IV
PWY−7268: NAD/NADP−NADH/NADPH cytosolic interconversion (yeast)
PWY−2941: lysine biosynthesis II
PWY−5173: superpathway of acetyl−CoA biosynthesis
PWY−5994: palmitate biosynthesis I (animals and fungi)
PWY−5910: superpathway of geranylgeranyldiphosphate biosynthesis I (via mevalonate)
UDPNACETYLGALSYN−PWY: UDP−N−acetyl−D−glucosamine biosynthesis II
PWY−7391: isoprene biosynthesis II (engineered)
PWY−922: mevalonate pathway I
Superclass
Other
Amino Acids Biosynthesis
Aromatic Compounds Degradation
Carbohydrates Biosynthesis
Cofactors, Prosthetic Groups, Electron Carriers Biosynthesis
Fatty Acid and Lipid Biosynthesis
Nucleosides and Nucleotides Biosynthesis
Secondary Metabolites Biosynthesis
Proportion
0
0.2
0.4
0.6
0.8
1

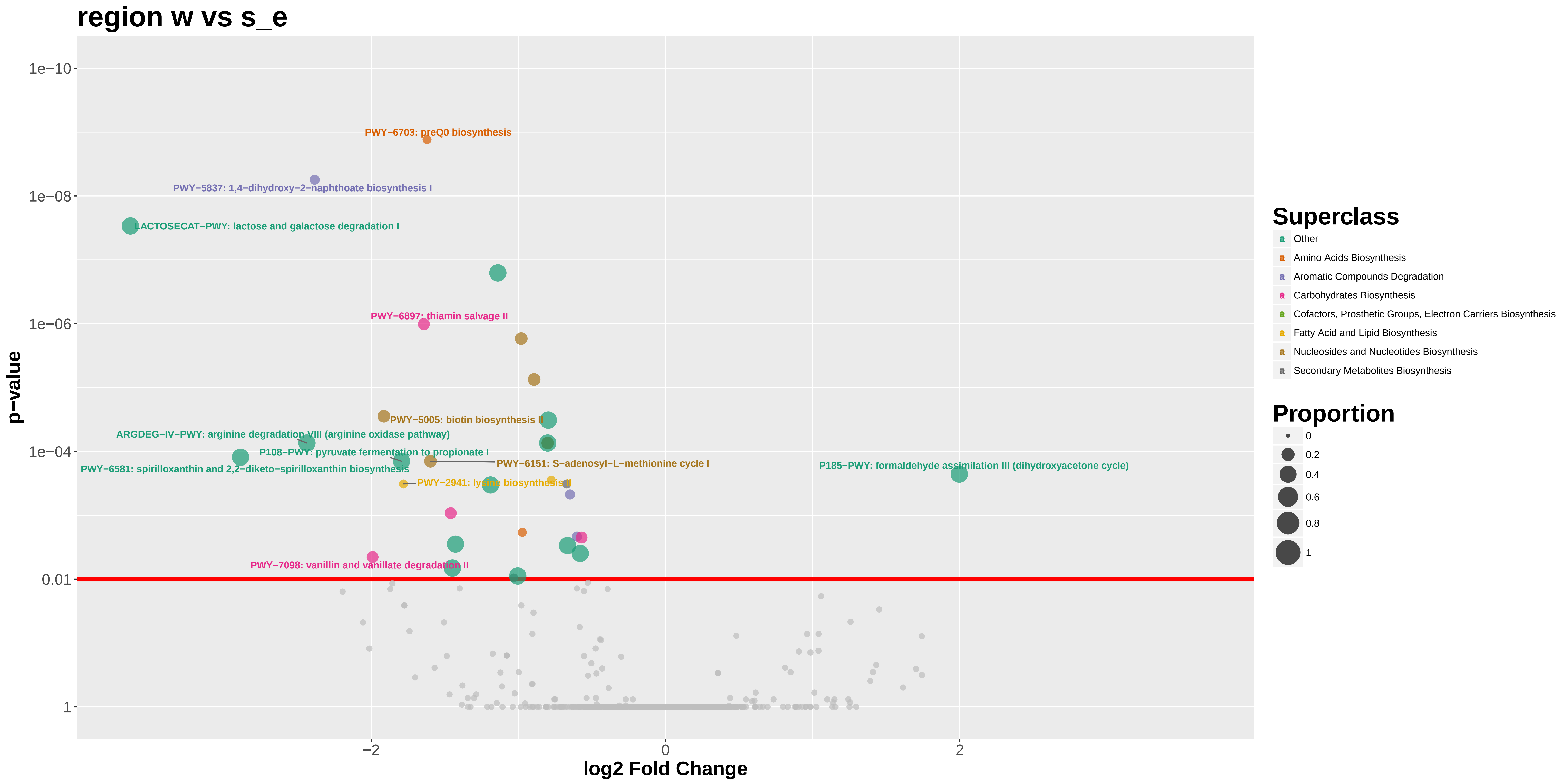
region w vs s_e
p−value
1e−10
1e−08
1e−06
1e−04
0.01
1
log2 Fold Change
−2
0
2
PWY−6703: preQ0 biosynthesis
PWY−5837: 1,4−dihydroxy−2−naphthoate biosynthesis I
LACTOSECAT−PWY: lactose and galactose degradation I
PWY−6897: thiamin salvage II
PWY−5005: biotin biosynthesis II
ARGDEG−IV−PWY: arginine degradation VIII (arginine oxidase pathway)
P108−PWY: pyruvate fermentation to propionate I
PWY−6151: S−adenosyl−L−methionine cycle I
PWY−6581: spirilloxanthin and 2,2'−diketo−spirilloxanthin biosynthesis
P185−PWY: formaldehyde assimilation III (dihydroxyacetone cycle)
PWY−2941: lysine biosynthesis II
PWY−7098: vanillin and vanillate degradation II
Superclass
Other
Amino Acids Biosynthesis
Aromatic Compounds Degradation
Carbohydrates Biosynthesis
Cofactors, Prosthetic Groups, Electron Carriers Biosynthesis
Fatty Acid and Lipid Biosynthesis
Nucleosides and Nucleotides Biosynthesis
Secondary Metabolites Biosynthesis
Proportion
0
0.2
0.4
0.6
0.8
1

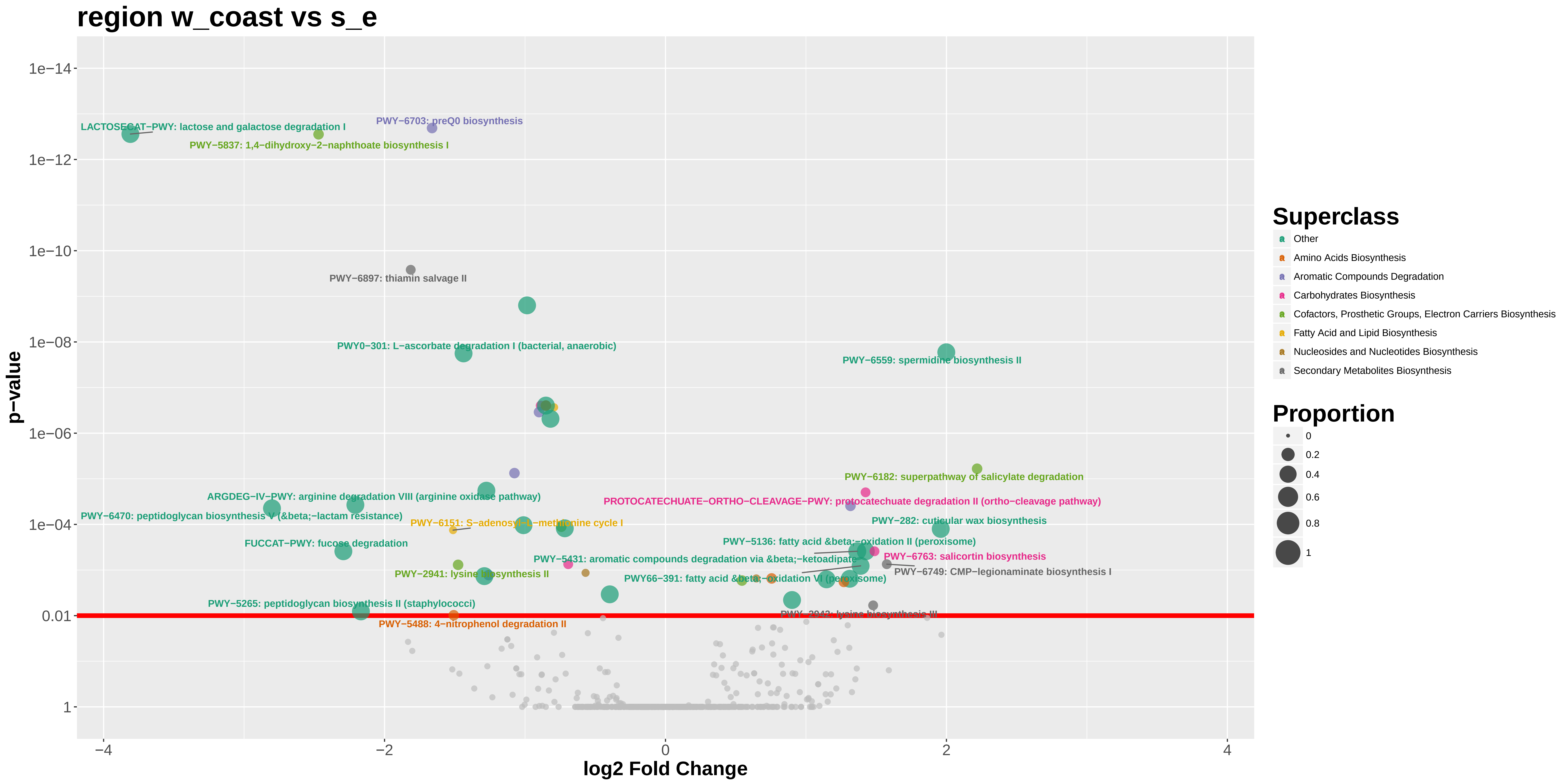
region w_coast vs s_e
p−value
log2 Fold Change
1e−14
1e−12
1e−10
1e−08
1e−06
1e−04
0.01
1
−4
−2
0
2
4
LACTOSECAT−PWY: lactose and galactose degradation I
PWY−6703: preQ0 biosynthesis
PWY−5837: 1,4−dihydroxy−2−naphthoate biosynthesis I
PWY−6897: thiamin salvage II
PWY0−301: L−ascorbate degradation I (bacterial, anaerobic)
PWY−6559: spermidine biosynthesis II
PWY−6182: superpathway of salicylate degradation
ARGDEG−IV−PWY: arginine degradation VIII (arginine oxidase pathway)
PROTOCATECHUATE−ORTHO−CLEAVAGE−PWY: protocatechuate degradation II (ortho−cleavage pathway)
PWY−6470: peptidoglycan biosynthesis V (β−lactam resistance)
PWY−6151: S−adenosyl−L−methionine cycle I
PWY−282: cuticular wax biosynthesis
FUCCAT−PWY: fucose degradation
PWY−5136: fatty acid β−oxidation II (peroxisome)
PWY−5431: aromatic compounds degradation via β−ketoadipate
PWY−6763: salicortin biosynthesis
PWY−2941: lysine biosynthesis II
PWY66−391: fatty acid β−oxidation VI (peroxisome)
PWY−6749: CMP−legionaminate biosynthesis I
PWY−5265: peptidoglycan biosynthesis II (staphylococci)
PWY−2942: lysine biosynthesis III
PWY−5488: 4−nitrophenol degradation II
Superclass
Other
Amino Acids Biosynthesis
Aromatic Compounds Degradation
Carbohydrates Biosynthesis
Cofactors, Prosthetic Groups, Electron Carriers Biosynthesis
Fatty Acid and Lipid Biosynthesis
Nucleosides and Nucleotides Biosynthesis
Secondary Metabolites Biosynthesis
Proportion
0
0.2
0.4
0.6
0.8
1

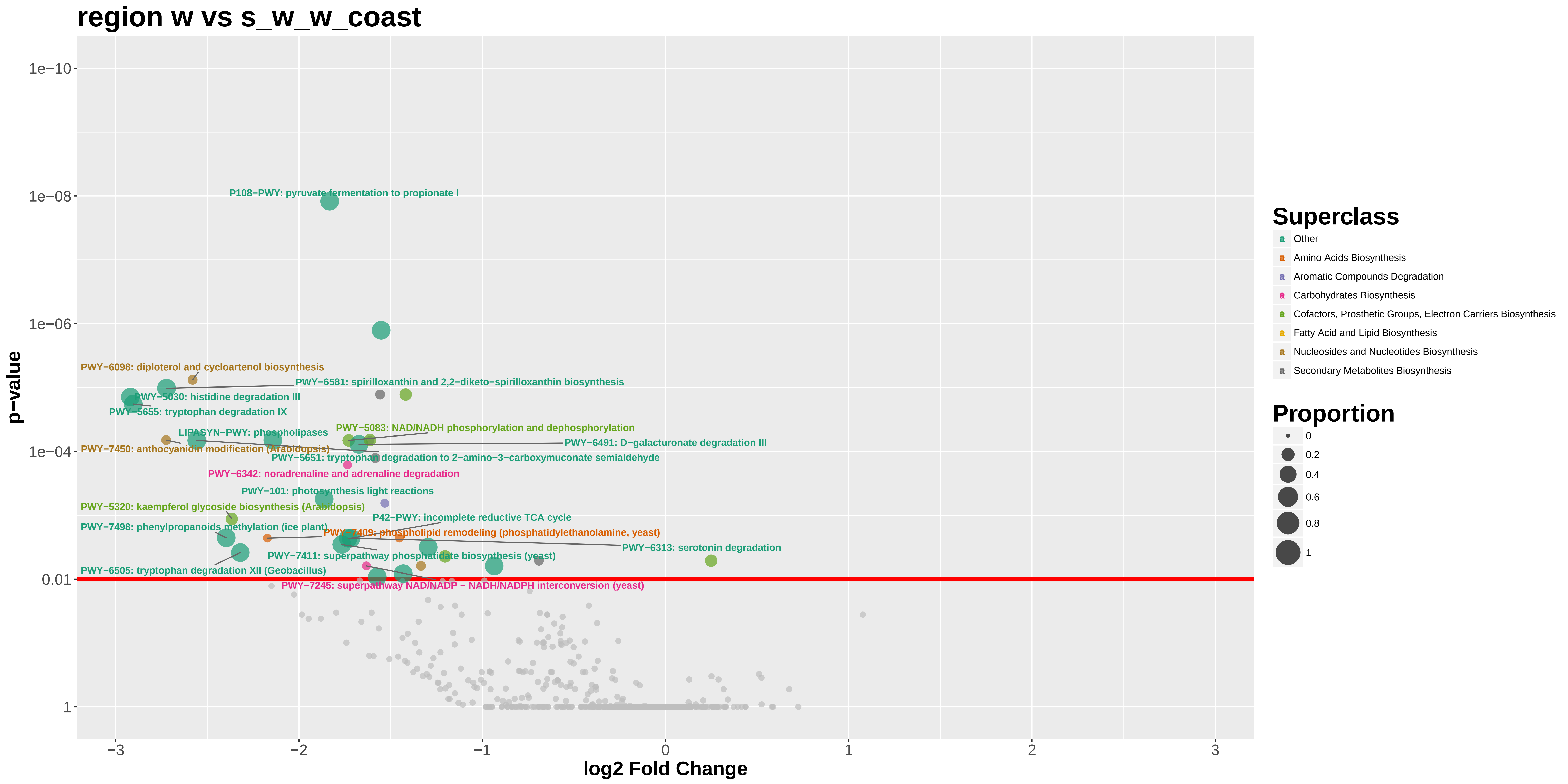

region w vs s_w_w_coast
p−value
log2 Fold Change
1e−10
1e−08
1e−06
1e−04
0.01
1
−3
−2
−1
0
1
2
3
P108−PWY: pyruvate fermentation to propionate I
PWY−6098: diploterol and cycloartenol biosynthesis
PWY−6581: spirilloxanthin and 2,2−diketo−spirilloxanthin biosynthesis
PWY−5030: histidine degradation III
PWY−5655: tryptophan degradation IX
PWY−5083: NAD/NADH phosphorylation and dephosphorylation
LIPASYN−PWY: phospholipases
PWY−6491: D−galacturonate degradation III
PWY−7450: anthocyanidin modification (Arabidopsis)
PWY−5651: tryptophan degradation to 2−amino−3−carboxymuconate semialdehyde
PWY−6342: noradrenaline and adrenaline degradation
PWY−101: photosynthesis light reactions
PWY−5320: kaempferol glycoside biosynthesis (Arabidopsis)
P42−PWY: incomplete reductive TCA cycle
PWY−7498: phenylpropanoids methylation (ice plant)
PWY−7409: phospholipid remodeling (phosphatidylethanolamine, yeast)
PWY−6313: serotonin degradation
PWY−7411: superpathway phosphatidate biosynthesis (yeast)
PWY−6505: tryptophan degradation XII (Geobacillus)
PWY−7245: superpathway NAD/NADP − NADH/NADPH interconversion (yeast)
Superclass
Other
Amino Acids Biosynthesis
Aromatic Compounds Degradation
Carbohydrates Biosynthesis
Cofactors, Prosthetic Groups, Electron Carriers Biosynthesis
Fatty Acid and Lipid Biosynthesis
Nucleosides and Nucleotides Biosynthesis
Secondary Metabolites Biosynthesis
Proportion
0
0.2
0.4
0.6
0.8
1

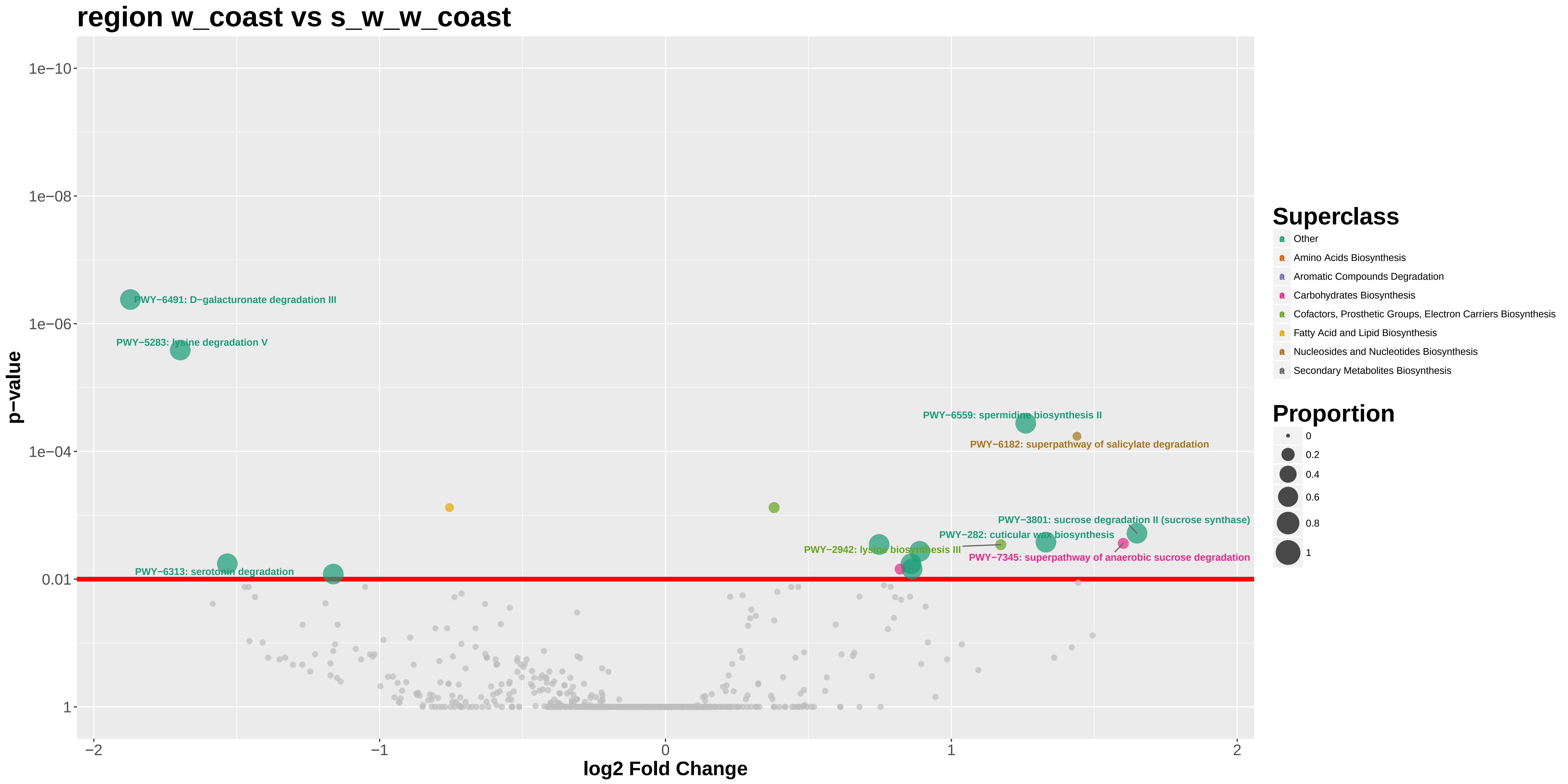

region w_coast vs s_w_w_coast
p−value
log2 Fold Change
1e−10
1e−08
1e−06
1e−04
0.01
1
−2
−1
0
1
2
PWY−6491: D−galacturonate degradation III
PWY−5283: lysine degradation V
PWY−6313: serotonin degradation
PWY−6559: spermidine biosynthesis II
PWY−6182: superpathway of salicylate degradation
PWY−3801: sucrose degradation II (sucrose synthase)
PWY−282: cuticular wax biosynthesis
PWY−2942: lysine biosynthesis III
PWY−7345: superpathway of anaerobic sucrose degradation
Superclass
Other
Amino Acids Biosynthesis
Aromatic Compounds Degradation
Carbohydrates Biosynthesis
Cofactors, Prosthetic Groups, Electron Carriers Biosynthesis
Fatty Acid and Lipid Biosynthesis
Nucleosides and Nucleotides Biosynthesis
Secondary Metabolites Biosynthesis
Proportion
0
0.2
0.4
0.6
0.8
1

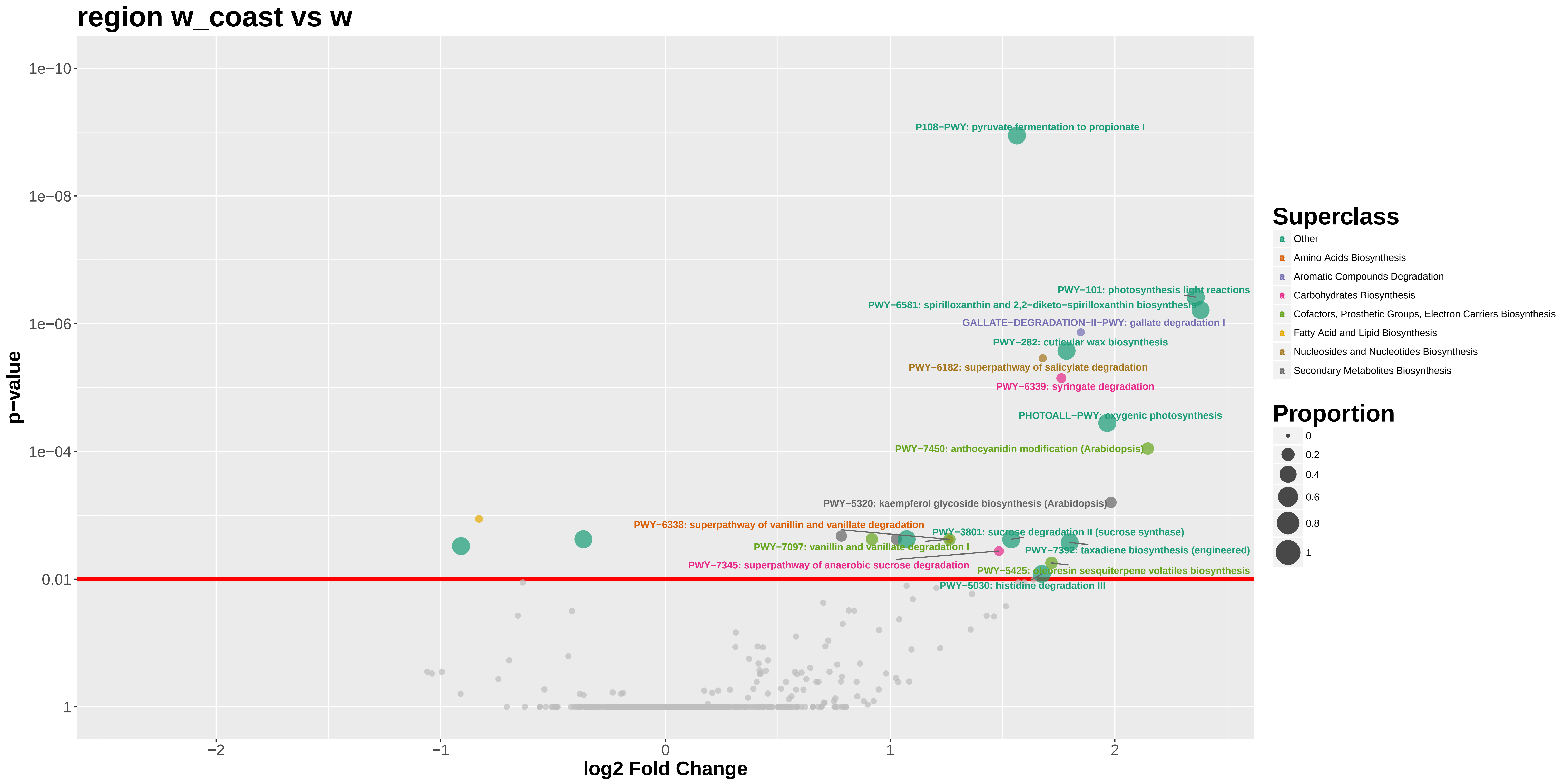
region w_coast vs w
P108−PWY: pyruvate fermentation to propionate I
PWY−101: photosynthesis light reactions
PWY−6581: spirilloxanthin and 2,2−diketo−spirilloxanthin biosynthesis
GALLATE−DEGRADATION−II−PWY: gallate degradation I
PWY−282: cuticular wax biosynthesis
PWY−6182: superpathway of salicylate degradation
PWY−6339: syringate degradation
PHOTOALL−PWY: oxygenic photosynthesis
PWY−7450: anthocyanidin modification (Arabidopsis)
PWY−5320: kaempferol glycoside biosynthesis (Arabidopsis)
PWY−6338: superpathway of vanillin and vanillate degradation
PWY−3801: sucrose degradation II (sucrose synthase)
PWY−7097: vanillin and vanillate degradation I
PWY−7392: taxadiene biosynthesis (engineered)
PWY−7345: superpathway of anaerobic sucrose degradation
PWY−5425: oleoresin sesquiterpene volatiles biosynthesis
PWY−5030: histidine degradation III
p−value
1e−10
1e−08
1e−06
1e−04
0.01
1
log2 Fold Change
−2
−1
0
1
2
Superclass
Other
Amino Acids Biosynthesis
Aromatic Compounds Degradation
Carbohydrates Biosynthesis
Cofactors, Prosthetic Groups, Electron Carriers Biosynthesis
Fatty Acid and Lipid Biosynthesis
Nucleosides and Nucleotides Biosynthesis
Secondary Metabolites Biosynthesis
Proportion
0
0.2
0.4
0.6
0.8
1
