# Supplementary material for: Metagenomic characterization of ambulances across the USA
Source: Microbiome. 2017 Sep 22;5:125. doi: 10.1186/s40168-017-0339-6 (PMC5610413; doi:10.1186/s40168-017-0339-6)
Supplement: Supplementary file 29 — Figure S12. Volcano plot of the p-value versus log2-fold change (LFC) of HUMAnN2 pathway abundances resulting from a DESeq2 differential abundance analysis for city class with FDR correction (Benjamini-Hochberg correction, α = 0.01). Class combinations were selected based on overlap data classification performance. Points vary in color based on pathway superclass and size based on the proportion of genes in that class with p < α. Genes in the 95th percentile of absolute LFC are labeled. (PDF 338 kb) [file 40168_2017_339_MOESM29_ESM.pdf]

city S003 vs S005

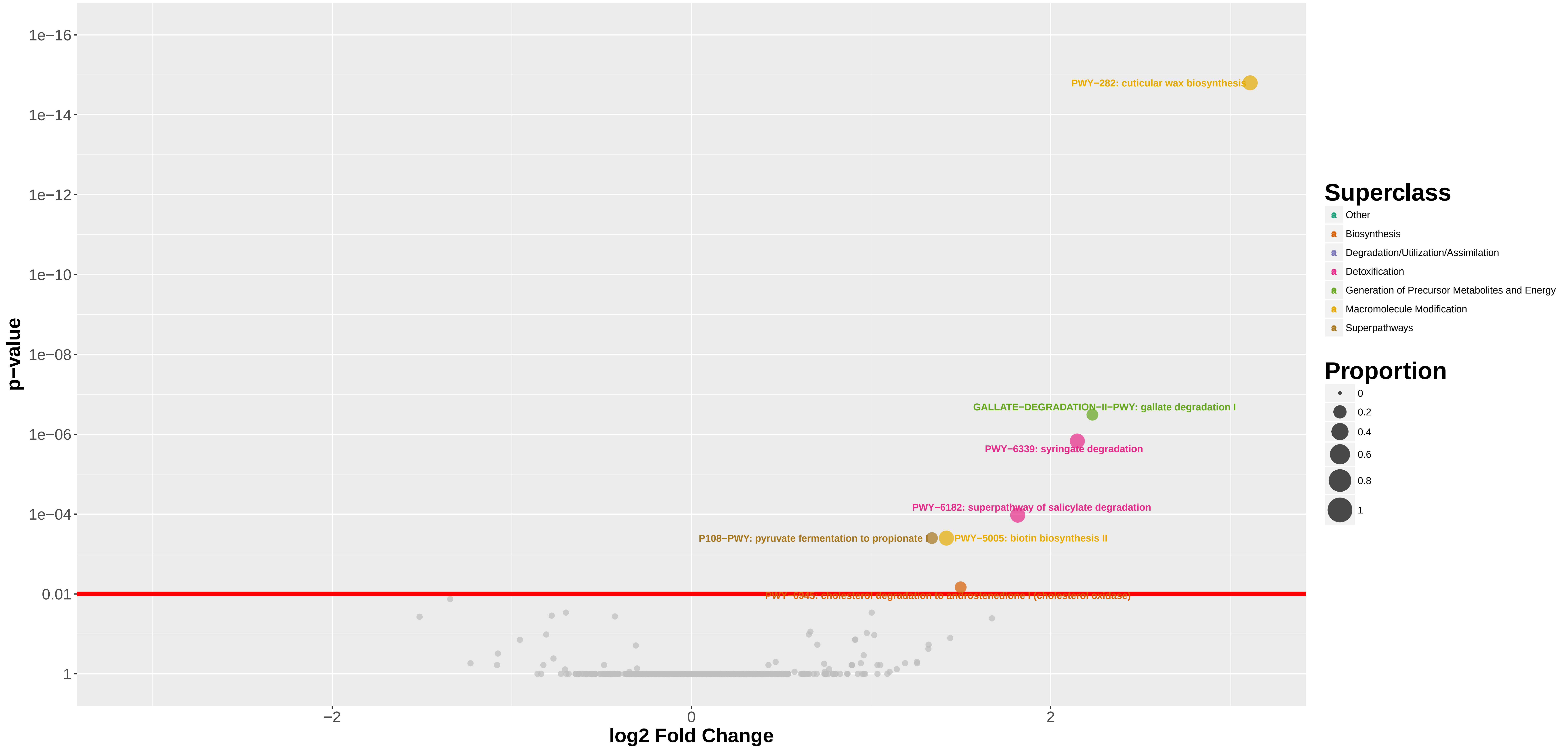

city S002 vs S005

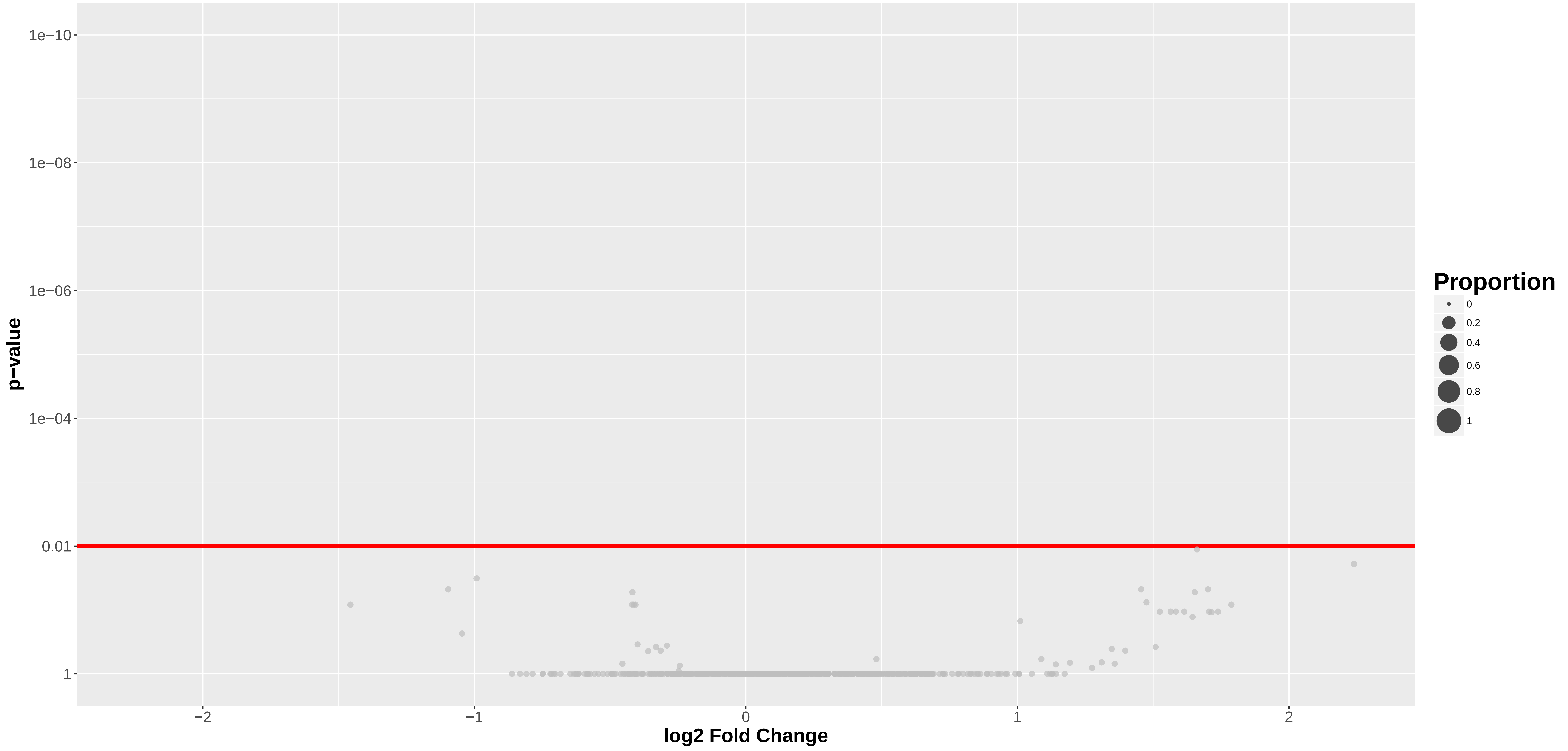

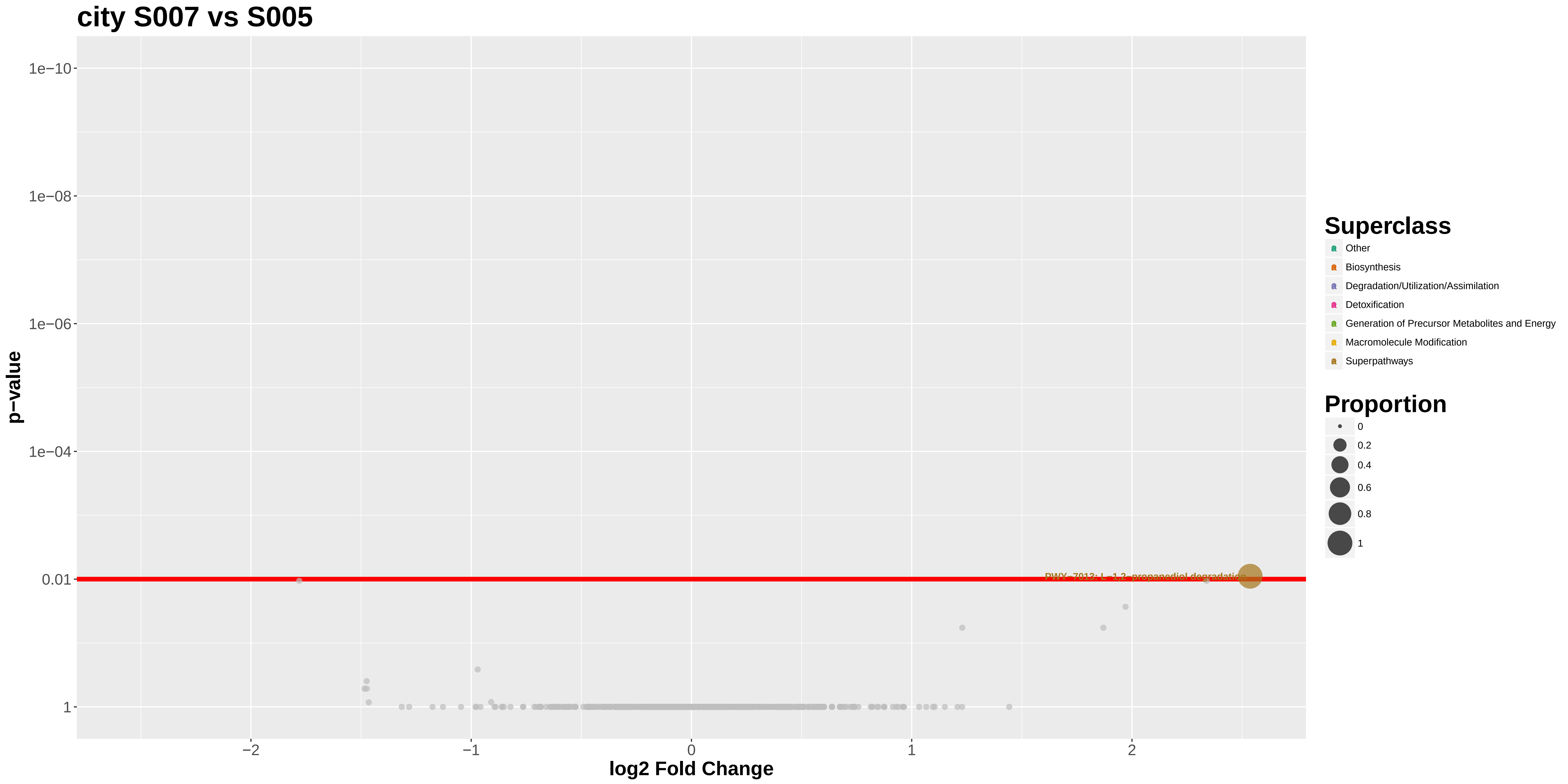

city S007 vs S005
p−value
1e−10
1e−08
1e−06
1e−04
0.01
1
−2
−1
0
1
2
log2 Fold Change
PWY−7013: L−1,2−propanediol degradation
Superclass
Other
Biosynthesis
Degradation/Utilization/Assimilation
Detoxification
Generation of Precursor Metabolites and Energy
Macromolecule Modification
Superpathways
Proportion
0
0.2
0.4
0.6
0.8
1

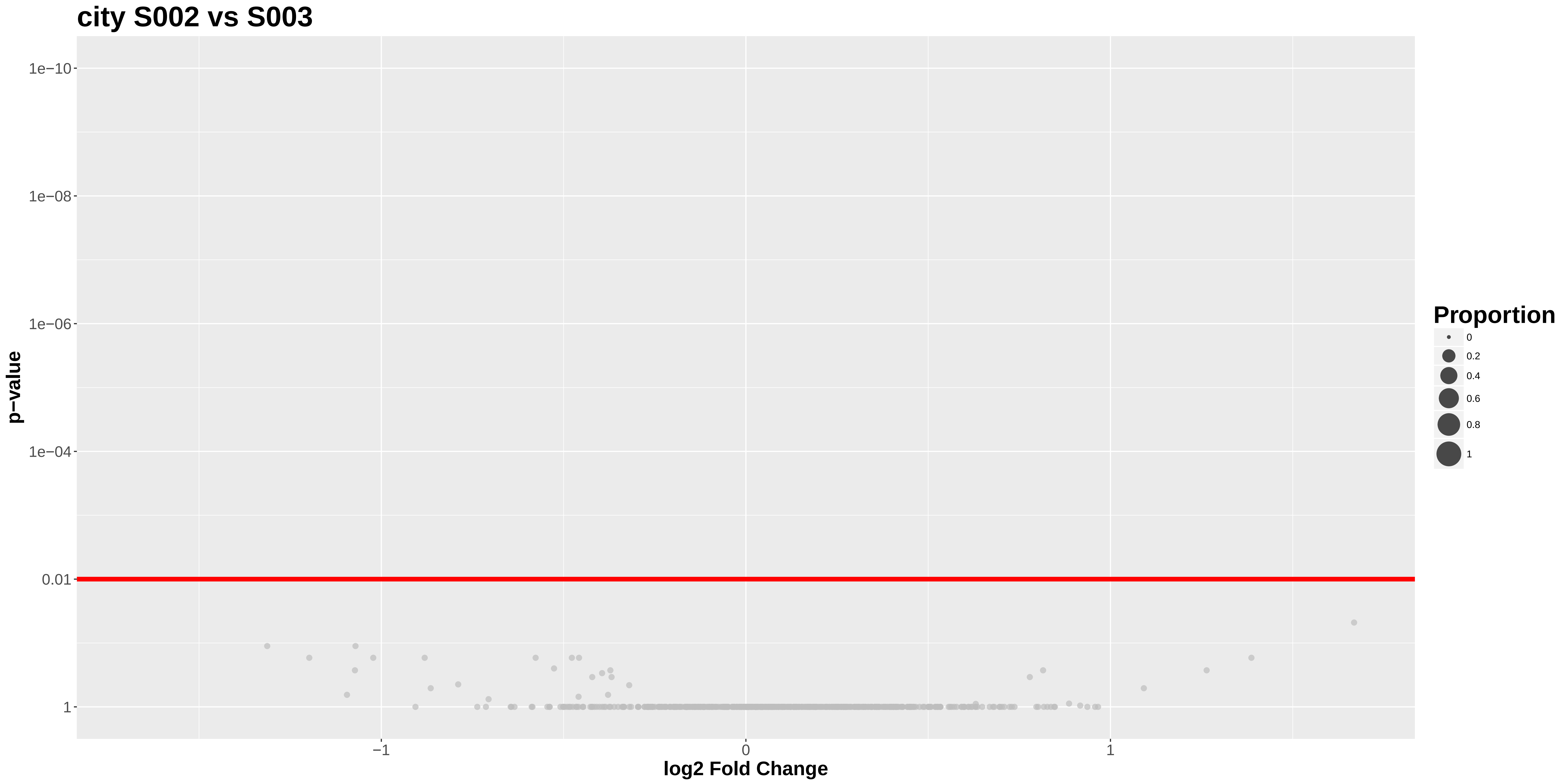

city S002 vs S003
1e−10
1e−08
1e−06
1e−04
0.01
1
p−value
−1
0
1
log2 Fold Change
Proportion
0
0.2
0.4
0.6
0.8
1

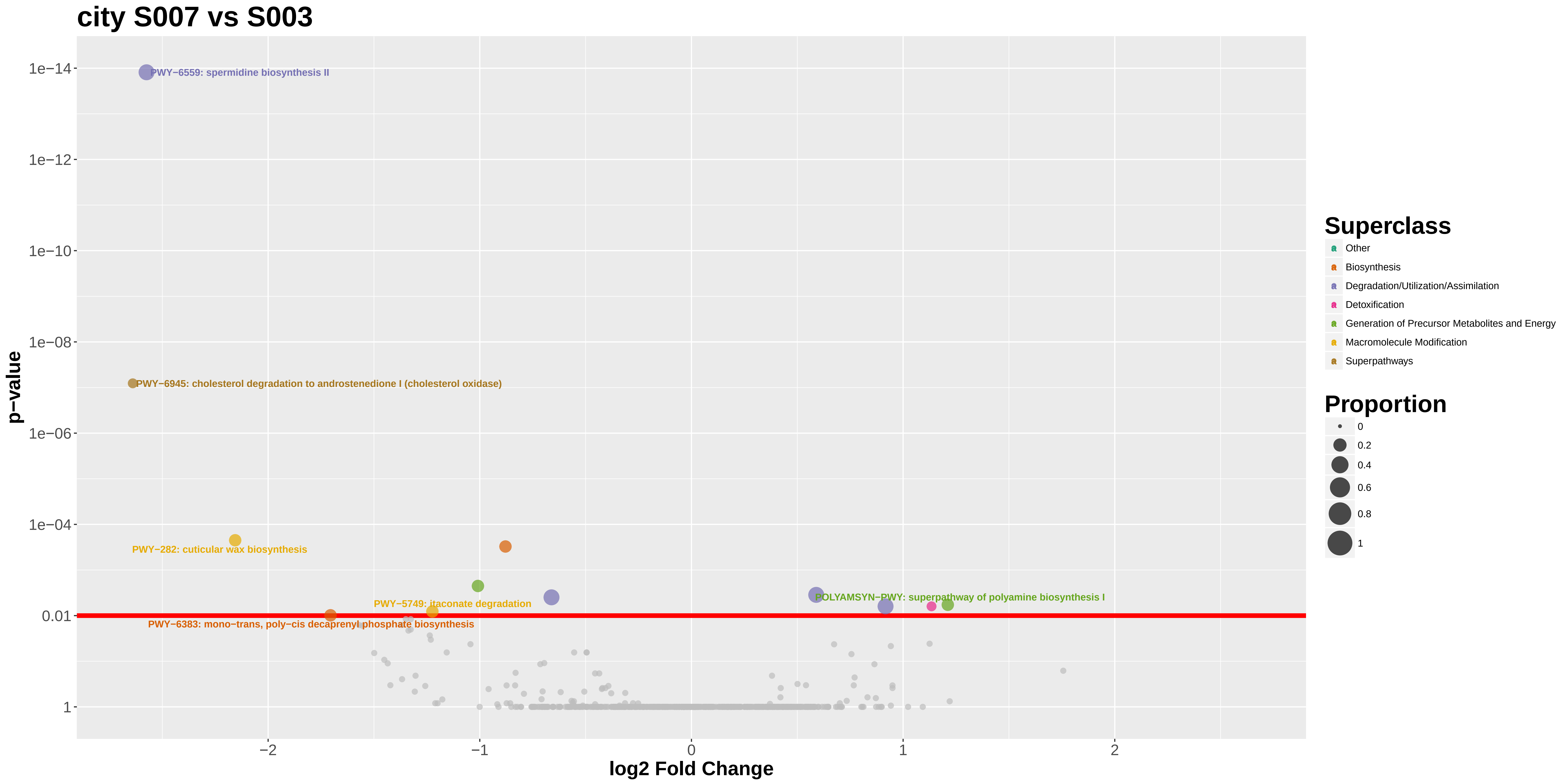
city S007 vs S003
PWY−6559: spermidine biosynthesis II
PWY−6945: cholesterol degradation to androstenedione I (cholesterol oxidase)
PWY−282: cuticular wax biosynthesis
PWY−5749: itaconate degradation
PWY−6383: mono−trans, poly−cis decaprenyl phosphate biosynthesis
POLYAMSYN−PWY: superpathway of polyamine biosynthesis I
1e−14
1e−12
1e−10
1e−08
1e−06
1e−04
0.01
1
p−value
−2
−1
0
1
2
log2 Fold Change
Superclass
Other
Biosynthesis
Degradation/Utilization/Assimilation
Detoxification
Generation of Precursor Metabolites and Energy
Macromolecule Modification
Superpathways
Proportion
0
0.2
0.4
0.6
0.8
1

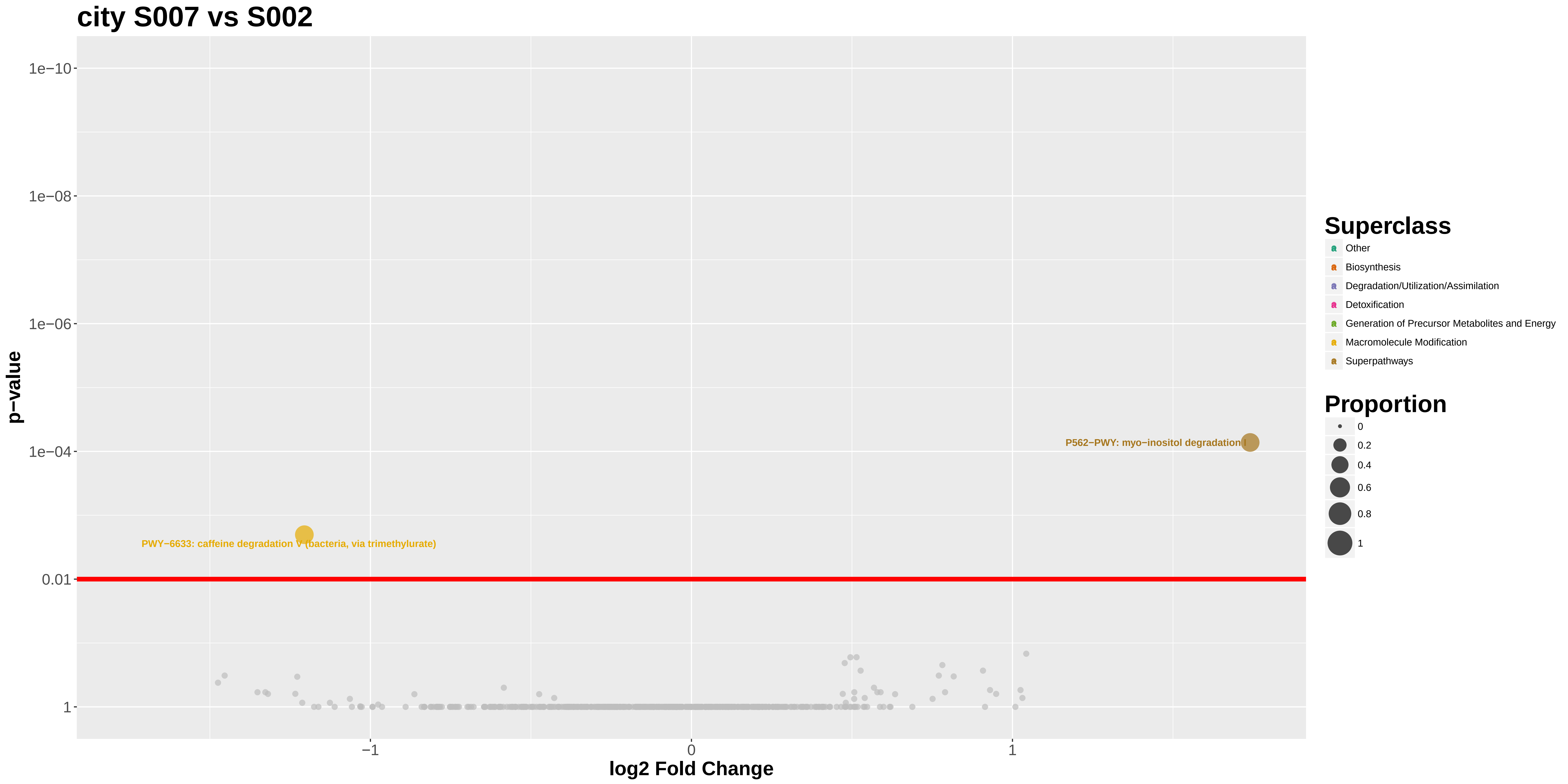
city S007 vs S002
p−value
1e−10
1e−08
1e−06
1e−04
0.01
1
log2 Fold Change
−1
0
1
PWY−6633: caffeine degradation V (bacteria, via trimethylurate)
P562−PWY: myo−inositol degradation I
Superclass
Other
Biosynthesis
Degradation/Utilization/Assimilation
Detoxification
Generation of Precursor Metabolites and Energy
Macromolecule Modification
Superpathways
Proportion
0
0.2
0.4
0.6
0.8
1

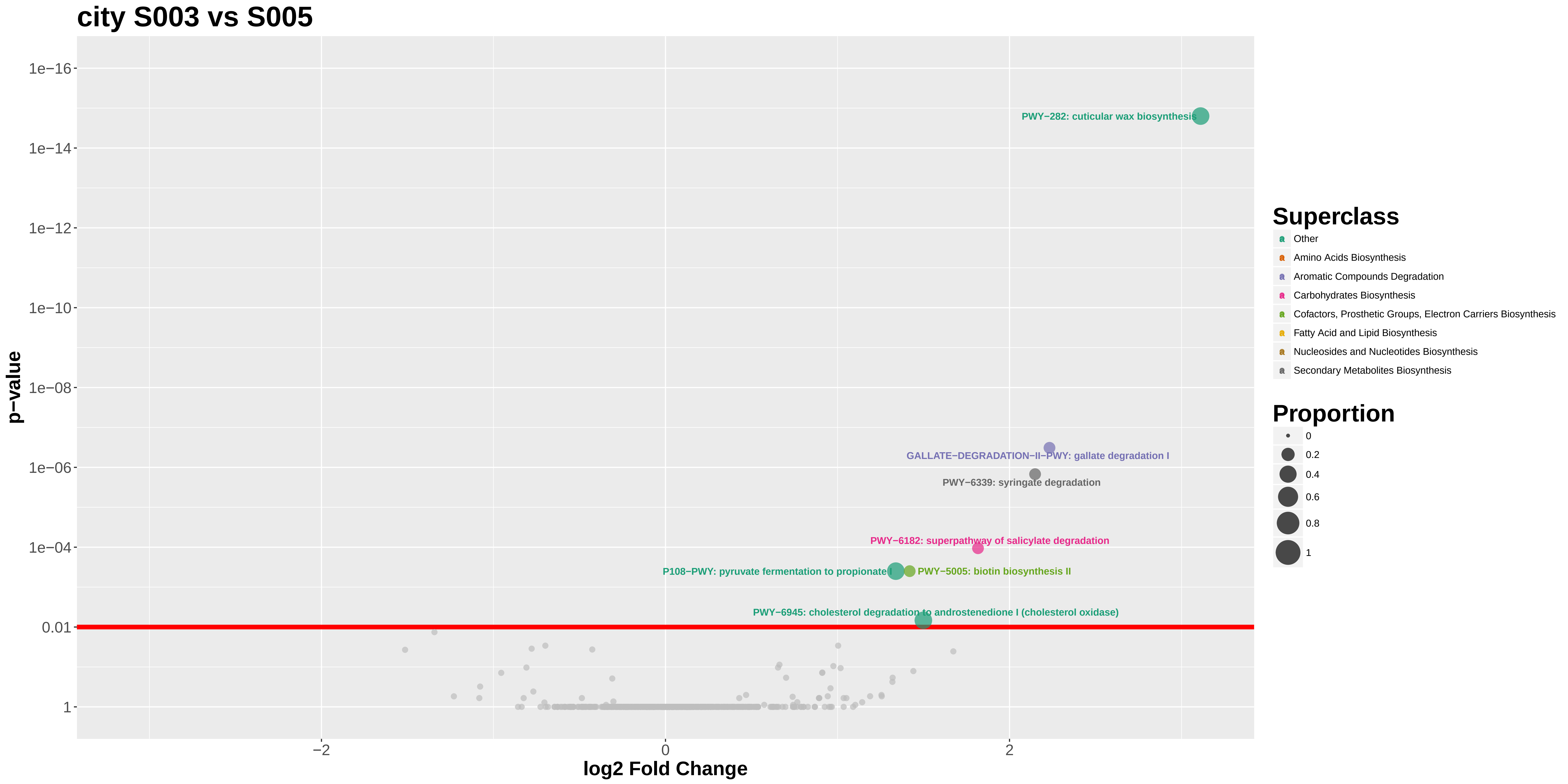

city S003 vs S005
p−value
1e−16
1e−14
1e−12
1e−10
1e−08
1e−06
1e−04
0.01
1
log2 Fold Change
−2
0
2
PWY−282: cuticular wax biosynthesis
GALLATE−DEGRADATION−II−PWY: gallate degradation I
PWY−6339: syringate degradation
PWY−6182: superpathway of salicylate degradation
P108−PWY: pyruvate fermentation to propionate I
PWY−5005: biotin biosynthesis II
PWY−6945: cholesterol degradation to androstenedione I (cholesterol oxidase)
Superclass
Other
Amino Acids Biosynthesis
Aromatic Compounds Degradation
Carbohydrates Biosynthesis
Cofactors, Prosthetic Groups, Electron Carriers Biosynthesis
Fatty Acid and Lipid Biosynthesis
Nucleosides and Nucleotides Biosynthesis
Secondary Metabolites Biosynthesis
Proportion
0
0.2
0.4
0.6
0.8
1

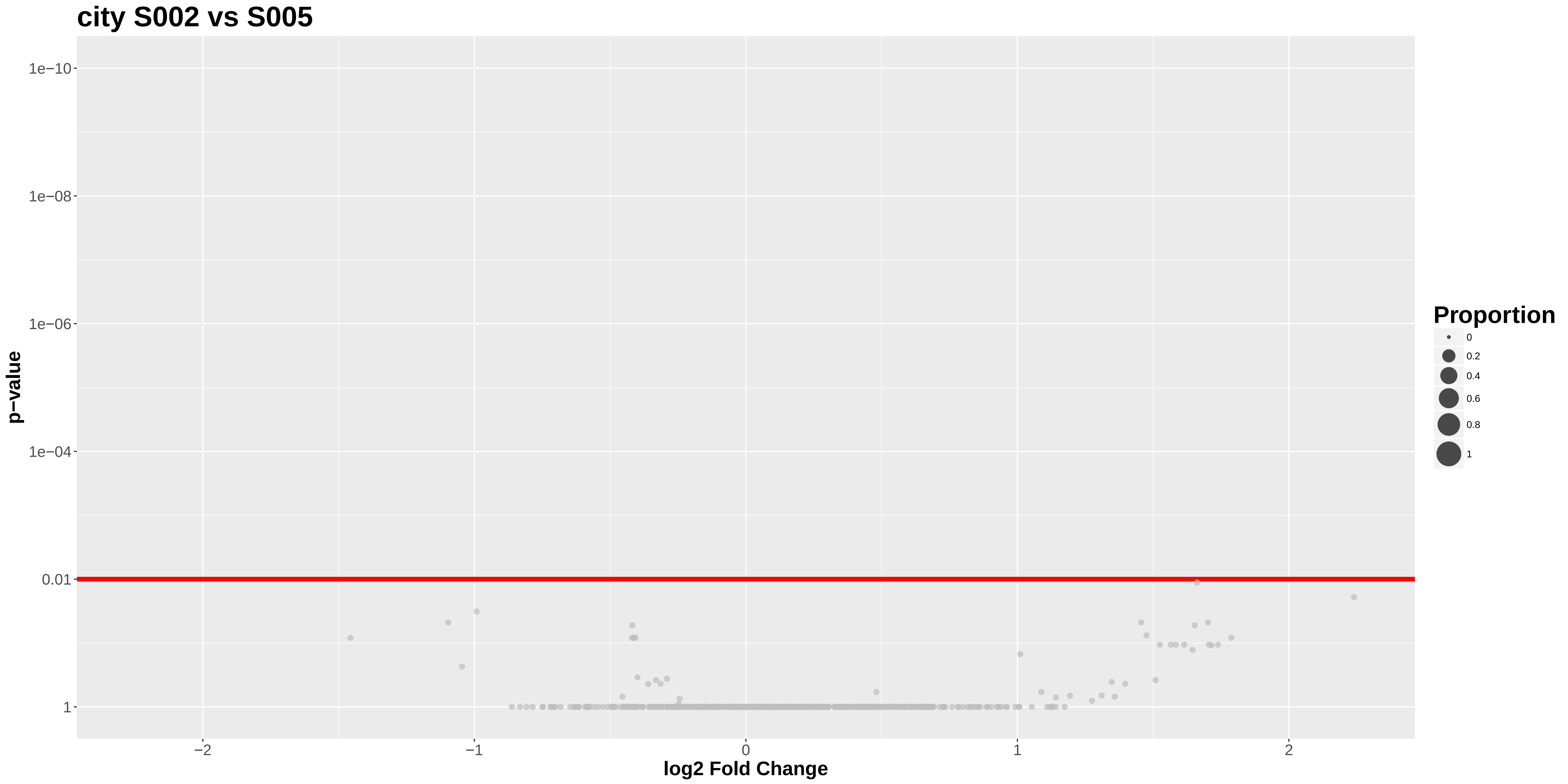

city S002 vs S005
p−value
1e−10
1e−08
1e−06
1e−04
0.01
1
−2
−1
0
1
2
log2 Fold Change
Proportion
0
0.2
0.4
0.6
0.8
1

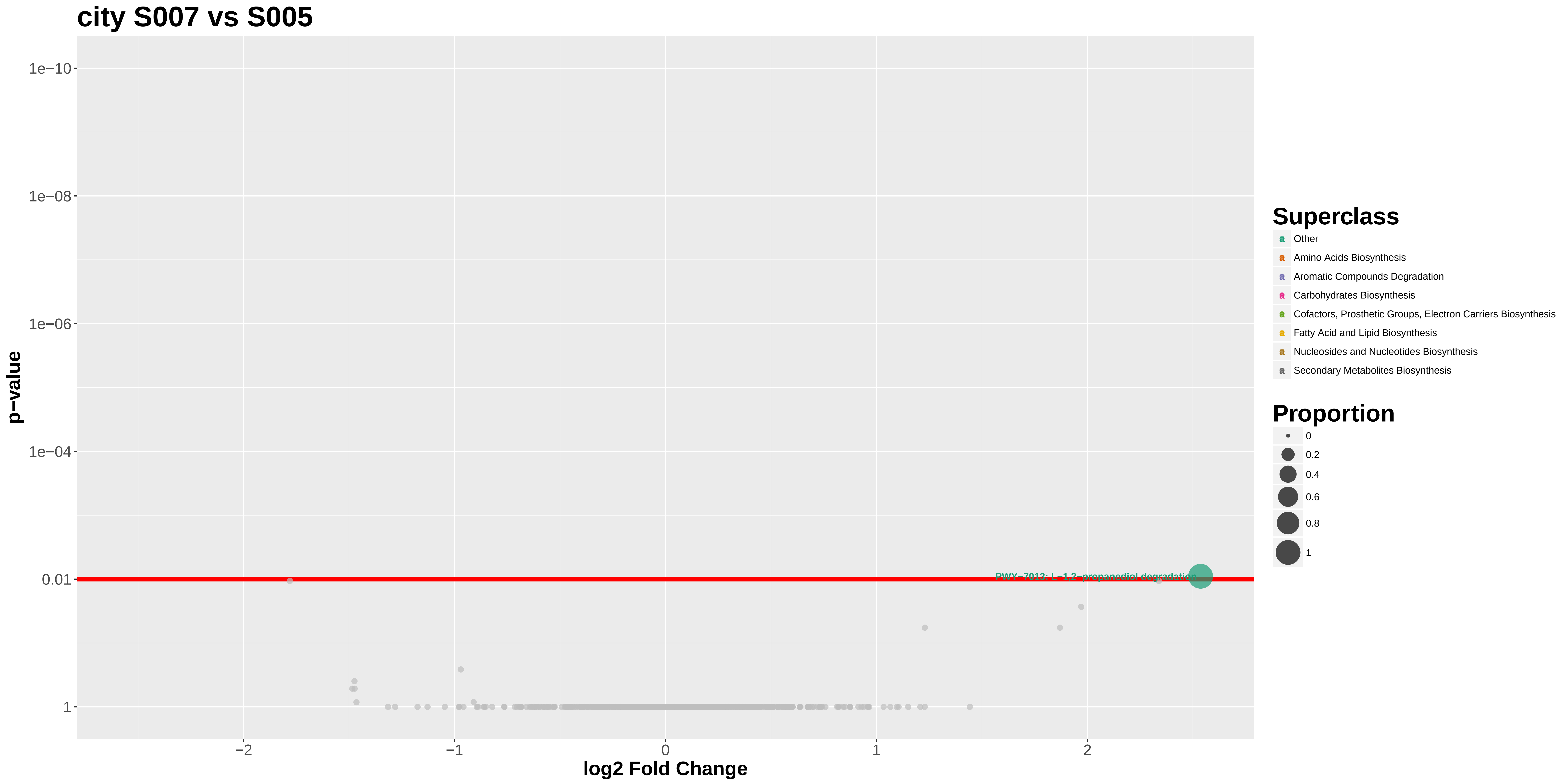

city S007 vs S005
p−value
1e−10
1e−08
1e−06
1e−04
0.01
1
−2
−1
0
1
2
log2 Fold Change
PWY−7013: L−1,2−propanediol degradation
Superclass
Other
Amino Acids Biosynthesis
Aromatic Compounds Degradation
Carbohydrates Biosynthesis
Cofactors, Prosthetic Groups, Electron Carriers Biosynthesis
Fatty Acid and Lipid Biosynthesis
Nucleosides and Nucleotides Biosynthesis
Secondary Metabolites Biosynthesis
Proportion
0
0.2
0.4
0.6
0.8
1

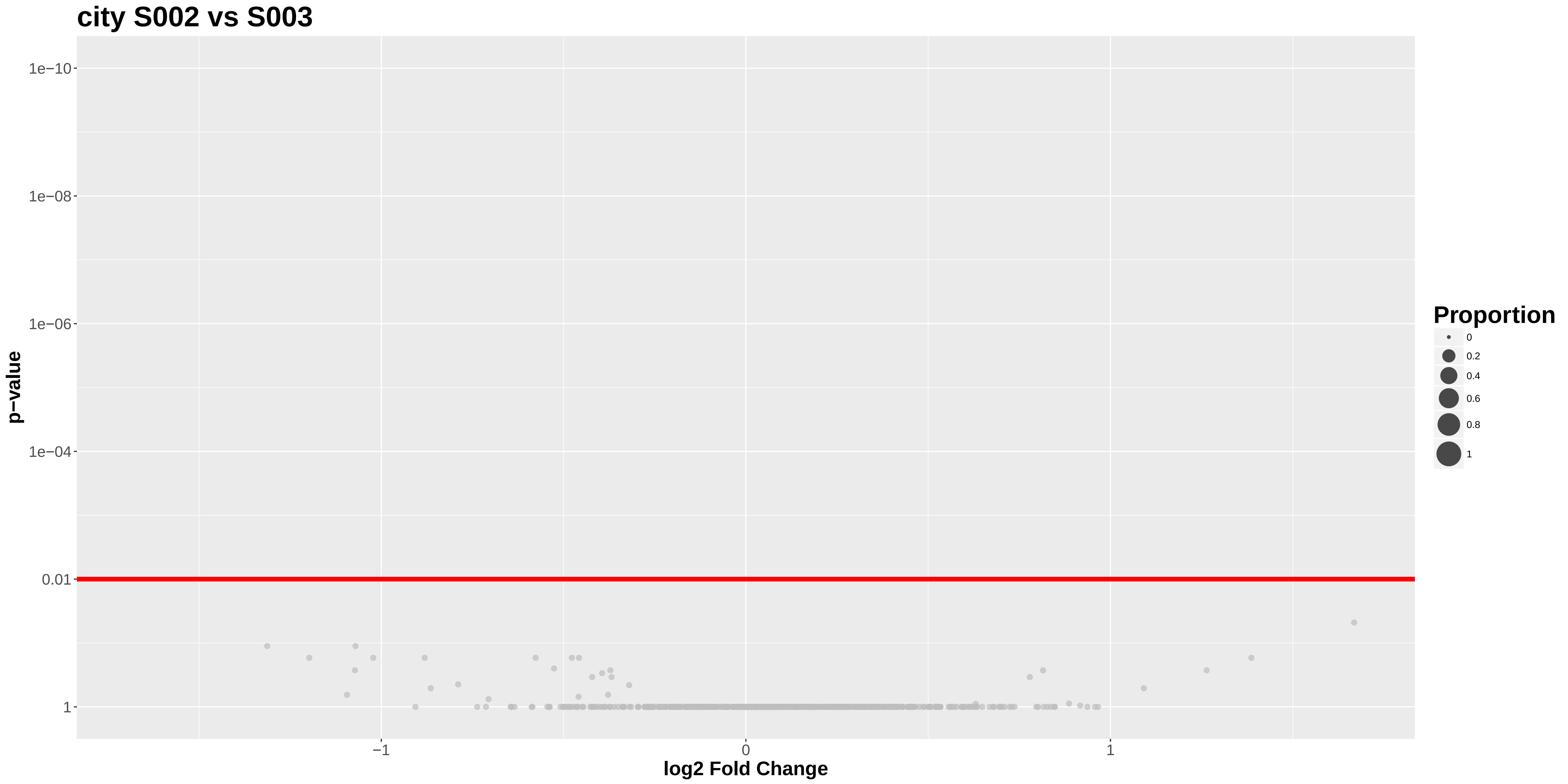
city S002 vs S003
p−value
1e−10
1e−08
1e−06
1e−04
0.01
1
−1
0
1
log2 Fold Change
Proportion
0
0.2
0.4
0.6
0.8
1

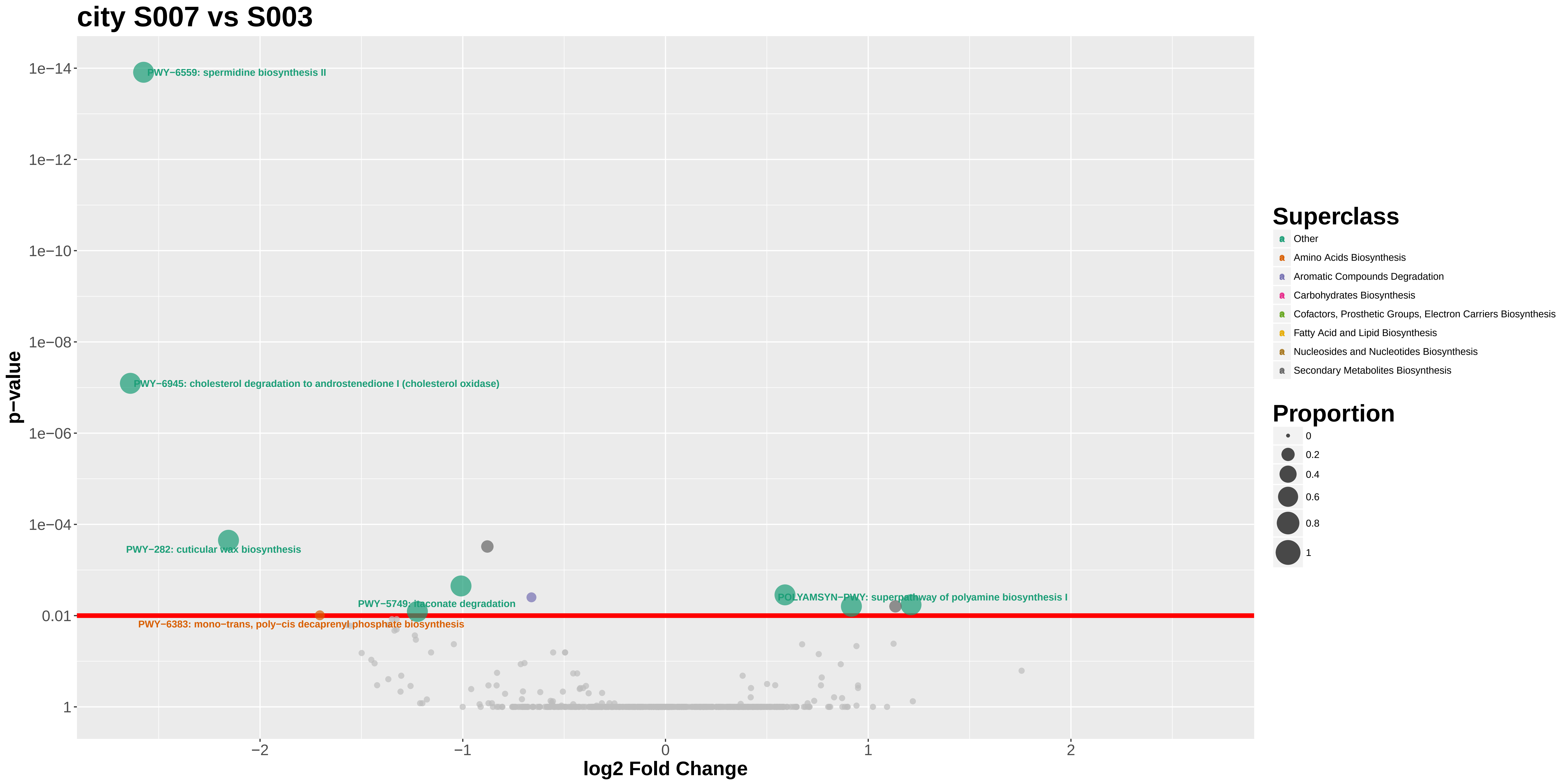

city S007 vs S003
PWY−6559: spermidine biosynthesis II
PWY−6945: cholesterol degradation to androstenedione I (cholesterol oxidase)
PWY−282: cuticular wax biosynthesis
PWY−5749: itaconate degradation
PWY−6383: mono−trans, poly−cis decaprenyl phosphate biosynthesis
POLYAMSYN−PWY: superpathway of polyamine biosynthesis I
p−value
1e−14
1e−12
1e−10
1e−08
1e−06
1e−04
0.01
1
log2 Fold Change
−2
−1
0
1
2
Superclass
Other
Amino Acids Biosynthesis
Aromatic Compounds Degradation
Carbohydrates Biosynthesis
Cofactors, Prosthetic Groups, Electron Carriers Biosynthesis
Fatty Acid and Lipid Biosynthesis
Nucleosides and Nucleotides Biosynthesis
Secondary Metabolites Biosynthesis
Proportion
0
0.2
0.4
0.6
0.8
1

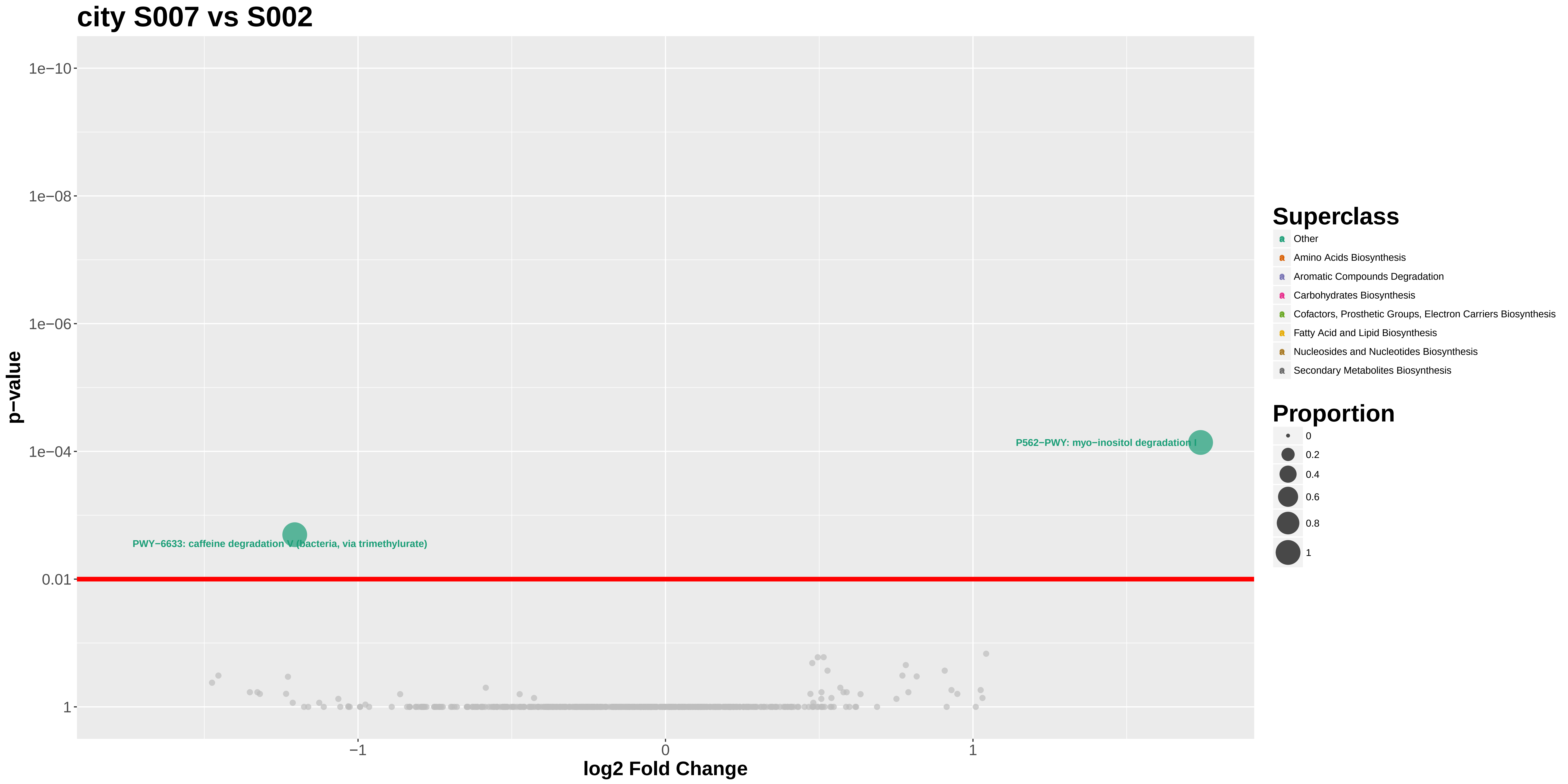

city S007 vs S002
p−value
1e−10
1e−08
1e−06
1e−04
0.01
1
log2 Fold Change
−1
0
1
PWY−6633: caffeine degradation V (bacteria, via trimethylurate)
P562−PWY: myo−inositol degradation I
Superclass
Other
Amino Acids Biosynthesis
Aromatic Compounds Degradation
Carbohydrates Biosynthesis
Cofactors, Prosthetic Groups, Electron Carriers Biosynthesis
Fatty Acid and Lipid Biosynthesis
Nucleosides and Nucleotides Biosynthesis
Secondary Metabolites Biosynthesis
Proportion
0
0.2
0.4
0.6
0.8
1
