# Supplementary material for: Metagenomic characterization of ambulances across the USA
Source: Microbiome. 2017 Sep 22;5:125. doi: 10.1186/s40168-017-0339-6 (PMC5610413; doi:10.1186/s40168-017-0339-6)
Supplement: Supplementary file 33 — Figure S14. Boxplot of alpha diversity across surfaces (Shannon index calculated based on Metaphlan2 results). Surface did not have a significant effect on alpha diversity (univariate ANOVA with surfaces N < 10 dropped: P = 0.70). (DOCX 122 kb) [file 40168_2017_339_MOESM33_ESM.docx]

Figure S14: boxplot of mean shannon diversity (metaphlan data) across surfaces
