# Supplementary material for: Metagenomic characterization of ambulances across the USA
Source: Microbiome. 2017 Sep 22;5:125. doi: 10.1186/s40168-017-0339-6 (PMC5610413; doi:10.1186/s40168-017-0339-6)

Figure S15: variation in beta diversity across surface (A), and region (B), and species correlation plot (C).

A


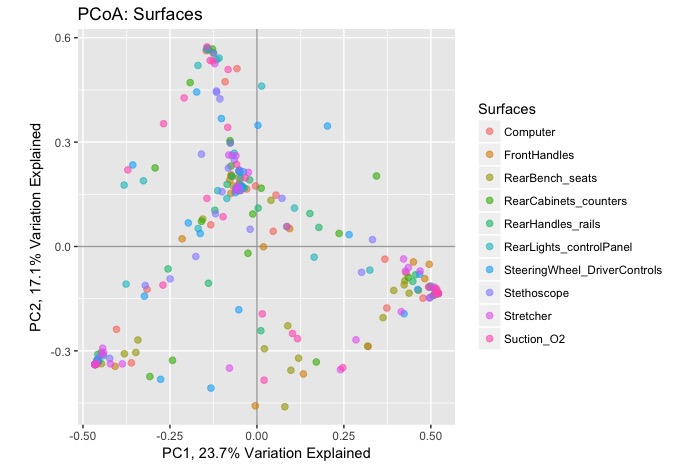


B


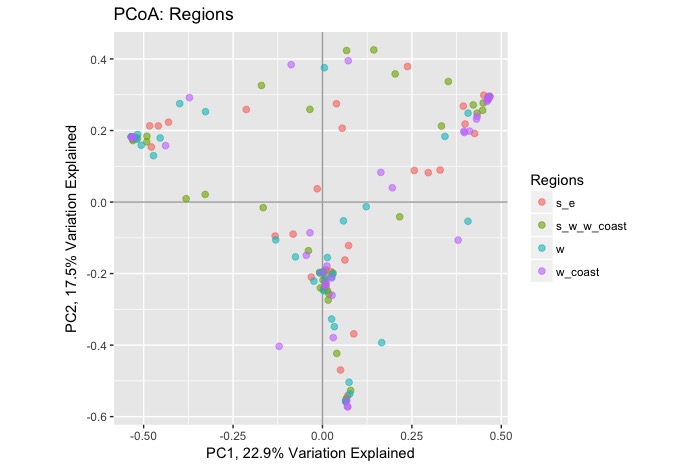


C


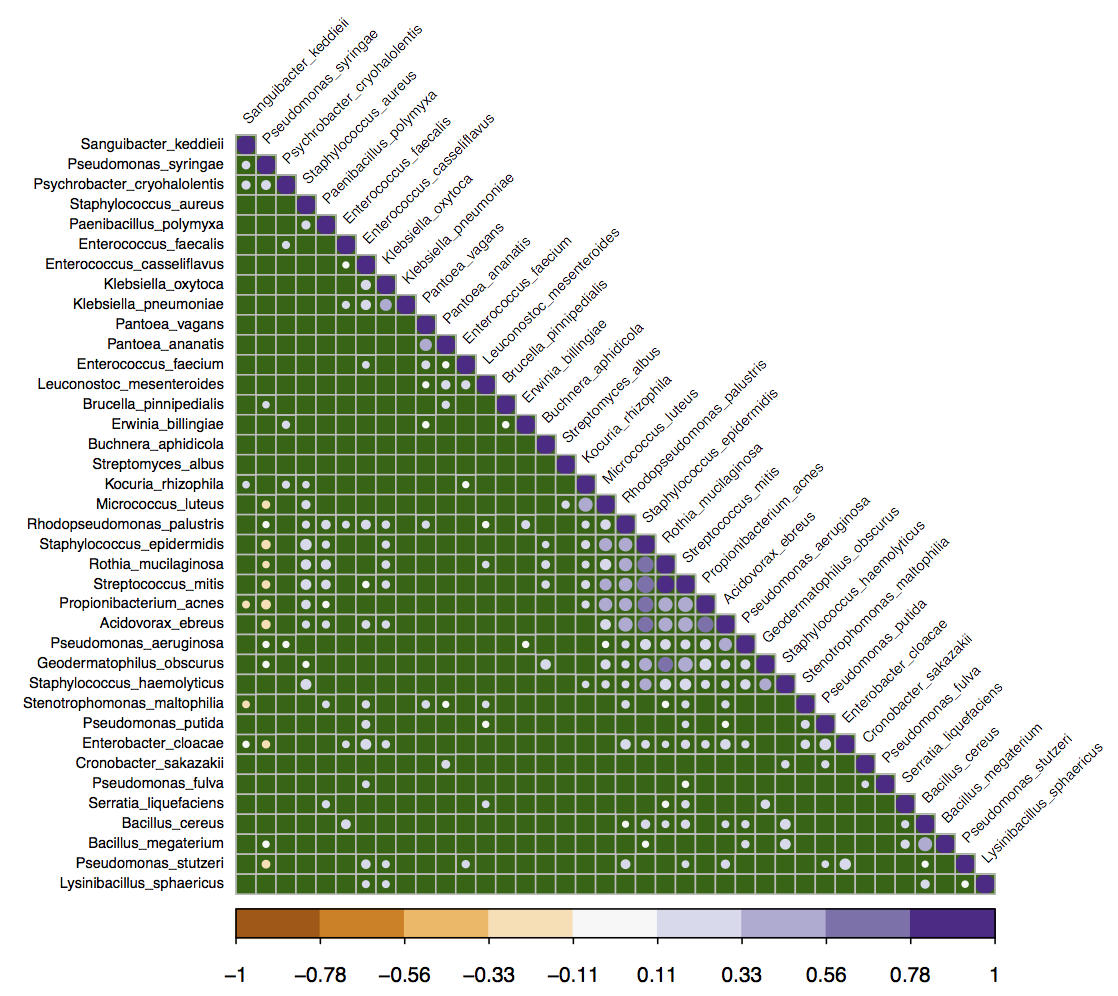

Supplement: Supplementary file 34 — Figure S15. Potential factors driving variation in beta diversity (calculated using MetaPhlAn2/CLARK overlap). Beta diversity was calculated with relative abundances, using the VEGAN package in R. Data were standardized [0,1] and balanced through random sampling (for each region, n = 36; for each surface, n = 25). (A) By surface, (B) by region, (C) Correlation plot using Spearman’s rank coefficient, including only species that had total relative abundance > 10, P < 0.05. (DOCX 552 kb) [file 40168_2017_339_MOESM34_ESM.docx]
