# Supplementary material for: Metagenomic characterization of ambulances across the USA
Source: Microbiome. 2017 Sep 22;5:125. doi: 10.1186/s40168-017-0339-6 (PMC5610413; doi:10.1186/s40168-017-0339-6)
Supplement: Supplementary file 35 — Supplemental Methods. (DOCX 32 kb) [file 40168_2017_339_MOESM35_ESM.docx]

**Supplemental Methods**:

*Sequencing*

Data was shipped to sequencer Hudson Alpha in 5 plates. Plates 1-4 were sequenced over 2 lanes of HiSeq2500 in High Output mode 125 bp PE run. The last plate of 16 samples was sequenced over 2 lanes of HiSeq2500 in Rapid Run mode 125 bp PE run.

*Data Analysis*

NOTE: Scripts are also available on Github at the following link: https://github.com/2017-ambMicrobiome/ambulance_data

Trimming and Cleaning Data

#shell script for FASTX-Toolkit

#!/bin/bash

#$ -cwd

#$ -j y

#$ -l h_rt=96:00:00

#$ -m bea

#$ -M niamh.ohara@gmail.com

#$ -N fastx_amb

#$ -pe smp 5

#$ -l os=rhel6.3

#$ -l vf=1G

#$ -l h_vmem=2G

#$ -l zenodotus=true

set -ex

#Moving files over to temp directory on cluster

rsync -a /zenodotus/masonlab/rotcollab_scratch/har2011/ambulance/FASTQ_hudson_alpha/pairedEnds_fromOriginal/*.fastq.gz $TMPDIR

cd $TMPDIR

gunzip *.gz

#Trimming using FASTX toolkit with cutoff of Q20

for f in *fastq

do

cat $f | /home/darryl/bin/fastx_toolkit/bin/fastq_quality_trimmer -v -t 20 -l 80 -Q 33 -o $f.out

done

rsync -a $TMPDIR/*.out /zenodotus/masonlab/rotcollab_scratch/nbo5/trim_amb_samples/trim_amb_Q20

#Trimming adapters using cutadapt

for f in *fastq

do

cat $f | /home/nbo5/bin/cutadapt-1.2.1/bin/cutadapt -b GATCGGAAGAGCGGTTCAGCAGGAATGCCGAG -b ACACTCTTTCCCTACACGACGCTCTTCCGATCT -b AATGATACGGCGACCACCGAGATCTACACTCTTTCCCTACACGACGCTCTTCCGATCT -b CAAGCAGAAGACGGCATACGAGATCGGTCTCGGCATTCCTGCTGAACCGCTCTTCCGATCT -b ACACTCTTTCCCTACACGACGCTCTTCCGATCT -b CGGTCTCGGCATTCCTGCTGAACCGCTCTTCCGATCT -o $f.clean -

done

rsync -a $TMPDIR/*.clean /zenodotus/masonlab/rotcollab_scratch/nbo5/trim_amb_samples/trim_amb_adapt

MetaPhlAn2 Analysis

#!/bin/bash

#$ -cwd

#$ -j n

#$ -l h_rt=96:00:00

#$ -m bea

#$ -M eba2001@med.cornell.edu

#$ -N ambulance_metaphlan

#$ -pe smp 5

#$ -l os=rhel6.3

#$ -l h_vmem=1G

rsync -a /zenodotus/masonlab/pathomap_scratch/ebrahim/Ambulance_Study/clean_up/*fastq.gz $TMPDIR

mkdir $TMPDIR/metaphlan_out

cd $TMPDIR

for file in $(find $TMPDIR/metaphlan_out *fastq.gz)

do

zcat $file | /home/ebrahim/anaconda/bin/python /home/ebrahim/bin/metaphlan2/metaphlan2.py --bowtie2db /home/ebrahim/bin/metaphlan2/db_v20/mpa_v20_m200 --bowtie2_exe /home/ebrahim/bin/bowtie2-2.1.0/bowtie2 --input_type fastq --mpa_pkl /home/ebrahim/bin/metaphlan2/db_v20/mpa_v20_m200.pkl --nproc $NSLOTS --bowtie2out $file.bt2.out > $TMPDIR/metaphlan_out/$file.out

done

rsync -a $TMPDIR/metaphlan0_out /zenodotus/masonlab/pathomap_scratch/ebrahim/Ambulance_Study/

Overlap Analysis

CLARK provides microbial identification and reports for each organism detected the NCBI taxonomy ID, name at the species/strain level and taxonomic lineage. We used a naming approach in which we took taxa names, reformatted them in the format of MetaPhlAn2 output and for each line in the clark output we selected those that matched through the equivalent MetaPhlAn2 file. Here is a list of the scripts used and logs generated during the process for both the genus and species overlap files.

#!/bin/bash

# overlap_metaph_clark.sh

# Looks for overlaping species between clark abundance output and metaphlan.

# Declaring variables

in_files=''

out_file=''

out_d=''

help_readout="\nUsage:overlap_metaph_clark.sh -i <input_files> -d <out_dir> -f <out_file>

-h --help Displays this message

-i --input Input files, separated by a comma

-d --output_dir Output directory

-f --output_file Output filename

* Long form of options require \"=\"

"

# Check for file

if [[ -z $@ ]]; then

echo -e "$help_readout"

exit 1

fi

# GetOpt

ARGS=$( getopt -o h::i:d:f: -l "help::,input:,output_dir:,output_file:" -n "overlap_metaph_clark.sh" -- "$@" );

eval set -- "$ARGS";

# extract options and their arguments into variables

while true; do

case "$1" in

-h|--help)

shift;

echo -e "${help_readout}";

exit 1;

;;

-i|--input)

shift;

if [[ -n $1 ]]; then

in_files=$1;

shift;

fi

;;

-d|--output_dir)

shift;

if [[ -n $1 ]]; then

out_d=$1;

shift;

fi

;;

-f|--output_file)

shift;

if [[ -n $1 ]]; then

out_file=$1;

shift;

fi

;;

--)

shift;

break;

;;

esac

done

# Parsing input files

in_clark=$( echo ${in_files} | cut -d ',' -f1 );

in_meta=$( echo ${in_files} | cut -d ',' -f2 );

echo -e "\nVariable echo:

Input files: ${in_files}

Input clark file: ${in_clark}

Input metaphlan file: ${in_meta}

Ouput directory: ${out_d}

Ouput file name: ${out_file}

"

# Search for overlapping species

echo "Species,Clark_rel_abun,Meta_rel_abun" > ${out_d}/${out_file}_spp

echo "Genus" > ${out_d}/${out_file}_gen

echo "tmp" > ${out_d}/${out_file}_gen.tmp

echo -e "\nProcessing files ${in_clark} and ${in_meta}."

while read -r line || [[ -n "$line" ]]; do

clark_spp_nm=$( echo "$line" | cut -d ',' -f1 );

if [[ $clark_spp_nm != "Name" && $clark_spp_nm != "UNKNOWN" ]]; then

clark_spp_nm_meta_fmt=$( echo "$clark_spp_nm" | sed -e "s: :_:g" );

overlap_chk_spp=$( egrep "s__${clark_spp_nm_meta_fmt}" ${in_meta} | head -n 1 );

clark_gen_nm_meta_fmt=$( echo "$clark_spp_nm" | cut -d ' ' -f1 );

overlap_chk_gen=$( egrep "g__${clark_gen_nm_meta_fmt}" ${in_meta} );

if [[ -n $overlap_chk_spp ]]; then

echo -e "\n\t\tFound species ${clark_spp_nm} in both files"

clark_rel_abun=$( echo "$line" | cut -d ',' -f6 );

meta_rel_abun=$( echo "$overlap_chk_spp" | cut -f2 );

echo "${clark_spp_nm},${clark_rel_abun},${meta_rel_abun}" >> ${out_d}/${out_file}_spp

fi

if [[ -n $overlap_chk_gen ]]; then

echo -e "\n\t\tFound genus ${clark_gen_nm_meta_fmt} in both files"

echo "${clark_gen_nm_meta_fmt}" >> ${out_d}/${out_file}_gen.tmp

fi

fi

done < ${in_clark}

tail -n +2 ${out_d}/${out_file}_gen.tmp | sort | uniq >> ${out_d}/${out_file}_gen

rm ${out_d}/${out_file}_gen.tmp

# End script

HUMAnN2 Analysis

#$ -e /zenodotus/masonlab/pathomap_scratch/ebrahim/Ambulance_Study/humann_output/AW0508_clean.8.2.humann2.err

#$ -m bea

#$ -M eba2001@med.cornell.edu

#$ -l zenodotus=true

#$ -l h_vmem=2G

#$ -pe smp 8

#$ -N AW0508_clean.8.2.humann2

name=AW0508_clean.8.2

out_name=AW0508_clean.humann2

input_file=/zenodotus/masonlab/pathomap_scratch/ebrahim/Ambulance_Study/clean_up/AW0508_clean.fastq.gz

cpu=8

out_dir=/zenodotus/masonlab/pathomap_scratch/ebrahim/Ambulance_Study/humann_output

rsync -av $input_file $TMPDIR/

cd $TMPDIR

export PATH=/home/emh2013/programs/jdk1.8.0_66/bin:/home/emh2013/programs/jdk1.8.0_66:/home/emh2013/anaconda/bin:/home/emh2013/programs/samtools-1.3/bin:/usr/lib64/qt-3.3/bin:/usr/kerberos/sbin:/usr/kerberos/bin:/usr/local/bin:/bin:/usr/bin:/usr/local/sbin:/usr/sbin:/sbin:/opt/bin:/opt/dell/srvadmin/bin:/home/ebrahim/bin:/home/yos2006/tools/BEDTools-Version-2.14.3/bin:/home/emh2013/scripts/bioinfo_scripts/python:/home/emh2013/scripts/bioinfo_scripts/perl:/home/emh2013/scripts/bioinfo_scripts/awk:/home/emh2013/scripts/bioinfo_scripts/bash:/home/emh2013/scripts/bioinfo_scripts/scripts_CRG:/home/emh2013/dev/jitterbug-code:/home/emh2013/dev/jitterbug-code/jip_scripts/:/home/emh2013/dev/jitterbug-code/scripts/:/home/emh2013/programs/htop-1.0/bin/bin:/home/emh2013/programs/FastQC:/home/emh2013/programs/lumpy-sv/scripts:/home/emh2013/programs/lumpy-sv/bin:/home/emh2013/programs/IGVTools:/home/emh2013/programs/breakdancer-1.1_2011_02_21/cpp:/home/emh2013/programs/scalpel-0.3.1:/home/emh2013/programs/ncbi-blast-2.2.30+/bin:/home/emh2013/programs/last-572/src:/home/emh2013/programs/last-572/scripts:/home/emh2013/programs/nanopore-scripts:/home/emh2013/programs/cdhit:/home/emh2013/programs/biobakery-shortbred-ddce9103c5ee:/home/emh2013/programs:/home/emh2013/programs/graphlan:/home/emh2013/programs/metaphlan2/utils:/home/emh2013/programs/metaphlan2/utils/export2graphlan:/home/emh2013/programs/metaphlan2:/home/emh2013/programs/lefse:/home/emh2013/programs/bowtie2-2.2.5:/home/emh2013/programs/humann2_v0.2.0/humann2/tools:/home/emh2013/programs/htslib-1.3/bin

echo "name: $name"

date=$(date)

echo " starting humann2 $name ... $date" >> /home/ebrahim/JOB_LOG.txt

humann2 --input $input_file --threads $cpu --remove-temp-output --output-basename $out_name --output .

date=$(date)

echo "DONE humann2 $name ... $date" >> /home/ebrahim/JOB_LOG.txt

echo "files generated:"

ls -l

rsync -av $out_name* $out_dir/

*Staphylococcus aureus* Coverage Analysis

#bedtools to calculate coverage across the genome of S. aureus for one S. aureus positive file

#!/bin/bash

echo `/home/darryl/bin/bedtools2-2.19.1/bin/coverageBed -d -abam /zenodotus/masonlab/rotcollab_scratch/nbo5/hai_work/s_aureus/AW0713_pe.sorted.bam -b /zenodotus/masonlab/rotcollab_scratch/nbo5/hai_work/s_aureus/reference/GCA_000013465.1_ASM1346v1_genomic.gff.gz > s_aureus_AW0713.cover.txt`

#pull out coverage including average coverage, variance and standard deviation from bedtools coverage files.

#!/bin/bash

for i in /zenodotus/masonlab/rotcollab_scratch/nbo5/hai_work/coverage_bedtools/s_aureus/coverage/*.txt; do

grep "mecA" $i > $i.mecA.txt

done

#Just pulling out the gene

#meca - repeat for all the target genes

#!/bin/bash

#saving lines that have a mecA and Gene hit

for i in /zenodotus/masonlab/rotcollab_scratch/nbo5/hai_work/coverage_bedtools/s_aureus/coverage/*.txt; do

grep "mecA" $i | grep "Gene" > $i.mecA.txt

done

#Then run from within the scripts direct /zenodotus/masonlab/rotcollab_scratch/nbo5/scripts

./get_coverage_meca.sh

#Then get average and SD and SE for the last column - the coverage column

CARD Analysis

#!/bin/bash

# 20160805_parallel_custom_abundance_ebr.sh

# Create directories

export tmp=/scratchLocal/cmlab/har2011/tmp_2

cd $tmp

mkdir raw_f

mkdir ref_f

mkdir abun

export ref_f=${tmp}/ref_f

export raw_f=${tmp}/raw_f

export abun_o=${tmp}/abun

export script_d=/home/har2011/software/CLARKSCV1.2.2-b

# Sync files generated from clark using custom CARD database

parallel -j 24 --gnu rsync -av {1} ${raw_f} ::: $( find /zenodotus/masonlab/rotcollab_scratch/har2011/metagenomics/CLARK_out/clark_card_ambulance/31kmer/*.gz )

parallel -j 24 --gnu gunzip {1} ::: $( find ${raw_f}/*.gz )

# Create file reference

touch ${ref_f}/clarkOut.fofn

find ${raw_f}/*.csv >> ${ref_f}/clarkOut.fofn

# Process files

parallel -k -j 24 --gnu ${script_d}/JR_estimate_abundance_all.sh -i {} -d ${abun_o} -o {/}_abundance.csv -s 50 -t /zenodotus/masonlab/rotcollab_scratch/har2011/DB_files/clark_virulence_db/targets_finished.txt :::: ${ref_f}/clarkOut.fofn

# Rsync files back to abundance directory

parallel -j 24 --gnu rsync -av {1} /zenodotus/masonlab/rotcollab_scratch/har2011/metagenomics/CLARK_abundance_out/clark_card_ambulance/31kmer/score_50_all ::: $( find ${abun_o}/*.csv )

rm -r ${tmp}/*

Alpha Diversity Analysis in R

library("vegan")

library(stringr)

library(plyr)

library(xtable)

# import data, this tsv file has header and firs # removed IOT for importation and is also removed taxonomy data (now stored as OTU)

amb_data <- read.table("/home/jake/ambulance/overlap_diversity/spp_only_amb.biom.tsv", sep = "\t", header = TRUE, numerals = c("allow.loss"), colClasses = "numeric")

x1 <- data.frame(t(amb_data))

x1_dim <- matrix(dim(x1))

# Reformats each value in the matrix to an integer

for (i in 1:x1_dim[1,1])

{

  for (j in 1:x1_dim[2,1])

  {

    x1[i,j] <- as.integer(x1[i,j])

  }

}

# Diversity shannon-weaver index

div_amb_simp <- matrix(diversity(x1, index = "simpson"))

div_amb_shan <- matrix(diversity(x1, index = "shannon"))

div_amb_stat <- matrix(c(mean(div_amb_simp),sd(div_amb_simp), mean(div_amb_shan),sd(div_amb_shan)), nrow = 2, ncol = 2)

# Creating table

div_amb_stat <- round(div_amb_stat, 3)

rownames(div_amb_stat) <- c("Mean", "SD")

colnames(div_amb_stat) <- c("Simpsons", "Shannon")

div_amb_stat

sink("/home/jake/amb_stat_overlap.tex", append = TRUE, split = TRUE)

xtable(div_amb_stat)

# Create csv table with shannon and simpsons diversity indices

div_amb_simp_r <- round(div_amb_simp, 3)

div_amb_shan_r <- round(div_amb_shan, 3)

x2 <- as.matrix(row.names(x1))

x3 <- cbind(x2[2:398,], div_amb_simp_r[2:398,], div_amb_shan_r[2:398,])

colnames(x3) <- c("Sample.ID", "Simpsons.index", "Shannon.index")

write.csv(x3, file = "Diversity.indices.overlap.csv", quote = FALSE, row.names = FALSE)

Beta Diversity Analysis in R

require(tidyr)

require(vegan)

require(reshape)

require(dplyr)

require(ape)

require(ade4)

require(ggplot2)

require(ape)

require(ecodist)

require(ade4)

require(phyloseq)

library("devtools")

require(phyloseq)

require(PerformanceAnalytics)

install.packages("corrplot")

require(corrplot)

require(Hmisc)

require(readr)

library(RColorBrewer)

## Data prep/clean

ambdata_jun16 <- read_csv("~/Dropbox/Ambulance_Study/data_sample_info/metadata_compiled/ambulance_metaphlan_hais_diversity_june_2016.csv")

amb.env <- ambdata_jun16[,1:20]

rownames(amb.env) <- amb.env$sample.ID

# move overlap files (in folder spp_outlap_files_meta_clark_v1) to local wd or set wd to dropbox: ~/Dropbox/Ambulance_Study/results/overlap_meta_clark/v1/spp_outlap_files_meta_clark_v1

files <- list.files(pattern="*_spp")

olf <- NULL

for (f in files) {

  dat <- read.csv(f, header=F, sep=",", na.strings="", colClasses="character")

  dat$file <- unlist(strsplit(f,split=".",fixed=T))[1]

  olf <- rbind(olf, dat)

}

colnames(olf) <- c("Species","Clark","Meta", "File")

meta.olf <- olf[,-2] #remove clark

# make wide instead of long

meta.olf.2 <- reshape(meta.olf, timevar = "Species", idvar = "File", direction = "wide")

rownames(meta.olf.2) <- meta.olf.2$File

meta.olf.3 <- meta.olf.2[,-2]

colnames(meta.olf.3)[1] <- "sample.ID"

meta.olf.3$sample.ID <- gsub("_clean", "", meta.olf.3$sample.ID)

rownames(amb.env) <- amb.env[,1]

## merge the env data from full dataset with the olf to get env data for overlap

olf.env <- merge(meta.olf.3, amb.env, by = "sample.ID", all.x = FALSE)

rownames(olf.env) <- olf.env[,1]

meta.olf.4 <- olf.env[,2:128]

meta.olf.4[is.na(meta.olf.4)] <- 0 #change NAs to O

#

meta.olf.5 <- apply(meta.olf.4, 2, as.numeric) #change to numeric data -- might not be necessary

rownames(meta.olf.5) <- rownames(olf.env)

rownames(meta.olf.5) <- gsub("_clean", "", rownames(meta.olf.5))

colnames(meta.olf.5) <- gsub("Meta.", "", colnames(meta.olf.5))

colnames(meta.olf.5) <- gsub("\\s+","_",colnames(meta.olf.5))

meta.full <- cbind(meta.olf.5, olf.env[,129:ncol(olf.env)]) #combine env DF with spp DF

#add front/back (location of sampled surface) column

meta.full$front <- NA # add column for front/back; front = 1, back = 0

meta.full$front[meta.full$sample.Surface == "SteeringWheel_DriverControls" | meta.full$sample.Surface == "Computer" | meta.full$sample.Surface == "FrontHandles"] <- 1

meta.full$front[is.na(meta.full$front)] <- 0

meta.full$fact.front <- factor(meta.full$front, labels = c("back", "front"))

# remove the "after" cleaning status

meta.full.1 <- meta.full[-grep("AFTER",meta.full$sample.Clean_status),]

meta.spp.1 <- meta.full.1[,1:127]

##########

overlap <- read_csv("~/Dropbox/Ambulance_Study/results/overlap_meta_clark/overlap_v1_metaphlan.csv")

spp <- overlap[,2:128]

sim <- with(overlap, simper(spp, sample.Surface))

# summary(sim)

par(mar=c(2,2,2,2))

spp <- data.frame(spp)

spp.l <- apply(spp,1,log)

spp.l <- t(spp.l)

spp.2 <- spp[,colSums(spp)>10]

# chart.Correlation(spp.2, histogram=TRUE, pch=19)

# pearson's  and spearman's

pcor <- cor(spp.2, method = "pearson")

scor <-  cor(spp.2, method = "spearman")

# get pvals, plot (green=NA)

# pearson, log-transform

spp.3 <- log(spp.2+1)

pclog <- rcorr(as.matrix(spp.3), type = "pearson")

rlog <- pclog$r

plog <- pclog$P

corrplot(rlog, type = "lower", order = "hclust", p.mat = plog,

         sig.level = 0.05, insig = "blank",

         tl.col="black", tl.cex = .6, tl.srt =45,

         col = brewer.pal(n = 9, name = "PuOr"), bg = "darkgreen")

#pearson, no transform

pcp <- rcorr(as.matrix(spp.2), type = "pearson")

M <- pcp$r

p_mat <- pcp$P

corrplot(M, type = "lower", order = "hclust", p.mat = p_mat, sig.level = 0.05, insig = "blank",

         tl.col="black", tl.cex = .6, tl.srt =45, col = brewer.pal(n = 9, name = "PuOr"), bg = "darkgreen")

#spearman - best option for these data

scp <- rcorr(as.matrix(spp.2), type = "spearman")

R <- scp$r

p_matp <- scp$P

corrplot(R, type = "lower", order = "hclust", p.mat = p_matp, sig.level = 0.05, insig = "blank",

        tl.col="black", tl.cex = .6, tl.srt =45, col = brewer.pal(n = 9, name = "PuOr"), bg = "darkgreen")

corrplot(R, type = "upper", order = "hclust", p.mat = p_matp, sig.level = 0.05, insig = "blank",

         tl.col="black", tl.cex = .6, tl.srt =45, col = brewer.pal(n = 9, name = "PuOr"), bg = "darkgreen")

## flatten output

flattenCorrMatrix <- function(cormat, pmat) {

  ut <- upper.tri(cormat)

  data.frame(

    row = rownames(cormat)[row(cormat)[ut]],

    column = rownames(cormat)[col(cormat)[ut]],

    cor  =(cormat)[ut],

    p = pmat[ut]

  )

}

cms <- flattenCorrMatrix(scp$r, scp$P)

cms2 <- filter(cms, abs(cor > .5), p <= .05)

lowcor <- filter(cms, abs(cor<.11),  p <= .05)

View(cms2)

View(lowcor)

summary(cms2)

summary(lowcor)

#check out some plots: s>p for most

strep <- spp$Streptococcus_mitis

acid <- spp$Acidovorax_ebreus

plot(strep, acid)

cmp <- flattenCorrMatrix(pcp$r, pcp$P)

cmp2 <- filter(cmp, abs(cor > .5), p < .05)

View(cmp2)

prop <- spp$Propionibacterium_acnes

geo <- spp$Geodermatophilus_obscurus

roth <- spp$Rothia_mucilaginosa

strep <- spp$Streptococcus_mitis

plot(prop, roth)

plot(prop, geo)

plot(strep, roth)

cor(roth, geo)

cor(strep, roth)

# kendall's rho

kcor <- cor(spp, method = "kendal")

corrplot(kcor, method = "circle", type="upper", order="hclust", tl.col="black", tl.cex = .8, tl.srt=45)

##### PCoA -- balance data, remove ec, etc

##Region

#see which regions have small n

reg <- overlap  %>% group_by(region)

sum.reg <- summarise(reg, samp.n = n())

##remove eastcoast

no.ec<- overlap[-grep("e_coast",overlap$region ),]

## sample from regions n=36

sampled.region <- no.ec %>% group_by(region) %>% sample_n(size = 36, replace=FALSE)

sampled.spp <- sampled.region[,2:128]

sampled.spp <- data.frame(sampled.spp)

spp.hel <- decostand(sampled.spp, "range", MARGIN = 1) # standardize rows [0,1]

volf.sn <- vegdist(spp.hel)

volf.sn[is.na(volf.sn)] <- 0

mds.volf.sn <- dudi.pco(volf.sn, scannf=F)

VariationExplainedPC1 <- mds.volf.sn$eig[1]/sum(mds.volf.sn$eig)

VariationExplainedPC2 <- mds.volf.sn$eig[2]/sum(mds.volf.sn$eig)

#set up plot

ppp <- ggplot() + coord_fixed() +

  labs(x="Comp1, Axis1", y="Comp2, Axis2") +

  geom_hline(yintercept=0, col="darkgrey") +

  geom_vline(xintercept=0, col="darkgrey")

# make the scree plot in a viewport

myscree <- function(eigs, x=0.8, y=0.1, just=c("right","bottom")){

  vp <- viewport(x=x, y=y, width=0.2, height=0.2, just=just)

  sp <- qplot(factor(1:length(eigs)), eigs,

              geom="bar", stat="identity") +

    labs(x = NULL, y = NULL)

  print(sp, vp=vp)

}

ppp + geom_point(data=data.frame(mds.volf.sn$li, sample_data(data.frame(sampled.region))),

                      aes(x=A1, y=A2, col=sampled.region$region), size = 2, alpha=.6) +

  labs(title="PCoA: Regions") + scale_fill_hue(c=45, l=80) + xlab("PC1, 22.9% Variation Explained")  + ylab("PC2, 17.5% Variation Explained") + labs(colour = "Regions")

VariationExplainedPC1

VariationExplainedPC2

##Surfaces

#see what surfaces have low n

surf <- overlap %>% group_by(sample.Surface)

sum.surf <- summarise(surf, samp.n = n())

surfs <- filter(sum.surf, samp.n > 20)

surf.names <- as.character(surfs$sample.Surface)

overlap.2 <- overlap[overlap$sample.Surface %in% surf.names,]

## sample from surfaces n=27

sampled.ol <- overlap.2 %>% group_by(sample.Surface) %>% sample_n(size = 27, replace=FALSE)

sampled.spp <- sampled.ol[,2:128]

spp.3 <- decostand(sampled.spp, "range", MARGIN = 1) #standardized row values [0,1]

#####

volf.sn.2 <- vegdist(spp.3)

volf.sn.2[is.na(volf.sn.2)] <- 0

mds.volf.sn.2 <- dudi.pco(volf.sn.2, scannf=F)

VariationExplainedPC1 <- mds.volf.sn.2$eig[1]/sum(mds.volf.sn.2$eig)

VariationExplainedPC2 <- mds.volf.sn.2$eig[2]/sum(mds.volf.sn.2$eig)

VariationExplainedPC1

VariationExplainedPC2

ppp + geom_point(data=data.frame(mds.volf.sn.2$li, sample_data(data.frame(sampled.ol))),

                      aes(x=A1, y=A2, col=sampled.ol$sample.Surface), size = 2, alpha=.6) +

  labs(title="PCoA: Surfaces") + scale_fill_hue(c=45, l=80) + xlab("PC1, 23.7% Variation Explained") + ylab("PC2, 17.1% Variation Explained") + labs(colour = "Surfaces")

#front/back

ppp + geom_point(data=data.frame(mds.volf.sn.2$li, sample_data(data.frame(sampled.meta.2))),

                 aes(x=A1, y=A2, col=sampled.meta.2$fact.front), size = 2, alpha=.6) +

  labs(title="PCoA: Front vs Back of Ambulances") + scale_fill_hue(c=45, l=80)

#### permanova

a <- vegdist(spp.2)

a[is.na(a)] <- 0

perma.1 <- adonis(a~region, data = overlap, permutations = 2000)

perma.2 <- adonis(a~sample.Surface, data = overlap, permutations = 2000)

perma.3 <- adonis(a~region*sample.Surface, data = overlap, permutations = 2000)

round(perma.2$aov.tab[1:6], 4)

round(perma.1$aov.tab[1:6], 4)

Class Classification and Analysis R

We attempted to maximize classification performance of the taxonomic dataset by evaluating an array of classifiers on the training-validation set. For overlap, RF yielded the best mean balanced accuracy (Q1=0.5265, Q3=0.5757) and kappa (0.0505, 0.1326) for classifying sample surface during cross validation, although no classifier performed particularly well (**Figure S16**). There was a modest improvement in RF performance using the MetaPhlAn2 dataset (0.5341, 0.5804 and 0.0735 and 0.1577 for mean balanced accuracy and kappa, respectively); however, the results were generally consistent with overlap (**Figure S17**). Predicting region, on the other hand, resulted is much better performance, particularly for MetaPhlAn2 data, with, in terms of mean balanced accuracy, gradient boosting (0.6705, 0.7391) and RF (0.6571, 0.7337) performing best (**Figure S18-S19**). When region was split into smaller city groups, there was a clear performance drop (**Figure S20-S21**).

These results led us to utilize RF to evaluate classification in terms of input dataset. There was negligible difference in performance for classifying sample surface (**Figure S22**). Interestingly, for region, although there was clear improvement, the overlap dataset performed considerably worse compared to the other three datasets (**Figure S23**). Notably, the mean balanced accuracy IQR of the functional abundance tables (Megan and HUManN2) was smaller (0.6457, 0.7227 and 0.6394, 0.7218) than either taxonomic table (0.6287, 0.7253 and 0.5905, 0.6707 for MetaPhlAn2 and MetaPhlAn2/CLARK overlap, respectively). Classification with respect to city again led to worse performance than region, especially for the overlap dataset (**Figure S24**).

Given the challenge of multi-class classification problems with unbalanced data and few examples, we focused on the classification performance of the RF for classifying individual surfaces (1-against-all). Because we aimed to detect influential taxa, we utilized only the overlap dataset and ranked taxa in terms of RF importance. The overlap dataset was used to facilitate interpretability given that these taxa are of high confidence (i.e. detected by both MetaPhlAn2 and CLARK). The mean ROC score and balanced accuracy during cross validation across surface classes was poor: 0.5735 and 0.5480, respectively. Rear Bench Seats, Rear Lights Control Panel, and Stethoscope had the best balanced accuracies with 0.7121, 0.5957, and 0.5657 for the best RF model. There was a performance drop during testing, however, for Rear Bench Seats but not Rear Lights Control Panel or Stethoscope, with balanced accuracies of 0.5319, 0.6226, and 0.6513, respectively.

We assessed the performance binary Front-Rear surface classes; however, the performance remained poor with ROC scores of 0.5678 and 0.5205 during cross validation and testing, respectively (**Figure S25**).

#example script for surface on metaphlan data

# classification workflow

# metaphlan

# multiclass: surface

# assess classifiers on training data repeated CV

# 60 cores to parallelize

ncores <- 60

cl <- makeCluster(ncores,type='SOCK')

registerDoSNOW(cl)

# metaphlan data

OTU <- OTU_METAPHLAN

META <- META_METAPHLAN

# filter surfaces with N<=20

sMETA <- META[rownames(OTU),] %>%

  dplyr::select(id=SampleID,surface=sample.Surface,region=region,FR=FR,IE=IE) %>%

  group_by(surface) %>%

  filter(n() > 20)

sOTU <- OTU[sMETA$id,]

FILT <- (colSums(sOTU>0) < 3) # so no empties in test set

sOTU <- sOTU[,!FILT]

sOTU <- sOTU[rowSums(sOTU) > 0,]

sMETA <- sMETA %>% filter(id %in% rownames(sOTU))

Y <- as.factor(sMETA$surface)

# quantile normalized features

qOTU <- apply(sOTU,2,qnormalize)

# center/scale features

zOTU <- apply(sOTU,2,znormalize)

Xz <- zOTU[sMETA$id,]

Xq <- qOTU[sMETA$id,]

names(Y) <- sMETA$id

set.seed(78)

# make 80/20 training/testing datasets

p <- .8

train_idx <- createDataPartition(Y,times=1,p=p)$Resample1

train_names <- names(Y)[train_idx]

train_Y <- Y[names(Y) %in% train_names]

test_Y <- Y[!(names(Y) %in% train_names)]

X <- Xz #Xz

train_X <- X[rownames(X) %in% train_names,]

test_X <- X[!(rownames(X) %in% train_names),]

# test classifiers on training set

# 10-fold cv, repeated 10 times with down sampling to overcome class imbalance

nmin <- table(train_Y)

tr_ctrl <- trainControl(method='repeatedCV', number=10, repeats=10,

                        classProbs=TRUE,

                        summaryFunction=multiClassSummary,

                        sampling='down',

                        allowParallel=TRUE)

# rf: sweep number of sampled features at each split

# 128 trees, maximize mean roc of multiclass classification

param_sweep <- expand.grid(mtry=floor(seq(ncol(train_X)^.25,ncol(train_X)^.75,length=5)))

out_rf <- train(x=train_X,

                y=train_Y,

                method='rf',

                ntree=128,

                tuneGrid=param_sweep,

                metric='Mean_ROC',

                maximize=TRUE,

                trControl=tr_ctrl)

# en: sweep en and sparsity parameters

param_sweep <- expand.grid(alpha=c(.1,.5,.9),lambda=c(.0005,.005,.05))

out_en <- train(x=train_X,

                y=train_Y,

                method='glmnet',

                tuneGrid=param_sweep,

                maxit=1000000,

                metric='Mean_ROC',

                maximize=TRUE, #FALSE,

                trControl=tr_ctrl)

# regularized rf

param_sweep <- expand.grid(mtry=floor(seq(ncol(train_X)^.25,ncol(train_X)^.75,length=3)),coefReg=c(.1,.5,.9))

out_rrf <- train(x=train_X,

                 y=train_Y,

                 method='RRFglobal',

                 ntree=128,

                 tuneGrid=param_sweep,

                 metric='Mean_ROC',

                 maximize=TRUE, #FALSE,

                 trControl=tr_ctrl)

# linear svm

out_svmlinear <- train(x=train_X,

                       y=train_Y,

                       method='svmLinear',

                       metric='Mean_ROC',

                       maximize=TRUE, #FALSE,

                       trControl=tr_ctrl)

# rbf svm

out_svmrbf <- train(x=train_X,

                    y=train_Y,

                    method='svmRadial',

                    metric='Mean_ROC',

                    maximize=TRUE, #FALSE,

                    trControl=tr_ctrl)

# gradient boosting

out_gbm <- train(x=train_X,

                 y=train_Y,

                 method='gbm',

                 metric='Mean_ROC',

                 maximize=TRUE, #FALSE,

                 trControl=tr_ctrl)

# partial least squares

out_pls <- train(x=train_X,

                 y=train_Y,

                 method='pls',

                 metric='Mean_ROC',

                 maximize=TRUE, #FALSE,

                 trControl=tr_ctrl)

# k nearest neighbors

out_knn <- train(x=train_X,

                 y=train_Y,

                 method='kknn',

                 metric='Mean_ROC',

                 maximize=TRUE, #FALSE,

                 trControl=tr_ctrl)

# c5.0 decision tree

out_c50 <- train(x=train_X,

                 y=train_Y,

                 method='C5.0Tree',

                 metric='Mean_ROC',

                 maximize=TRUE, #FALSE,

                 trControl=tr_ctrl)

stopCluster(cl)

# assess model performance on training/validation set CV

models <- list(rf=out_rf,rrf=out_rrf,en=out_en,svmrbf=out_svmrbf,svmlinear=out_svmlinear,

               gbm=out_gbm,pls=out_pls,knn=out_knn,c50=out_c50)

resample_models <- resamples(models)

summary(resample_models,metric=c('Kappa','Mean_Balanced_Accuracy'))

saveRDS(list(models=models,train_X=train_X,train_Y=train_Y,test_X=test_X,test_Y=test_Y),file.path(out_path,'ml_metaphlan.rds'))

pdf(file.path(out_path,'ml_metaphlan.pdf'),height=6,width=10)

bwplot(resample_models,metric=c('Kappa','Mean_Balanced_Accuracy'))

dev.off()

rm(list=ls()[grepl('out_',ls()) & ls() != 'out_path'])

GLMM AMR and Alpha diversity models in R

library(lme4)

#bringing data in

amb_data <- read.table("/Users/niamhohara/Dropbox/Biotia/analysis/Ambulance/data_compiled/ambulance_metaphlan_hais_diversity_june_2016_R.txt", header = TRUE)

amb_data[1:3,]

#Modeling Shannon index

#running full model

full_amb <- lmer(Shannon.index_metaphlan ~ sample.Surface + sample.City + latitude + mean_temp_F + total_precip_inches + (1|region), data = amb_data, REML = FALSE)

summary(full_amb)

#then rerunning with highly correlated variables removed

full_short <- lmer(Shannon.index_metaphlan ~ sample.Surface + sample.City + mean_temp_F + (1|region), data = amb_data, REML = FALSE, na.action = "na.omit")

summary(full_short)

#running model with each variable removed in order to compare to full short model

full_short_no_surf <- lmer(Shannon.index_metaphlan ~ sample.City + mean_temp_F + (1|region), data = amb_data, REML = FALSE)

summary(full_short_no_surf)

full_short_no_city <- lmer(Shannon.index_metaphlan ~ sample.Surface + mean_temp_F + (1|region), data = amb_data, REML = FALSE)

summary(full_short_no_city)

full_short_no_temp <- lmer(Shannon.index_metaphlan ~ sample.Surface + sample.City + (1|region), data = amb_data, REML = FALSE, na.action = "na.omit")

summary(full_short_no_temp)

#comparing models using ANOVA

anova(full_short, full_short_no_surf)

anova(full_short, full_short_no_city)

anova(full_short, full_short_no_temp)

#Determing goodness of fit for mixed models - pseudo R2

library(MuMIn)

r.squaredGLMM(full_amb)

#Get effect sizes and standard error

summary(full_amb)

#modeling AMR

#bring data in

amb_amr_counts <- read.table("/Users/niamhohara/Dropbox/Biotia_Niamh/analysis/Ambulance/data_compiled/ambulance_metadata_december_2016_amr_R_NA_temp_dropped.txt", header = TRUE)

amb_amr_counts[1:2,]

#run model with correlated variables removed

full_short <- lmer(amr_count_plus_1_log ~ sample.Surface + sample.City + mean_temp_F + (1|region), data = amb_amr_counts, REML = FALSE, na.action = "na.omit")

summary(full_short)

#running model with each variable removed in order to compare to full short model full_short_no_surf <- lmer(amr_count_plus_1_log ~ sample.City + mean_temp_F + (1|region), data = amb_amr_counts, REML = FALSE)

summary(full_short_no_surf)

full_short_no_city <- lmer(amr_count_plus_1_log ~ sample.Surface + mean_temp_F + (1|region), data = amb_amr_counts, REML = FALSE)

summary(full_short_no_city)

full_short_no_temp <- lmer(amr_count_plus_1_log ~ sample.Surface + sample.City + (1|region), data = amb_amr_counts, REML = FALSE, na.action = "na.omit")

summary(full_short_no_temp)

#run anovas to compare the models

anova(full_short, full_short_no_surf)

anova(full_short, full_short_no_city)

anova(full_short, full_short_no_temp)

#Determing goodness of fit for mixed models - pseudo R2

library(MuMIn)

r.squaredGLMM(full_amb)

#Do the following to get effect sizes and standard error

summary(full_amb)
